# The first *Caipirasuchus* (Mesoeucrocodylia, Notosuchia) from the Late Cretaceous of Minas Gerais, Brazil: new insights on sphagesaurid anatomy and taxonomy

**DOI:** 10.7717/peerj.5594

**Published:** 2018-09-05

**Authors:** Agustín G. Martinelli, Thiago S. Marinho, Fabiano V. Iori, Luiz Carlos B. Ribeiro

**Affiliations:** 1 Instituto de Geociencias, Universidade Federal do Rio Grande do Sul, Porto Alegre, Rio Grande do Sul, Brazil; 2 Centro de Pesquisas Paleontológicas L. I. Price, Complexo Cultural e Científico Peirópolis, Pró-Reitoria de Extensão Universitária, Universidade Federal do Triangulo Mineiro, Uberaba, Minas Gerais, Brazil; 3 CONICET-Sección Paleontologia de Vertebrados, Museo Argentino de Ciencias Naturales “Bernardino Rivadavia”, Buenos Aires, Argentina; 4 Departamento de Ciências Biológicas, Universidade Federal do Triângulo Mineiro, Instituto de Ciências Exatas, Naturais e Educação, Uberaba, Minas Gerais, Brazil; 5 Museu de Paleontologia “Prof. Antonio Celso de Arruda Campos”, Monte Alto, Sao Paulo, Brazil

**Keywords:** Sphagesauridae, Adamantina formation, Triângulo mineiro, Bauru group, Brazil

## Abstract

Field work conducted by the staff of the Centro de Pesquisas Paleontológicas Llewellyn Ivor Price of the Universidade Federal do Triângulo Mineiro since 2009 at Campina Verde municipality (MG) have resulted in the discovery of a diverse vertebrate fauna from the Adamantina Formation (Bauru Basin). The baurusuchid *Campinasuchus dinizi* was described in 2011 from Fazenda Três Antas site and after that, preliminary descriptions of a partial crocodyliform egg, abelisaurid teeth, and fish remains have been done. Recently, the fossil sample has been considerably increased including the discovery of several, partially articulated fish remains referred to Lepisosteiformes and an almost complete and articulated skeleton referred to a new species of *Caipirasuchus* (Notosuchia, Sphagesauridae), which is the main subject of this contribution. At present, this genus was restricted to the Adamantina Formation cropping out in São Paulo state, with the species *Caipirasuchus montealtensis*, *Caipirasuchus paulistanus*, and *Caipirasuchus stenognathus*. The new material represents the holotype of a new species, *Caipirasuchus mineirus* n. sp., diferenciated from the previously ones due to the following traits: last two maxillary teeth located posterior to anterior edge of infraorbital fenestra, elongated lateroventral maxillo-jugal suture—about ½ the anteroposterior maxillar length—and contact between posterior crest of quadrate and posterior end of squamosal forming an almost 90° flaring roof of the squamosal, among others. *C. mineirus* was found in the same outcrop than *Campinasuchus* but stratigraphically the former occurs in the lower portion of the section with no unambiguous data supporting the coexistance of both taxa.

## Introduction

For about a century, since the description of the Patagonian Cretaceous crocodyliforms *Notosuchus terrestris* and *Cynodontosuchus rothi* (Woodward, 1896), the taxonomically diverse clade Notosuchia (Gasparini, 1971) remained hidden into the Late Cretaceous fossil record, with sporadic discoveries of now known to be closely related forms, coming from Brazil, Argentina, Malawi, and even China (Price, 1955; Bonaparte, 1991; Gomani, 1997; Wu, Sues & Sun, 1995; Wu & Sues, 1996). The history of notosuchians has been drastically modified since the last decades of the 20th century, when several discoveries and systematic studies improved the understanding of the group, not only taxonomically but also with paleobiogeographic and ecological implications (Buckley et al., 2000; Campos et al., 2001; Pol, 2003, 2005; Sereno et al., 2003; Turner, 2006; Zaher et al., 2006; Pol & Gasparini, 2007; De Andrade & Bertini, 2008a, 2008b, 2008c; Martinelli & Pais, 2008; Fiorelli & Calvo, 2008; Pinheiro et al., 2008; Novas et al., 2009; Sereno & Larsson, 2009; Carvalho et al., 2010; Kley et al., 2010; O’Connor et al., 2010; Turner & Sertich, 2010; Iori & Carvalho, 2009, 2011; Riff & Kellner, 2011; Iori & Garcia, 2012; Riff et al., 2012; Soto, Pol & Perea, 2011; Pol et al., 2014; Rabi & Sebők, 2015; Fiorelli et al., 2016). Based only on a few taxa until the 1990s, Notosuchia now includes more than 30 species (Pol & Leardi, 2015) and are among the most commonly recovered vertebrate remains from the Late Cretaceous rocks of the Bauru Group (Paraná Basin), in southeastern Brazil (Carvalho et al., 2010).

**Figure 1:**
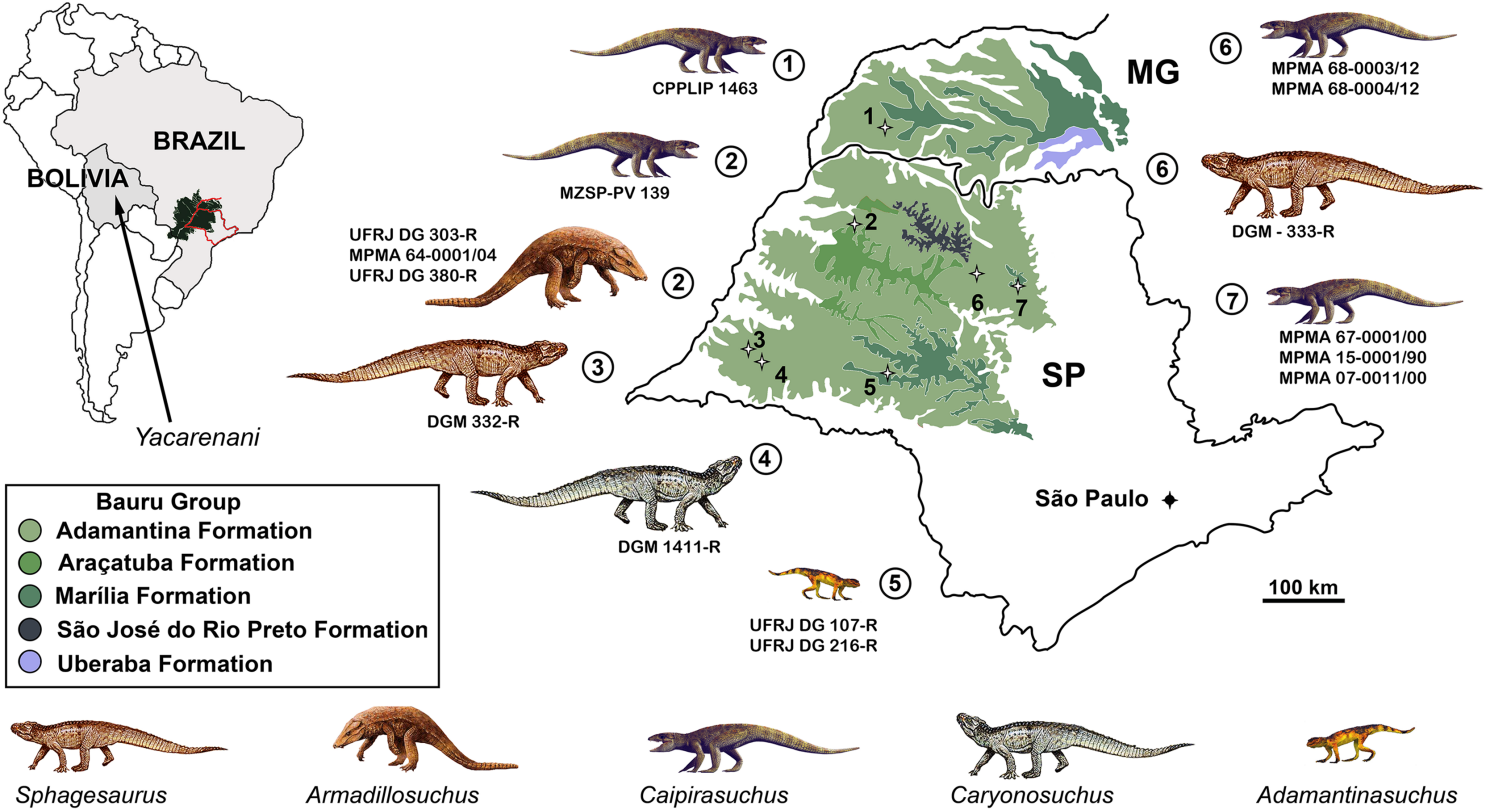
Distribution of South American sphagesaurids. The Brazilian sphagesaurid record in the states of Minas Gerais (MG) and São Paulo (SP), Brazil, as well as the sphagesaurid *Yacarerani* found in Bolivia. All come from Upper Cretaceous rocks.

Although notosuchians have a geographically broad distribution during the Cretaceous (Riff et al., 2012; Pol & Leardi, 2015), São Paulo and Minas Gerais states (southeastern Brazil) concentrate ∼20 species, including sphagesaurids (Price, 1950; Pol, 2003; Candeiro & Martinelli, 2006; Nobre & Carvalho, 2006; De Andrade & Bertini, 2008a; Marinho & Carvalho, 2009; Iori & Carvalho, 2011; Iori et al., 2013; Pol et al., 2014), baurusuchids (Price, 1955; Riff & Kellner, 2001, 2011; Carvalho, Campos & Nobre, 2005; Carvalho et al., 2011; Montefeltro, Larsson & Langer, 2011; Marinho et al., 2013; Godoy et al., 2014), and peirosaurids (Price, 1955; Carvalho, Ribeiro & Avilla, 2004; Carvalho, Vasconcellos & Tavares, 2007; Campos et al., 2011; although see Turner, 2006; Larsson & Sues, 2007; Zaher et al., 2006 for other hypotheses on the position of peirosaurids, outside Notosuchia). Notosuchia includes extremely disparate forms, such as terrestrial small- to medium-sized herbivorous species, terrestrial medium to large-sized active predators, with theropod-like adaptations in dentition and postcranium, and medium-sized semiaquatic to aquatic forms (Carvalho, Vasconcellos & Tavares, 2007; Carvalho et al., 2010; Riff & Kellner, 2011; Pol et al., 2014). Particularly, sphagesaurids have been proved to be the most diverse group of “advanced notosuchians” (sensu Pol et al., 2014), with at least eigth species distributed in the Brazilian state of São Paulo (Price, 1950; Nobre & Carvalho, 2006; Marinho & Carvalho, 2009; Kellner et al., 2011a; Iori & Carvalho, 2011; Iori et al., 2013; Pol, 2003; Pol et al., 2014), and one in central Bolivia (Novas et al., 2009) (Fig. 1).

**Figure 2:**
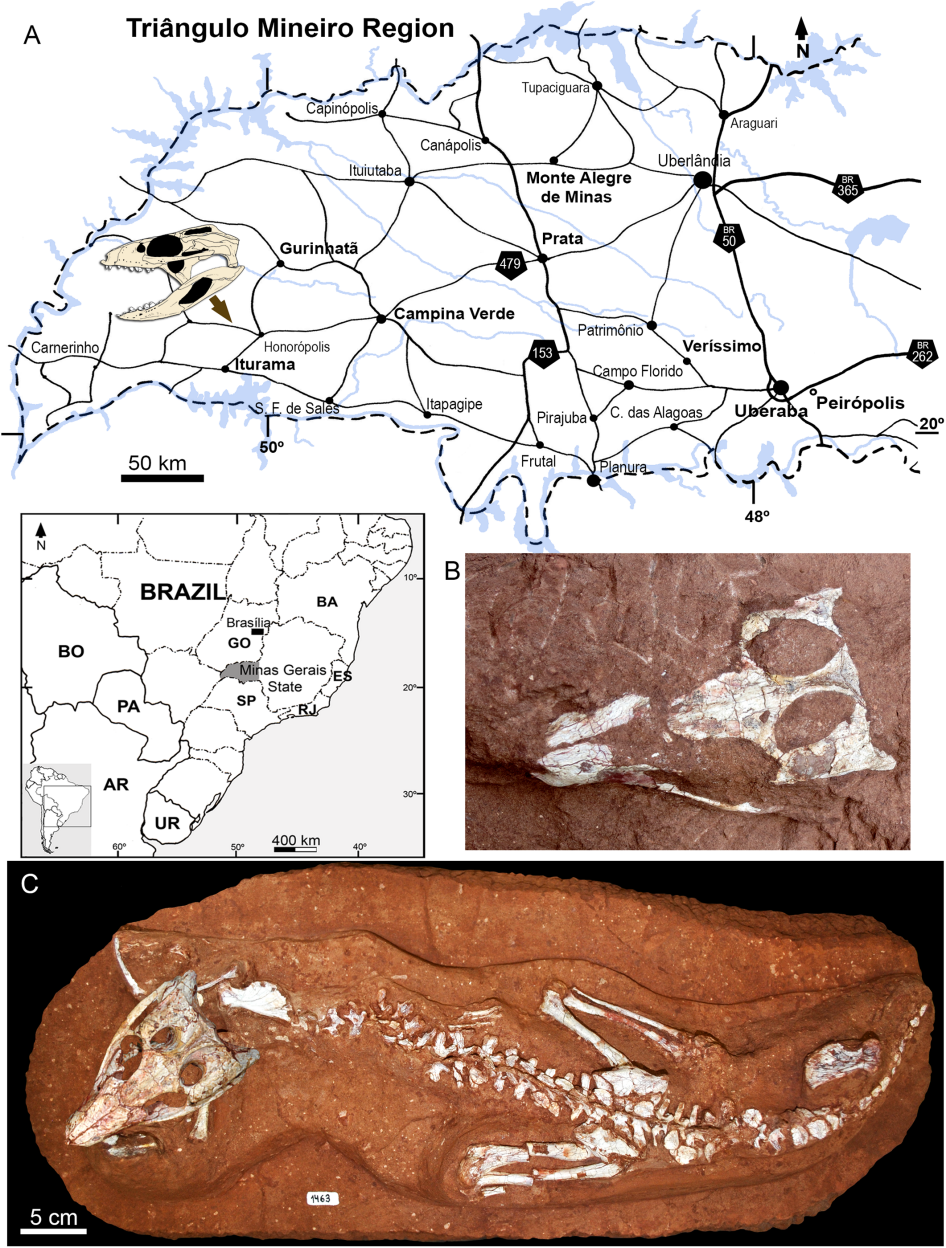
Map depicting the location of the “Fazenda Três Antas” site, in Campina Verde municipality, state of Minas Gerais, Brazil. (A) The arrow indicates where the holotype of *Caipirasuchus mineirus* was found. (B) Photograph of the skull in the field, in dorsal view. (C) Entire skeleton after preparation. Abbreviations: AR, Argentina; BA, Bahia state; BO, Bolivia; ES, Espirito Santo state; GO, Goias state; PA, Paraguay; UR, Uruguay; SP, São Paulo state; RJ, Rio de Janeiro state.

In 2009, staff of the Centro de Pesquisas Paleontológicas L. I. Price (CPPLIP, Universidade Federal do Triângulo Mineiro (UFTM), Uberaba, Minas Gerais) started prospecting for fossils in the region of Campina Verde (Minas Gerais, Brazil) (Fig. 2A). These field works have resulted in the discovery of a diverse Late Cretaceous continental fauna, distributed in at least three localities. The described taxa include the baurusuchid *Campinasuchus dinizi*, based on several specimens (Carvalho et al., 2011; Cotts et al., 2017), a partial crocodyliform egg (Marinho et al., 2012a), an isolated abelisaur theropod tooth (Marinho et al., 2012b), a femur of a noasaurid theropod (Martinelli et al., 2016), and hundreds of partial specimens of lepisosteiform fishes (Martinelli et al., 2012a, 2016; Martinelli & Teixeira, 2015). The 2014 field work, in the paleontological site Fazenda Três Antas (FTA), resulted in the discovered of an exquisitely preserved, partially articulated, 70 cm-long mesoeucrocodylian skeleton (CPPLIP 1463; Figs. 2B and 2C). This new specimen represents the first record of the sphagesaurid genus *Caipirasuchus* (Iori & Carvalho, 2011; Iori et al., 2013; Pol et al., 2014) outside the state of São Paulo. Moreover, CPPLIP 1463 exhibits a suite of features that diagnoses a new species. The description of the cranial and postcranial anatomy of the new species, taxonomical comments on the other species of the genus, and the inclusion of this taxon in a phylogenetic analysis, are presented below.

### Geological settings

**Figure 3:**
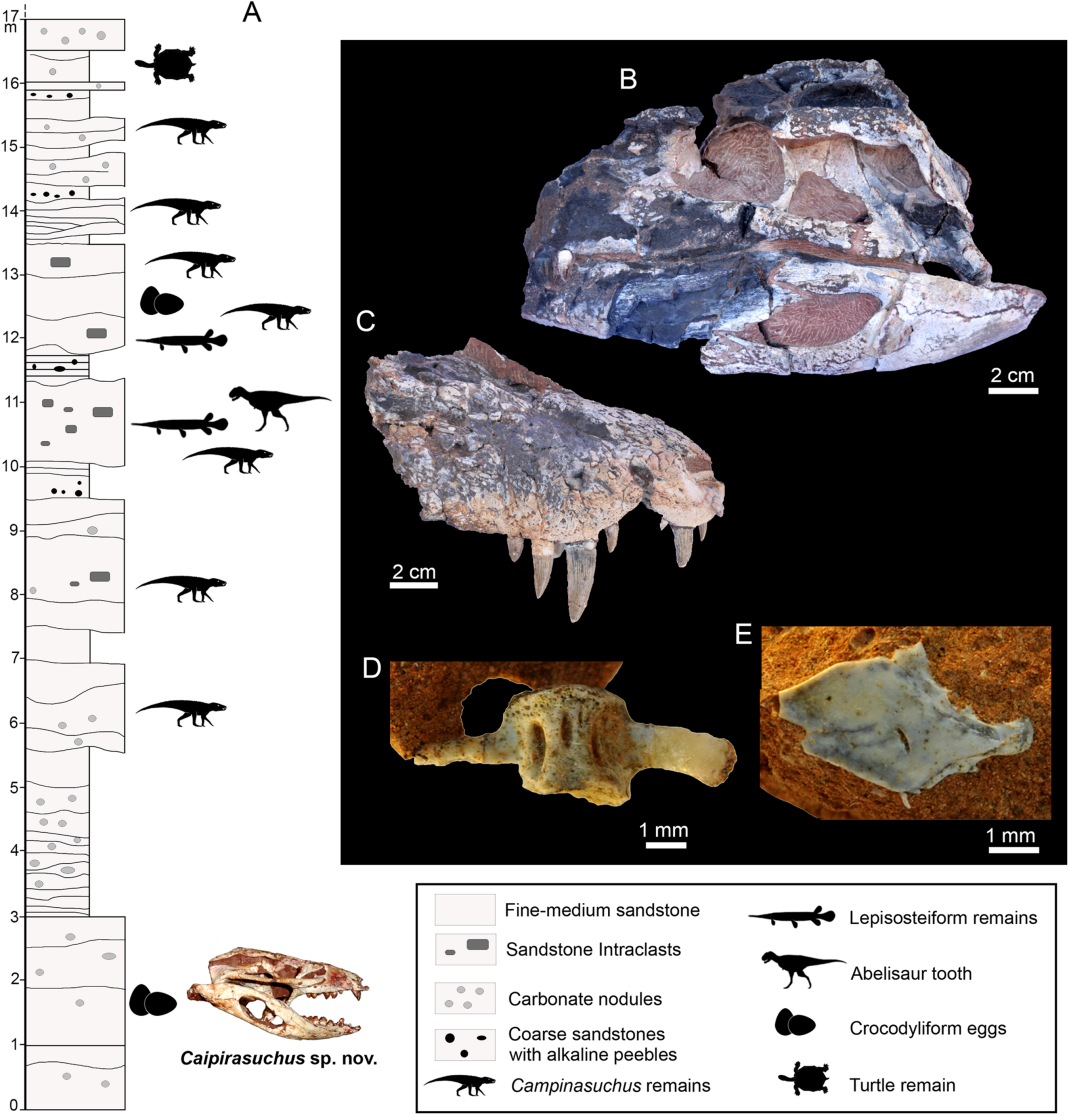
Stratigraphic log of the “Fazenda Três Antas” site, Municipality of Campina Verde, state of Minas Gerais, Brazil. (A) The skull refers the position of *C. mineirus* in the stratigraphic column. Some other fossils discovered at the same outcrop are: (B) Holotype CPPLIP 1235 of *Campinasuchus dinizi*. (C) Referred specimen CPPLIP 1236 of *C. dinizi*. (D) Vertebra CPPLIP 1247 of Lepisosteiformes indet. (E) Scale CPPLIP 1273 of Lepisosteiformes indet.

The FTA site in Campina Verde (Brazil) has several small fossiliferous outcrops, with a rich vertebrate fauna (Figs. 2 and 3). The exposed rocks in this region are referred to the Adamantina Formation (Bauru Basin, Upper Cretaceous), which is the most extensive outcropping unit of the Bauru Group (Soares et al., 1980; Fernandes & Coimbra, 1996; Batezelli & Ladeira, 2016; Menegazzo, Catuneanu & Chang, 2016). It is locally overlayed by the Echaporã Member of the Marília Formation (Batezelli & Ladeira, 2016; Menegazzo, Catuneanu & Chang, 2016) and it has been partially chronologically correlated to the Uberaba Formation, exposed in Uberaba and surrounding areas (e.g., in the municipality Veríssimo) (Dias-Brito et al., 2001; Menegazzo, Catuneanu & Chang, 2016). Some authors (Fernandes, 1998; Fernandes & Coimbra, 2000) have abandoned the use of the Adamantina Formation in order to divide it in several geographically smaller units, with distinctive facies, in the state of São Paulo. The Vale do Rio do Peixe, Presidente Prudente, São Jose do Rio Preto, and Araçatuba formations were the units proposed in substitution to part of the Adamantina Formation (Fernandes, 1998; Fernandes & Coimbra, 2000). Nevertheless, the use of the Adamantina Formation is still favoured over the alternative terminology (applied to the state of São Paulo state), especially in works regarding the outcrops in the Triângulo Mineiro region, in which Campina Verde is located (Paula e Silva, Kiang & Caetano-Chang, 2009; Batezelli, 2010; Batezelli & Ladeira, 2016; Menegazzo, Catuneanu & Chang, 2016; Pinheiro et al., 2018). Thus, we opted to follow these more recent geological contributions and favor use of Adamantina Formation in the Triângulo Mineiro region.

Outcrops of the FTA site occur in ravines formed by superficial erosion of the terrain. They are composed by fine- to coarse-grained red sandstones, intercalated with reddish siltstones and mudstones, and rare conglomerate levels. Calcrete surfaces also occur in several levels, as well as carbonate concretions (Fig. 3). These sediments were deposited in shallow braided fluvial systems, in which the fossils were buried mainly in alluvial plains in flood events, in a predominantly dry and hot climate (Goldberg & Garcia, 2000; Carvalho et al., 2011).

The age of the Adamantina Formation is still open to question. Dias-Brito et al. (2001) suggested a Turonian–Santonian age, based on micropaleontological and isotopic studies. A Campanian–Maastrichtian age was also proposed, based on ostracods (Gobbo-Rodrigues, Petri & Bertini, 1999) and vertebrates (Bertini et al., 1993; Santucci & Bertini, 2001; Martinelli & Teixeira, 2015; Salgado & Carvalho, 2008). Tamrat et al. (2002), based on magnetostratigraphic studies, suggested that the Uberaba Formation, which was arguably correlated with the Adamantina Formation, could not be older than Campanian. According to Goldberg & Garcia (1995), the Uberaba Formation has a rough lateral contact with the Adamantina Formation in the northwest region of the Uberaba municipality and most stratigraphic columns of the Bauru Basin placed Uberaba and Adamantina (Vale do Rio do Peixe Formation in Fernandes & Coimbra, 2000) as laterally correlated formations (Fernandes & Coimbra, 2000; Batezelli, Saad & Basilici, 2007). As a consequence, following the reasoning that Uberaba and Adamantina formations can be correlated, at least part of the Adamantina Formation can be considered Campanian. A recent study (Castro et al., 2018) provides high-precision U-Pb post-Turonian maximal age for an outcrop of the Adamantina Formation located in western São Paulo state, suggesting a late Coniacian–late Maastrichtian temporal constraint. However, the absolute age of the FTA’s outcrops is unknown and we consider them as belong to the upper part of the Late Cretaceous.

The specimen here described (CPPLIP 1463) comes from the lowermost level exposed at the FTA site, about four meters below the lowermost level bearing *Campinasuchus dinizi* and lepisosteiform remains (Fig. 3A). The layer that yielded CPPLIP 1463 is much less fossiliferous than the other layers of this locality. In addition to CPPLIP 1463, a few partial eggs were found in the same level, about one meters apart. They possibly correspond to crocodyliforms and will be described elsewhere.

## Materials and Methods

The specimen described here, as well as those used for comparisons, belong to public collections and were examined with the explicit permission of appropriate curators and/or collection managers. We followed all Brazilian regulations for fossil collection.

The specimen CPPLIP 1463 is housed at the CPPLIP of the UFTM, in Uberaba (Minas Gerais, Brazil). This material was discovered and excavated in July 2014. Fossil preparation was performed using needles and pneumatic tools. We compared the morphology of CPPLIP 1463 with other mesoeucrocodylian taxa, based on first hand examination and the literature, which are detailed along the text.

The electronic version of this article in portable document format will represent a published work according to the International Commission on Zoological Nomenclature (ICZN), and hence the new names contained in the electronic version are effectively published under that Code from the electronic edition alone. This published work and the nomenclatural acts it contains have been registered in ZooBank, the online registration system for the ICZN. The ZooBank Life Science Identifiers (LSIDs) can be resolved and the associated information viewed through any standard web browser by appending the LSID to the prefix http://zoobank.org/. The LSID for this publication is: urn:lsid:zoobank.org:pub:EF45EECD-02FD-433C-B747-21341A7CF7C2. The online version of this work is archived and available from the following digital repositories: PeerJ, PubMed Central, and CLOCKSS.

### Phylogenetic analysis

The specimen CPPLIP 1463 was included in the dataset of Fiorelli et al. (2016), which constitutes an updated version of that of Pol et al. (2012, 2014) and other sources (Clark, 1994; Ortega et al., 2000; Pol, 2003; Turner & Sertich, 2010; De Andrade et al., 2011; Leardi, Fiorelli & Gasparini, 2015a; Leardi et al., 2015b). The data matrix includes 113 terminals and 440 characters (File S1). The data scores for *Caipirasuchus mineirus* is also detailed in File S2.

The phylogenetic analysis was conducted under equally weighted parsimony, using the software TNT 1.5 (Goloboff, Farris & Nixon, 2008; Goloboff & Catalano, 2016). We maintained the same additive (=ordered) characters used by Fiorelli et al. (2016): Chapters 1, 3, 6, 10, 23, 37, 43, 44, 45, 49, 65, 67, 69, 71, 73, 77, 79, 86, 90, 91, 96, 97, 105, 116, 126, 140, 142, 143, 149, 167, 182, 187, 193, 197, 226, 228, 279, 339, 356, 357, 364, 368, and 401. The parsimony analysis was conducted by performing a heuristic search of Wagner trees with 10,000 random addition sequences, followed by Tree Bisection Reconnection (TBR), and saving 10 cladograms per round (Random seeds = 1). The resulting most parsimonious cladograms (MPCs) were subjected to a final round of TBR branch swapping. In addition, Bremer support values were calculated.

## Results

### Systematic paleontology

Archosauria Cope, 1869
Crocodyliformes Hay, 1930 (*sensu* Clark, in Benton & Clark, 1988)
Mesoeucrocodylia Whetstone & Whybrow, 1983
Sphagesauridae Kuhn, 1968 (*sensu* Marinho & Carvalho, 2007 and Pol et al., 2014)
*Caipirasuchus* Iori & Carvalho, 2011

**Type species.** *C. paulistanus* Iori & Carvalho, 2011

**Included species.** *C. montealtensis* De Andrade & Bertini, 2008a; *C. stenognathus* Pol et al., 2014; *C. mineirus* sp. nov.

**Remarks.** Pol et al. (2014) reviewed in detail the taxonomy of the genus *Caipirasuchus*, originally erected by Iori & Carvalho (2011), with additional taxonomic and phylogenetic inferences by Iori et al. (2013). The new species described here has all the generic features listed in Pol et al. (2014: 4) with the exception of a small diastema in the dentary between the fifth and sixth tooth. In the holotype of the new species the alveolus of the fifth dentary tooth is merged with the alveolar groove of the remaining teeth (6th–10th). Therefore, this character-state should no longer be considered as diagnostic for the genus. Taxonomic considerations of the four known species are provided in the Discussion section.

*Caipirasuchus mineirus* sp. nov. *urn:lsid:zoobank.org:act:8A54B326-3323-4EAC-9811-A8146AE110B5*

**Holotype.** CPPLIP 1463, an almost complete skeleton. The skull and lower jaws were removed from the rock matrix and the postcranial skeleton remains articulated on the rock in its original position (Figs. 2B and 2C).

**Etymology.** *Mineirus* refers to the state of Minas Gerais, southeastern Brazil, where the holotype was found, which has one of the most comprehensive Late Cretaceous continental fossil records in Brazil. In addition, it represents the first specimen and species of *Caipirasuchus* found outside the state of São Paulo.

**Locality and Horizon.** “FTA” site, Honorópolis District, Campina Verde County, Minas Gerais, Brazil. Adamantina Formation, Bauru Group, Bauru Basin, Upper Cretaceous.

**Diagnosis.** Small-sized (skull length 11.74 cm; see Table 1 for a complete list of measurements) sphagesaurid mesoeucrocodylian that differs from the other species of the genus by having the unique combination of characters (autapomorphies with an asterisk): two last maxillary teeth located posterior to the anterior edge of the suborbital fenestra*; last two maxillary teeth located posterior to the anterior rim of the orbit*; elongated ventrolateral maxillo-jugal suture (about half of the anteroposterior maxillary length); large and slender descending process of the lacrimal, passing ventrally to the antorbital fenestra; elongated suborbital fenestra (three times longer than wide); distal body of quadrate with parallel lateral and medial edges (in posterior view) and lateral and medial condyles equal in size, placed almost at the same height (the medial condyle is slightly ventral to the lateral one, but not as marked as in the remaining species of *Caipirasuchus*); posteromedial crest of the quadrates projected to the intercondylar groove instead of being projected to the medial condyle*; contact between the posterior crest of quadrate and the posterior end of squamosal forming an almost 90° flaring roof of the squamosal*; narrow and long choanal septum, which is almost as long as the length of suborbital fenestra; anteroposteriorly elongated mandibular fenestra (about three times longer than wide); reduced and only obliquely positioned suture between basisphenoid and quadrate; fourth dentary tooth the largest of the series; splenials occupy one fourth of the symphyseal length in dorsal view, exposition larger than in other *Caipirasuchus* species; D-shaped osteoderms without anteroposterior interlocking mechanism.

**Table 1:** Measurements of the skull, jaws, and some appendicular bones of the holotype CPPLIP 1463 of *C. mineirus*.

Skull	
Skull length (from the tip of the snout to the end of squamosal posterolateral process)	117.40
Basal skull length (from tip of snout to occipital condyle)	105.00
Rostrum length (from tip of the snout to anterior end of orbit)	41.00
Maximum skull width (at jugals)	60.00
Width secondary palate at third maxillary tooth	13.50
Maximum frontal width	27.90
Minimum frontal width	15.30
Anteroposterior orbital length	29.80
Temporal height (from quadrate condyle to skull roof)	39.80
Maximum length of suborbital fenestra	24.10
Maximum width of suborbital fenestra	7.80
Maximum length of supratemporal fossa	14.70
Maximum length of supratemporal fenestra	24.80
Length of choanal septum	21.30
Lower Jaw	
Mandibular length (from anterior tip to posterior end retroarticular process)	L123.70/R124.40
Maximum symphyseal length (in ventral view)	38.60
Dentary length (from anterior tip to posterior end of posterodorsal process)	L72.50/R76.20
Anteroposterior length of mandibular fenestra	35.80
Maximum height of mandibular ramus	22.80
Postcranium	
Humerus, proximodistal length	74.10
Humerus, lateromedial width of proximal end	18.90
Humerus, lateromedial width of distal end	12.00
Radius, proximodistal length	60.00
Radius, lateromedial width of proximal end	14.00
Radius, lateromedial width of distal end	9.00
Femur, proximodistal length	99.80^*^
Femur, lateromedial width of distal end	22.00
Tibia, proximodistal length	100.1
Fibula, proximodistal length	87.2

**Notes:**

They are in millimeters. R, refers to the right side and L refers to the left side.

* Indicates estimated measurement.

## Description

The holotype of *Caipirasuchus mineirus* (CPPLIP 1463) is comprised of a fairly complete, articulated skeleton (Fig. 2C). The skull and lower jaws are remarkably well-preserved, without any evidence of taphonomic deformation. It is perhaps the best-preserved skull of *Caipirasuchus* known so far, with sutures between bones being clearly visible. Unfortunately, the teeth’s crowns are partially eroded and several features are only partially preserved. In this regard, the holotype of *C. stenognathus* (Pol et al., 2014) remains with the best-preserved dentition among *Caipirasuchus* species.

The postcranial skeleton is fairly well-preserved, but some vertebral elements of the cervical region, ribs, shoulder, and forelimb bones are missing. The skeleton is embedded in a hard sandstone block; therefore, some features are difficult to access (Fig. 2C). It is almost articulated, in a “resting position,” with flexed hindlimbs, and only a few shifted bones. For example, the left scapula was transported to the middle portion of the tail.

### Skull

**Figure 4:**
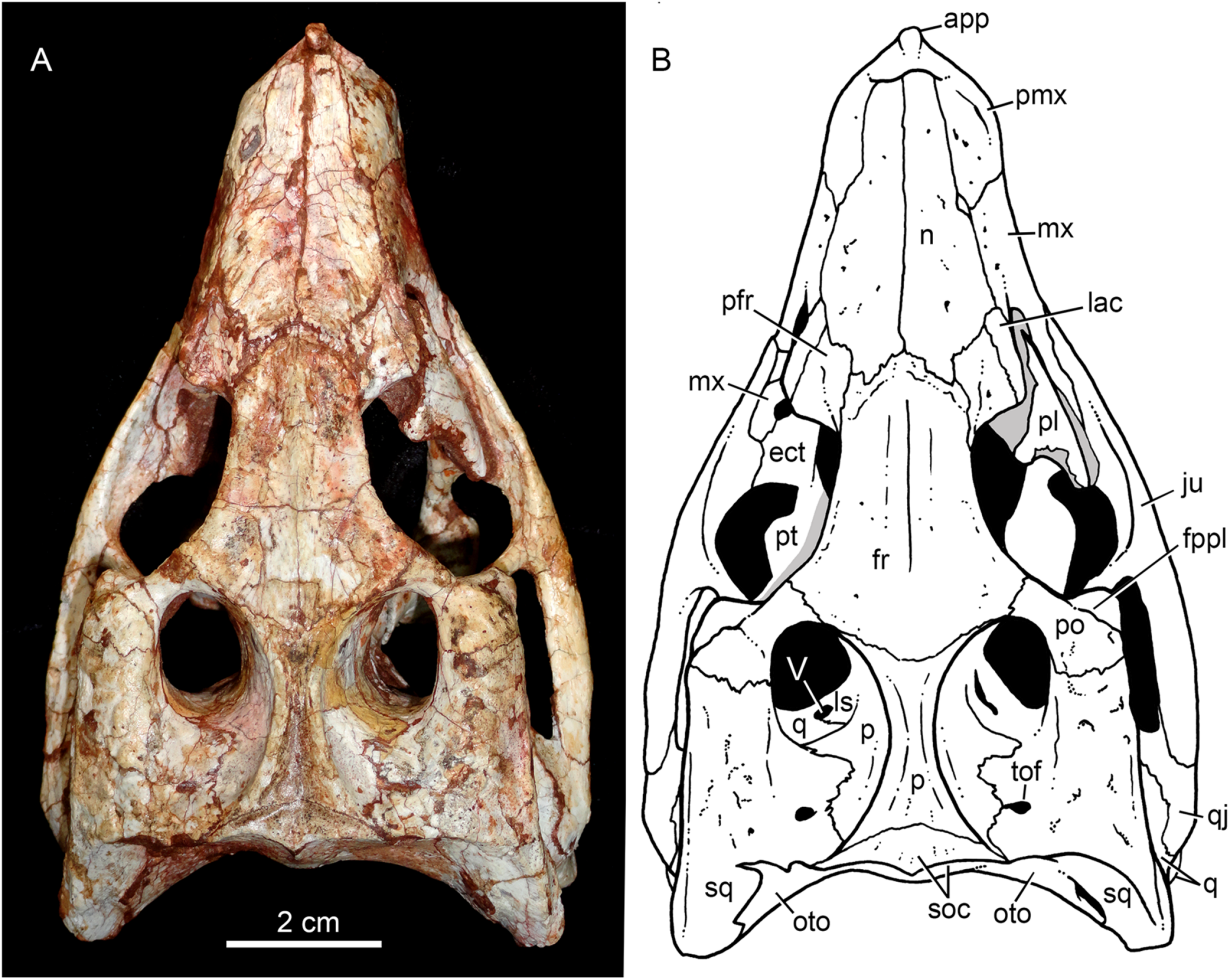
*Caipirasuchus mineirus*, CPPLIP 1463. Skull in dorsal view (A) with schematic drawing (B). Abbreviations: app, anterior process premaxilla; ect, ectopterygoid; fppl, facet for posterior palpebral; fr, frontal; ju, jugal; lac, lacrimal; ls, laterosphenoid; mx, maxilla; n, nasal; oto, otoccipital; p, parietal; pfr, prefrontal; pl, palpebral; pmx, premaxilla; po, postorbital; pt, pterygoid; q, quadrate; qj, quadrato-jugal; sq, squamosal; soc, supraoccipital; tof, temporo-orbital foramen; V, cranial nerve V.

**Figure 5:**
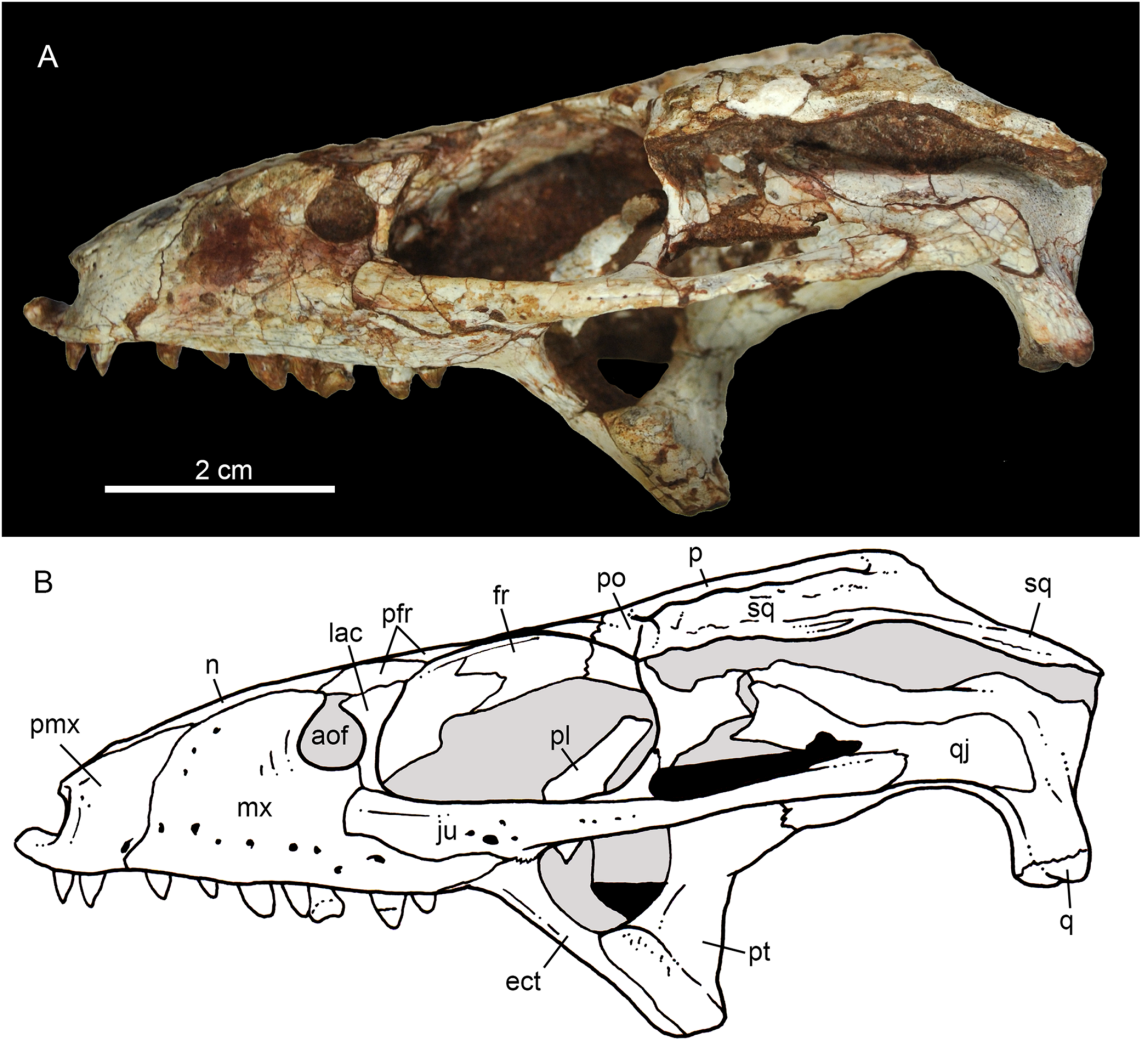
*Caipirasuchus mineirus*, CPPLIP 1463. Skull in left lateral view (A) with schematic drawing (B). Abbreviations: aof, antorbital fenestra; ect, ectopterygoid; fr, frontal; ju, jugal; lac, lacrimal; mx, maxilla; n, nasal; q, quadrate; qj, quadrato-jugal; p, parietal; pfr, prefrontal; pl, palpebral; pmx, premaxilla; po, postorbital; pt, pterygoid; sq, squamosal.

**Figure 6:**
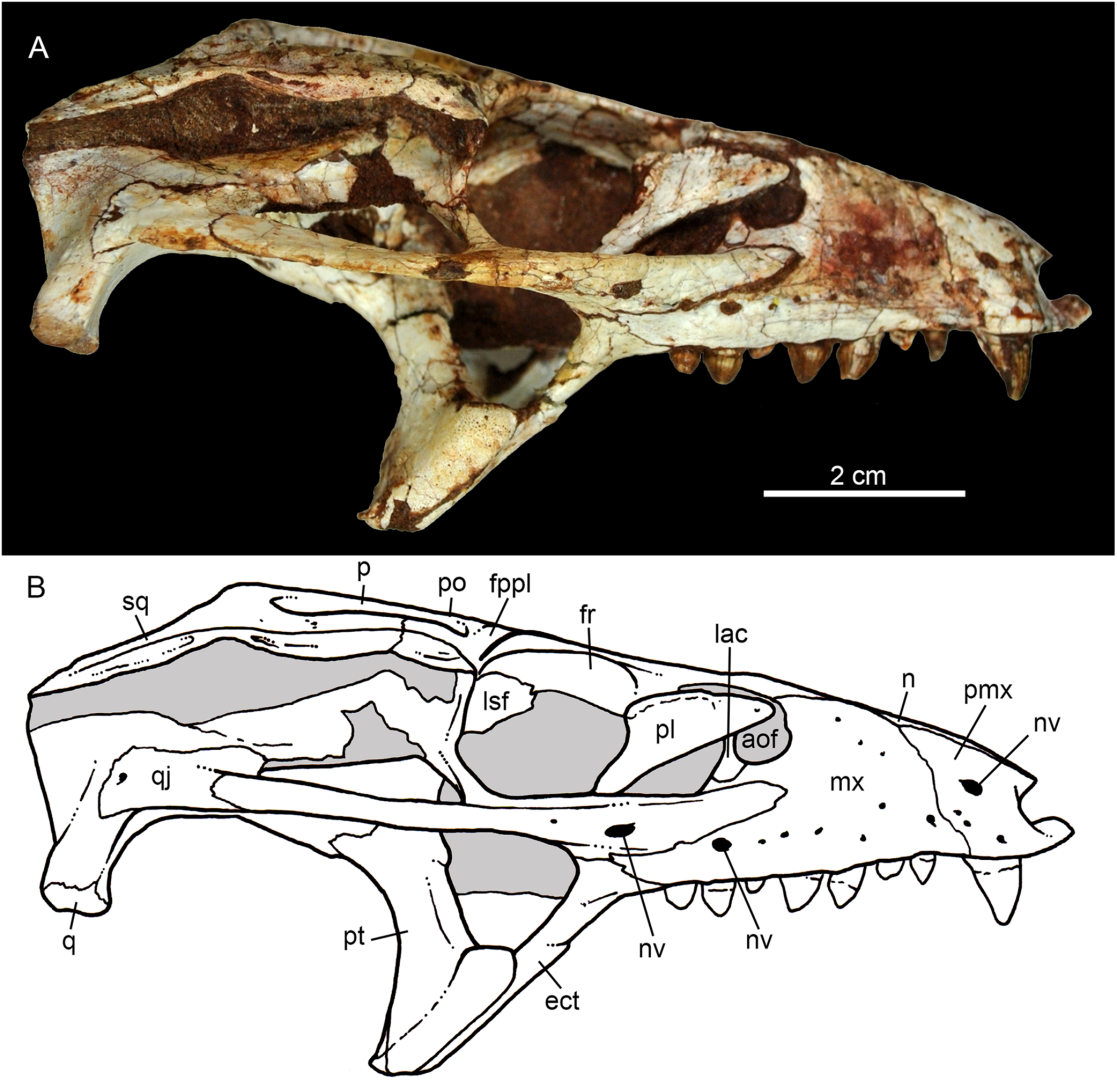
*Caipirasuchus mineirus*, CPPLIP 1463. Skull in right lateral view (A) with schematic drawing (B). Abbreviations: aof, antorbital fenestra; ect, ectopterygoid; fppl, facet for posterior palpebral; fr, frontal; lac, lacrimal; lsf, laterosphenoid; mx, maxilla; n, nasal; nv, neurovascular foramen; q, quadrate; qj, quadrato-jugal; p, parietal; pfr, prefrontal; pl, palpebral; pmx, premaxilla; po, postorbital; pt, pterygoid; sq, squamosal.

The skull has a short, cilindrical snout (1/3 of the skull length), large orbits and a subquadrangular skull roof with prominent posteriorly projected squamosal in dorsal and lateral views (Figs. 4A and 4B). The external nares are small and facing anteriorly. The large orbits are circular and laterally oriented. The morphology of both nares and orbits are typical of terrestrial animals, widely recognized among notosuchians (Gasparini, 1971; Carvalho, Campos & Nobre, 2005; Carvalho et al., 2011; Nobre & Carvalho, 2006; Pol, 2003; Pol et al., 2014). In lateral aspect, the skull tapers anteriorly, with a straight dorsal line (Figs. 5A, 5B, 6A and 6B). In this view, the pterygoid flanges are well developed, posteroventrally projected, as in most crocodyliforms (Iordansky, 1973; Clark, 1994). Both anterior palpebral bones are preserved. The right anterior palpebral is slightly shifted ventrolaterally from its original position, partially lying inside the right orbit and the antorbital fenestra. The left palpebral is disarticulated, within the orbital cavity (Figs. 5A and 5B). The palpebral bone has a triangular shape in dorsal aspect and is unsculptured (Figs. 6A and 6B). The posterolateral process of the palpebral is thin, with a concave medial border, and is slightly longer than the anterior process that rests on the posterodorsal border of the prefrontal–lacrimal. Posterior palpebrals are not preserved. A smooth facet on the anterolateral portion of the postorbital would be indicating the presence of an small posterior palpebral. A similar facet, althought more developed, was observed in *Caipirasuchus stenognathus* and due to its posterior extention it was considered an autapomorphy of the species (Pol et al., 2014). However, the facet for the posterior palpebral in *C. mineirus* is similar to that present in the holotype of *C. paulistanus* (MPMA 67-0001/00).

**Figure 7:**
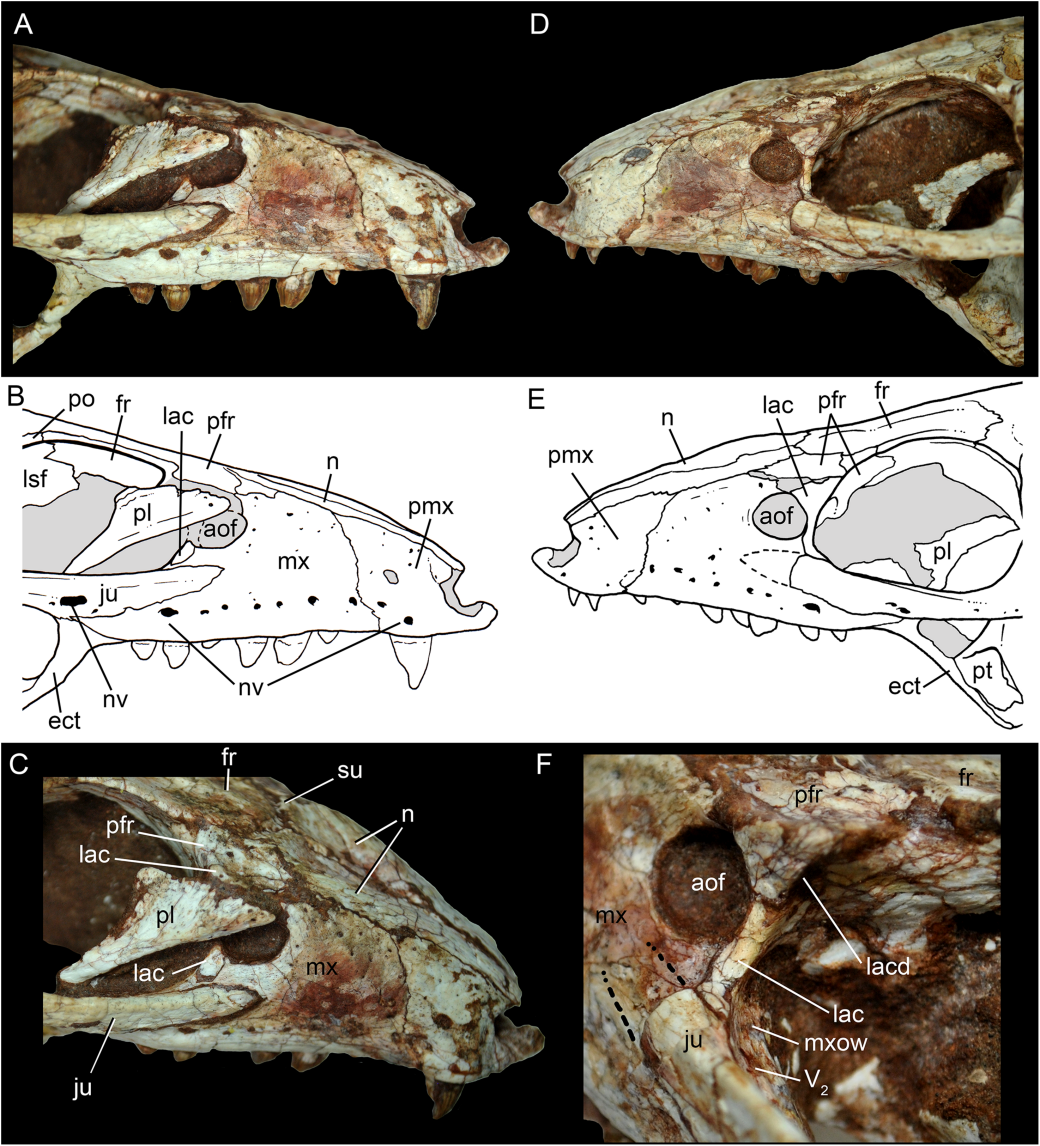
*Caipirasuchus mineirus*, CPPLIP 1463. Details of the snout in right lateral view (A) with schematic drawing (B), in right latero-anterodorsal view (C), in left lateral view (D) with schematic drawing (E), and of the inner orbital cavity in posterodorsal view (F). Abbreviations: aof, antorbital fenestra; ect, ectopterygoid; fr, frontal; ju, jugal; lac, lacrimal; lacd, lacrimal duct; lsf, laterosphenoid; mx, maxilla; mxow, maxillary orbital wall; n, nasal; nv, neurovascular foramen; pfr, prefrontal; pl, palpebral; pmx, premaxilla; po, postorbital; pt, pterygoid; V_2_, foramen maxillary branch of trigeminal nerve.

**Figure 8:**
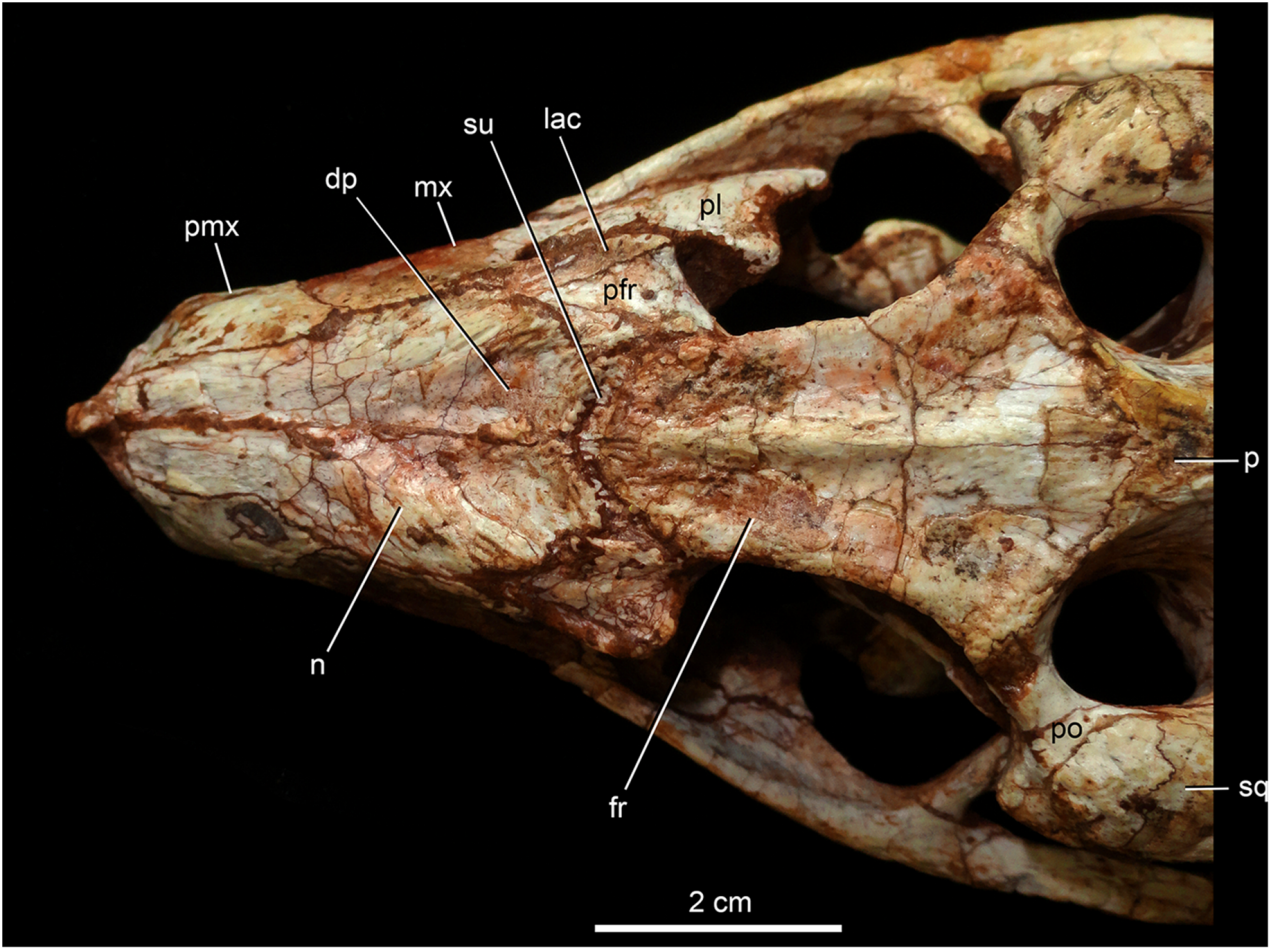
*Caipirasuchus mineirus*, CPPLIP 1463. Detail of the two-thirds anterior portion of the skull in dorsal view. Abbreviations: dp, depression; fr, frontal; lac, lacrimal; mx, maxilla; n, nasal; p, parietal; pfr, prefrontal; pl, palpebral; pmx, premaxilla; po, postorbital; su, sulcus; sq, squamosal.

The facial process of the premaxilla is well-developed as in *C. paulistanus*, *C. montealtensis*, and *C. stenognathus* and other “advanced notosuchians,” such as *N. terrestris* (Bonaparte, 1991; Barrios et al., 2018) and *Adamantinasuchus navae* (Nobre & Carvalho, 2006), but it is considerably larger in baurusuchids (e.g., *Gondwanasuchus scabrosus*; Marinho et al., 2013; *Aplestosuchus sordicus*; Godoy et al., 2014). Due to the size of the facial process, the nasal-premaxilla suture is anteroposteriorly elongated, extending itself as long as the nasal-maxilla suture (Figs. 5A, 5B and 7A–7C). The suture with the maxilla is interdigitated and starts at the alveolar level, posterior to the fourth caniniform tooth and runs posterodorsally. The posterodorsal portion of the premaxilla is broad and relatively short. The contact of the three bones (premaxilla, maxilla, and nasal) is at the level of the posterior border of the first maxillary tooth. The external surface of the premaxillary facial process is anteroposteriorly convex, due to the presence of the root of the largest maxillary tooth. There are many small nutritious foramina distributed on this unsculpted external surface. Between the premaxilla-maxilla suture, there is a conspicuous large nutritious foramen near the alveolar edge (Figs. 5A, 5B and 7A), similar to the condition of some other derived notosuchians (e.g., *N. terrestris*, *Comahuesuchus brachybuccalis*; Bonaparte, 1991; Martinelli, 2003; Barrios et al., 2018). In addition, only on the right side, there is a large foramen located more anterodorsally than the previous one, facing anteriorly. The perinarial depression is reduced, confined to the anteriormost portion of the bone, facing more anteriorly than laterally (Figs. 7A–7C). The anteromedial process of the premaxilla develops far anteriorly than the dorsal narial rim. This process contacts its counterpart at midline and projects anterodorsally, forming a procumbent projection. This configuration gives a triangular outline to the tip of the snout in dorsal/ventral view (Fig. 8). In other *Caipirasuchus* species, this structure is poorly preserved. There is no evidence of an ossified internarial bar, to contact these premaxillary processes and the nasals (Figs. 7A–7C), differing from the condition of baurusuchids and peirosaurids (Carvalho, Ribeiro & Avilla, 2004; Carvalho, Campos & Nobre, 2005; Carvalho, Vasconcellos & Tavares, 2007). The premaxilla forms the ventral, lateral, and a small portion of the dorsolateral edges of the external nares.

**Figure 9:**
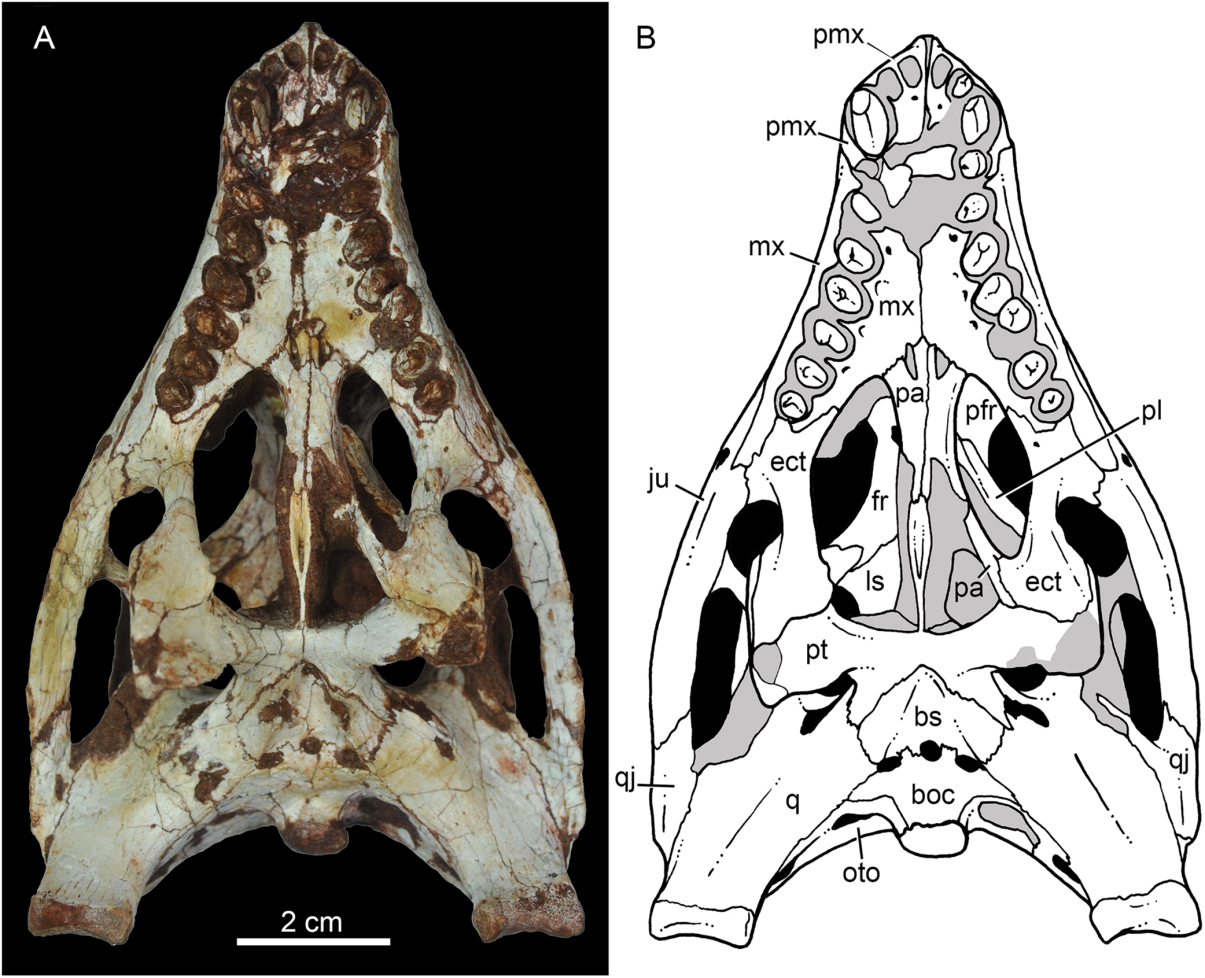
*Caipirasuchus mineirus*, CPPLIP 1463. Skull in ventral view (A) with schematic drawing (B). Abbreviations: bs, basisphenoid; boc, basioccipital; ect, ectopterygoid; fr, frontal; ju, jugal; ls, laterosphenoid; mx, maxilla; oto, otoccipital; pa, palatine; pfr, prefrontal; pl, palpebral; pmx, premaxilla; po, postorbital; pt, pterygoid; q, quadrate; qj, quadrate-jugal.

The palatal portion of each premaxilla bears four discrete alveoli, the third being the largest one, where the largest caniniform tooth is implanted (Fig. 9). The first and second alveoli are subrectangular, shorter mesiodistally than labiolingually, the third is large and suboval and the fourth is circular, larger than the first two alveoli. In ventral view, it is possible to observe how the premaxilla is laterally overlapped by the maxilla. Thus the fourth tooth, which in lateral view seems to be implanted into the maxilla, is in fact implanted into the premaxilla (Fig. 9), like in all other sphagesaurids known to date (Iori & Carvalho, 2011; Iori et al., 2013). The palatal contact between both premaxillae and premaxilla-maxilla is badly preserved, without clear information on the incisive foramina (Fig. 9).

The facial process of the maxilla is almost vertical, with a slight convexity near the alveolar edge, which is straight all along the bone. Its external surface is smoothly ornamented and, in addition to randomly distributed foramina, there is a line of seven distinctive foramina parallel and close to the alveolar edge (Figs. 5A, 5B and 7A–7C). The presence of two distinctive surfaces, one ornamented and dorsolaterally faced and the other vertical and unornamented (near the alveolar edge), observed in several “advanced notosuchians” (Pol, 2003; Pol et al., 2014), is poorly defined but present in *C. mineirus*. Most of the facial process of the maxilla is vertical and the ornamentation is so soft that the difference between both regions is poorly marked. The nasal-maxilla suture runs anteroposteriorly and forms a low ridge, with the nasal horizontally and the maxilla vertically positioned (Figs. 5A, 5B, 6A, 6B and 8). At the end of this suture, the maxilla and the nasal diverge where a thin process of the lacrimal wedges.

The maxillary facial process is the main structure to contribute to the edge of the circular antorbital fenestra, forming the dorsal, anterior, and ventral edges of this fenestra (Figs. 7A–7D). The posteroventral process of the facial lamina of the maxilla is extremely large in comparison to the other *Caipirasuchus* species. The lateral maxilla-jugal suture (more evident in the right side because in the left one the anteriormost tip of the jugal is broken) is about half of the anteroposterior length of the maxilla. It starts at the level of the anterior edge of the antorbital fenestra (or at the level of the distal edge of third maxillary tooth) and runs until the middle of the orbit. This suture is considerably shorter and more vertically oriented in other *Caipirasuchus* species. The jugal rests on this process, being only sutured in the posteroventral and dorsal portions. Conspicuously, the maxilla has a very small contribution to the orbital rim on the right side, whereas on the left side the thin descending process of the lacrimal contacts the jugal, excluding the maxilla of the orbital rim (Figs. 7A–7C). This small contribution in the right side has not the same development as in *C. stenognathus* (Pol et al., 2014) and it is here considered as an abnormality of CPPLIP 1463.

The maxilla contacts the lacrimal through a longitudinal suture, dorsally to the antorbital fenestra, and a vertical suture that starts at the posteroventral corner of the antorbital fenestra until it touches the jugal (on the left side). The maxilla-prefrontal contact is not present due to a thin anterodorsal process of lacrimal (Fig. 8).

The antorbital fenestra is well-preserved in both sides, although the right one is partially covered by the anterior palpebral. It is rounded, relatively large and with well-defined edges (see Discussion) (Figs. 5A, 5B, 6A, 6B and 7A–7C).

The maxilla has a large contribution to the internal orbital wall, forming its anterolateral floor. At the posteroventral region, the contacts with the jugal and ectopterygoid are observed. An oval foramen for the entrance of the maxillary branch of the trigeminal nerve (V_2_) is present in this region, facing posteriorly, that is, dorsally bordered by the maxilla and ventrally by the ectopterygoid (Fig. 7F). The maxillary orbital wall also extends dorsally to contact laterally the lacrimal and dorsally the descending process of the prefrontal, both contacted by means of a semicircular, elongated suture. A well-developed maxillary orbital wall was reported for *Sphagesaurus huenei* (Pol, 2003; RCL-100), being considered a synapomorphy of the clade composed by *Mariliasuchus amarali* plus Sphagesauridae (Pol et al., 2014).

The palatal contribution of the maxilla (Fig. 9) is large in comparison to *Caipirasuchus paulistanus* and *C. montealtensis* (Iori et al., 2013), because in these latter taxa the teeth are closer to the midline of the skull, narrowing the secondary palate. In *C. stenognathus*, the palatal contribution of the maxilla seems to be similar to CPPLIP 1463; acknowledging that in the holotype of *C. stenognathus* the skull is laterally deformed. The palatal maxillary process is strait, with sharp interalveolar processes that do not reach the lateral alveolar edge; therefore, the alveoli are connected to each other at the mesial–distal contact. In palatal view, the contact with the premaxilla is broken. The midline contact between both maxillae starts at the level of the first two teeth until the level of the distal edge of the fourth tooth; then, it contacts the palatine. At this point, there are two elongated maxillo-palatine fenestrae, bordered anteromedially by the maxilla and posterolaterally by the palatine (Fig. 9). The fenestrae are about three times longer than wide, positioned at the level of the fourth maxillary tooth. Maxillo-palatine fenestrae are present in *C. stenognathus* (Pol et al., 2014), and also occurs in *Mariliasuchus amarali* (De Andrade, Bertini & Pinheiro, 2006; Zaher et al., 2006), *Llanosuchus tamaensis* (Fiorelli et al., 2016) and *N. terrestris* (MACN-PV-RN 1038; De Andrade & Bertini, 2008b; Barrios et al., 2018), but they are absent in the other *Caipirasuchus* species (MPMA 67-0001/00; MPMA 15-0001/90; Iori et al., 2013).

The maxilla-palatine suture has two regions: a transversal and reduced medial one between the fenestrae, and another in which the palatine anterolateral process expands and contacts the maxilla by means of an oblique, interdigitated suture (Fig. 9). The anterior end of this oblique suture continues until the anteriormost level of the fourth maxillary alveolus. The right and left oblique sutures, although not touching one another at the midline due to the fenestrae, forms an open V-shaped contour of about 80°. This sutural condition is quite similar among *Caipirasuchus* species (Iori et al., 2013; Pol et al., 2014). The main difference is the lack of maxilla-palatine fenestrae in *C. paulistanus* and *C. montealtensis*, as aforementioned.

In ventral view, the maxilla contributes to the anterolateral border of the suborbital fenestra (Fig. 9). Also, the last two maxillary teeth are positioned posterior to the anterior border of the suborbital fenestra. The last maxillary alveolus is not completely closed by the maxilla, due to a small portion of the ectopterygoid that forms part of its posterolateral rim (Fig. 9).

The nasals form the dorsal roof of the snout, representing about one third of the skull’s length (Figs. 4A, 4B and 8). They form most of the dorsal edge of the external nares and are slightly concave on its anterior third. The lateral sutures are slightly asymmetrical. On the right side, the suture with the premaxilla diverges slightly posteriorly, and at the contact with the maxilla-premaxilla-nasal it turns slightly laterally; and then it runs until it contacts the prefrontal, where it obliquely turns medially to reach the frontal. On the left side, the suture with the premaxilla is more concave laterally and then more concave medially with the maxilla than in the right side. The contact with the fused frontals is an open V-shaped suture (Figs. 4A, 4B and 8). At this point, there is a deep groove along all the suture, delimited anteriorly and posteriorly by a ridge of bone formed by the nasal and frontal, respectively. This condition is also present in *C. paulistanus*, for which it was originally considered an autopomorphy (Pol et al., 2014). Conspicuously, there is a triangular depression with a low crest at the midpoint of the suture, on the posterior half of the nasals, that produces elevated and transversely concave lateral borders of the nasal (Fig. 8). This depression has shallow ornamentation consisting of shallow parallel grooves, especially near the suture with the prefrontal and frontals. A similar feature was described for *C. stenognathus*, as an autapomorphy (Pol et al., 2014). Although much less developed, we have observed a triangular depression in the nasals of *C. paulistanus* (MPMA 67-0001/00) and *C. montealtensis* (MPMA 15-0001/90; see also Iori et al., 2013).

The lacrimals are well-preserved on both sides of the skull, but the internal portion of the right side is partially obscured by the shifted anterior palpebral (Figs. 7A, 7B and 8). The dorsal plate is subrectangular, slightly anteromedially to posterolaterally inclined, with the posterior end tapering. It is four times longer than wide. The dorsal surface is slightly concave, with the posterolateral corner more elevated than the prefrontal, thus limiting laterally the surface to accommodate the anterior process of the anterior palpebral (Fig. 8). In lateral view, the descending process of the lacrimal narrows abruptly to form a thin, vertical bar that limits the posterior edge of the antorbital fenestra. At the contact with the jugal, it turns posteriorly to wedge between the anterior process of the jugal and the maxillary orbital wall. In the orbital cavity, the lacrimal descending process contacts the descending processes of the prefrontal and the maxilla (Figs. 7A–7F). Just lateral to this three-sutural point, there is a small lacrimal foramen, facing posteriorly. The lateral descending process and the orbital wall of the lacrimal forms a sharp crest that constitutes the anteriormost border of the orbit.

As described before, in the right side of the skull, the descending process of the lacrimal does not reach the jugal (Figs. 7A and 7B). We consider this as an abnormality, based on the left side of the skull, in which the lacrimal reaches the jugal, as in *C. paulistanus* and *C. montealtensis*. Additionally, the long and slender descending process of the lacrimal is considered autapomorphic for *C. mineirus*. In *C. paulistanus*, *C. montealtensis*, and *C. stenognathus* it is short and stout, contacting the jugal only in the two former species due to the presence of a dorsomedial process on the jugal (Iori et al., 2013; Pol et al., 2014).

The dorsal plate of the prefrontal is diamond-shaped, being more anteroposteriorly elongated than transversely wide (Fig. 8). The suture with the lacrimal is slightly oblique (anteromedially to posterolaterally oriented), parallel to the medial suture of the nasal and frontal, whereas the anterior suture with the nasal is anterolaterally to posteromedially oriented. The dorsal surface of the lacrimal is slightly concave and smooth to accommodate the anterior process of the palpebral. The shape of this bone is quite different from the condition of *C. paulistanus* and *C. stenognathus* in which the dorsal exposure is more triangular-shaped, being in these latter species more anteroposteriorly short and lateromedially broad. Also, in *C. mineirus* the prefrontal contacts the frontal by means of a straight suture, whereas in *C. stenognathus* the prefrontal-frontal suture is L-shaped. This difference makes the prefrontal more laterally positioned (and consequently more anteriorly placed with respect to the orbit) in *C. mineirus* than in *C. stenognathus*.

The descending process of the prefrontal is a large, slightly concave lamina that faces posteroventrally and forms the anterodorsal roof of the orbital cavity (Figs. 5A, 5B and 7A–7F). In CPPLIP 1463, it is sutured with the descending processes of both lacrimal and the maxilla. The prefrontal descending process has a posteromedial and slightly dorsal triangular projection that extends posteriorly below the frontal (only observed in the left orbital cavity), and reaches the level of the midpoint of the orbit.

The frontals are fused and are anteroposteriorly as long as the nasals (Figs. 4A, 4B and 8). There is a medial crest along the entire bone, that becomes lower at the posterior portion. The crest is sharper at the middle portion and widens anteriorly (Fig. 8). This condition resembles that of *C. paulistanus* (Iori & Carvalho, 2011), whereas in *C. montealtensis* and *C. stenognathus* the crest is not developed on the anterior fourth of the bone. Between this crest and the orbital rim, each side of the frontal is gently concave. The ornamentation is smooth, with several very small foramina and some shallow grooves. The frontal largely contributes to the dorsal rim of the orbit. This edge is sharp and concave, being this concavity more marked than in *C. paulistanus* and *C. montealtensis*, similar to *C. stenognathus*. The posterior contact with the parietal and postorbital is oblique (anterolateral to posteromedial) (Fig. 8). At the anterior edge of the supratemporal fossa where these bones meet, they form a sharp rim, with the frontal horizontally and the parietal and postorbital vertically positioned. Consequently, the contribution of the frontal to the supratemporal fossa is minimal, and the anterior vertical wall of the supratemporal fenestra is constituted by the parietal and postorbital where they meet through an interdigitated irregular suture.

The postorbital contributes to the margins of the supratemporal and infratemporal fenestrae and to the orbital rim (Figs. 4A, 4B, 5A, 5B, 6A and 6B). The bone widens at the anteromedial contact with the frontal and at the posterior contact with parietal, while it is more constricted where it forms the anterolateral edge of the supratempral fenestra. This pattern results in a more ventral position of the constricted midportion of the postorbital when compared to both anteromedial and posterior edges of the bone (Fig. 8). The posterolateral body of the postorbital has two main components: a dorsal portion, slightly ornamented with furrows, that contacts the parietal by means of a transversal interdigitated suture, positioned slightly anteriorly to the midpoint of the supratemporal fenestra; and a smooth triangular surface, that points anterolaterally, placed in a more ventral position than the dorsal portion. Both structures delimit an oblique groove that accommodates the posterior process of the posterior palpebral. This condition is also observed in *C. stenognathus* (Pol et al., 2014). The descending process of the postorbital has an anterior lamina that forms part of the posterolateral orbital cavity laterally with a very sharp rim (Figs. 5A, 5B, 6A and 6B). The postorbital widens ventrally to contact the jugal and expands posteriorly to contact the quadratojugal, forming most of the dorsal edge of the suborbital fenestra. The posterolateral descending process of the postorbital is laminar and concave, facing posterolaterally. At this region, the contact with the quadratojugal and parietal is obscured by cracks in both sides of the skull.

As the frontals, the parietals are fused at midline (Figs. 4A and 4B). The parietal has a dorsal plate, bordered by rounded, laterally concave crests that limit the supratemporal fossa. Medially to these crests, the parietals are transversely concave on the posterior half and flat to slightly concave on the anterior half. The transversal constriction of the parietal crests is more accentuated at the level of the posterolateral corner of the supratemporal fenestrae. The ornamentation at this portion is shallow, with some longitudinal furrows mainly on its anterior half. Posteriorly, the parietal contacts the supraoccipital by means of a transversely long and posteriorly concave suture (Figs. 4A and 4B). The lateral-most point of this suture reaches the level of the middle of the supratemporal fenestra. The descending process of the parietal is vertical in its anterior two thirds, at the medial and anteromedial edges of the supratemporal fenestra. The posteromedial edge of the supratemporal fenestra has a vertical component of the parietal, as well as a subtly concave subhorizontal component that forms the posteromedial floor of the supratemporal fossa. The lateral contact of the parietal with the squamosal is by an irregular W-shaped suture, where the squamosal partially rests on the parietal. Inside the supratemporal fenestra, the anterior half of the descending process of the parietal contacts the laterosphenoid through a horizontal suture.

**Figure 10:**
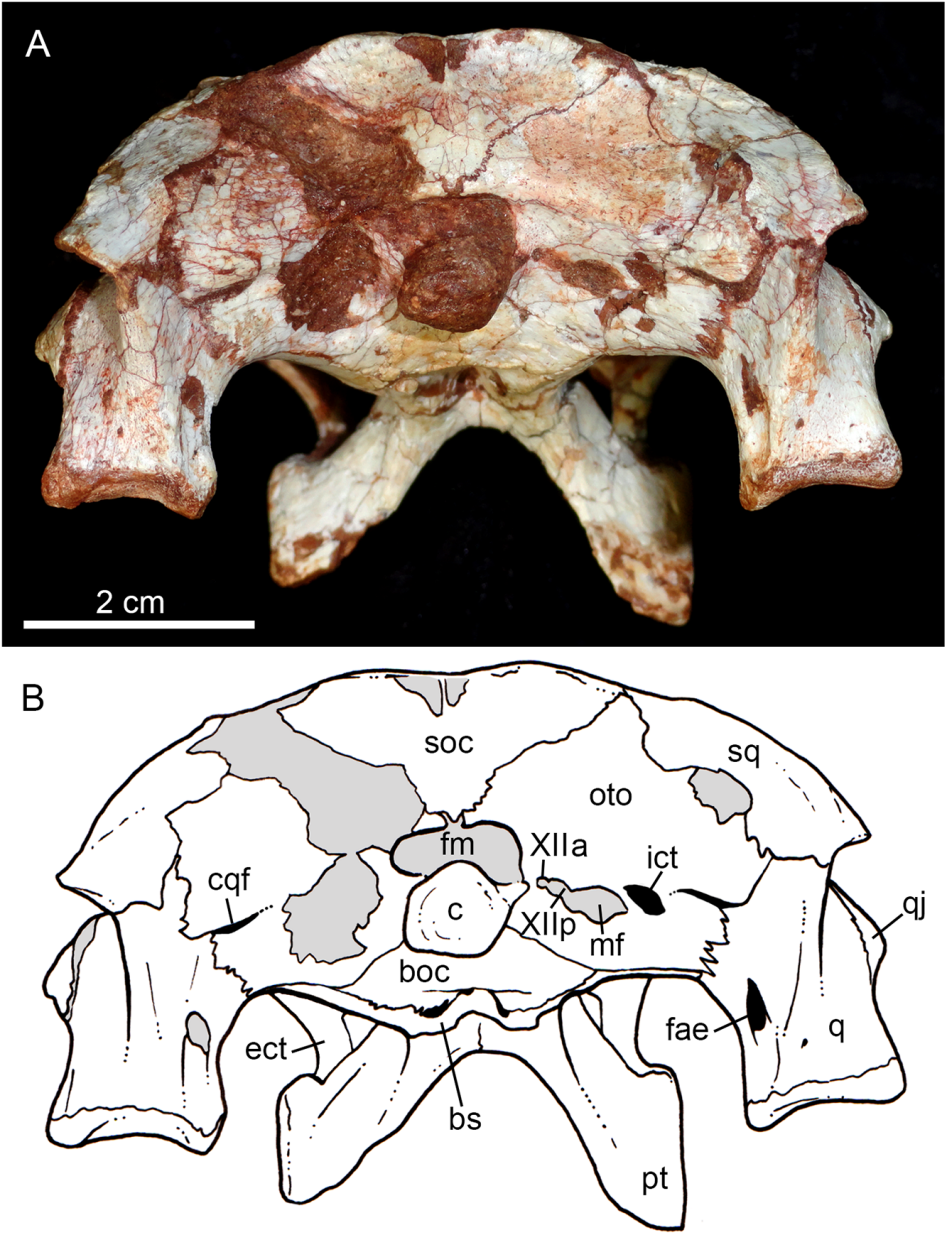
*Caipirasuchus mineirus*, CPPLIP 1463. Skull in occipital view (A) with schematic drawing (B). Abbreviations: bs, basisphenoid; boc, basioccipital; c, condyle; cqf, cranioquadrate foramen; ect, ectopterygoid; fm, foramen magnum; fae, foramen aërum; fpt, pterygoid; icf, internal carotid foramen; mf, metotic foramen; oto, otoccipital; pt, pterygoid; q, quadrate; qj, quadrate-jugal; soc, supraoccipital; sq, squamosal; XIIa, foramen for the anterior ramus of the hypoglossal cranial nerve; XIIp, foramen for the posterior ramus of the hypoglossal cranial nerve.

The squamosal is the largest bone of the cranial roof and is heavily ornamented (Figs. 4A and 4B). Both squamosals are preserved and no deformation is observed. The posteromedial process is square-shaped and slightly concave, and descends to the supratemporal fenestra to contact the anterodorsal process of the quadrate. On this posteromedial process, only the posterior third is ornamented, indicating the end of the supratemporal fossa. There is no conspicuous step on this portion of the skull, separating the supratemporal fossa from the skull roof. The oval temporo-orbital foramen is placed at the center of the posteromedial process and faces medially. The anterior process of the squamosal is robust, gently transversely convex and contacts the postorbital through a transversal, irregularly interdigitated suture. This suture is positioned anterior to the middle of the supratemporal fenestra, similar to *C. paulistanus*. In *C. stenognathus* this suture is far posteriorly placed, located at the posterior end of the supratemporal fenestra. At the anterior process of the squamosal, the ornamentation is more apparent. It consists of anastomosed grooves, mainly on the lateral two thirds of the bone, facing dorsolaterally. The medial surface is smooth, forming the lateral edge of the supratemporal fenestra. The posterior process of the squamosal is as large as the anterior process, slightly tapering posteriorly, and posteroventrally projected (Figs. 4A and 4B). There are two distinctive surfaces on the posterior process of the squamosal: a smooth surface facing posterodorsally, that represents the two thirds medial of the process and corresponds to the posterior profile of the skull; and a lateral surface gently ornamental, facing dorsally. In dorsal view, the squamosal posterior process forms as prominent projection that reaches the level of the posterior border of the quadrate. In lateral view, a sigmoid continuous process formed by the postorbital and the squamosal forms the lateral edge of the skull, with two waves at the level of the skull table and then posteroventrally projected until its end at the level of the quadrate. In lateral view, the anterior dorsal concavity of this postorbital-squamosal lateral edge is at the level of the supratemporal fenestrae, whereas the second concavity is at the level of the otic recess area. From the postorbital bar up to the end of the posterior process of the squamosal, the lateral border of the skull roof forms a continuous cavity, partially hidden in lateral view, where the morphology of the otic recess area is not accessed due to a hard matrix inside of it (Figs. 5A, 5B, 6A and 6B). In posterior view, the medial border of the posterior squamosal process reaches the prominent posterior crest of the quadrate and both forms a 90° angle, with a laterally flaring squamosal (Figs. 10A and 10B), not observed in other “advanced notosuchians.” This medial edge also contacts the dorsal projection of the otoccipital.

The jugal is a slender and long bone, well-preserved in both sides (Figs. 5A, 5B, 7A–7E, 9A and 9B). Its anteroposterior length represents more than half of the skull’s length. The suborbital portion of the jugal is longer than the posterior process (Figs. 5A and 5B). The jugal’s suborbital process tapers anteriorly as a sharp projection over the maxilla, that reaches the level of the distal edge of the third maxillary tooth. As described before, the contact with the lacrimal is reduced, only observed in the left side of the skull (Figs. 7D and 7E). The suborbital process is dorsally bounded by a sharp edge which constitutes the orbital rim. Below this crest, the body of the suborbital process expands until its posteromedial contact with the ectopterygoid. At this point, the jugal reaches its maximum depth. The anterior half of the suborbital process faces laterally, whereas it gradually turns downward and medially, facing ventrolaterally. At this point, a sigmoid anteroposterior suture with the maxilla develops until the midpoint of the orbit. Just above the three-sutural point (jugal, maxilla, and ectopterygoid) there is a large foramen facing anteriorly (Figs. 7A and 7B) that is also present in the other *Caipirasuchus* species. The specimen lacks the typical curved dorsal edge of the jugal, as other *Caipirasuchus* species. The curved dorsal edge of the jugal is seen in *Sphagesaurus huenei* (Pol, 2003), *Adamantinasuchus navae* (Nobre & Carvalho, 2006), and *Yacarerani boliviensis* (Novas et al., 2009). In addition to this large foramen, there is a very small foramen along the external surface of the jugal. This portion of the jugal is unornamented, as in other *Caipirasuchus* species, and differing from several notosuchians with ornamented jugals (e.g., *Armadillosuchus arrudai*, Marinho & Carvalho, 2009; *Sphagesaurus huenei*, Pol, 2003; *Baurusuchus* spp., e.g., Carvalho, Campos & Nobre, 2005).

The sharp jugal crest that characterizes the orbital rim becomes a rounded edge, facing dorsolaterally, up to the midlength of orbit. At this point, the body of the jugal becomes a transversely large and dorsoventrally thin lamina that runs posteriorly reaching the quadratojugal. This shape produces a subhorizontal platform, facing dorsolaterally that starts in front of the base of the medial suborbital bar (Figs. 5A, 5B, 6A and 6B). The posterior process projects slightly dorsally, and extends over the quadratojugal to form a U-shaped suture. The posteriormost tip of this suture is posteriorly positioned to the posterior rim of the infratemporal fenestra. The medial suborbital process of the jugal is circular in cross-section and posteromedially projected. It has an expanded base on the dorsomedial edge of the body of the jugal body. The connection between this process and the body of the jugal is gradual, without a marked step or groove. The suture between the postorbital and jugal is oblique (anterolateral to posteromedial), starting on the anterior edge of the suborbital process, at middle height of the fenestra, up to the posterior edge, near its dorsal-most tip.

The quadratojugal constitutes the posterodorsal edge of the suborbital fenestra by means of a laminar anteromedial process, that is dorsally sutured with the quadrate (Figs. 5A, 5B, 6A and 6B). The lateral exposure of the quadratojugal is relatively large, anteroposteriorly concave and without ornamentation. A large lateral exposure is not observed in *C. paulistanus*, being more similar to the condition of *C. montealtensis* and, apparently, *C. stenognathus* (in this latter taxon the quadratojugal is shifted from its original position; Pol et al., 2014). The thicker portion of the bone is in its ventral edge, at the midway between the sutures with the jugal and the quadrate.

**Figure 11:**
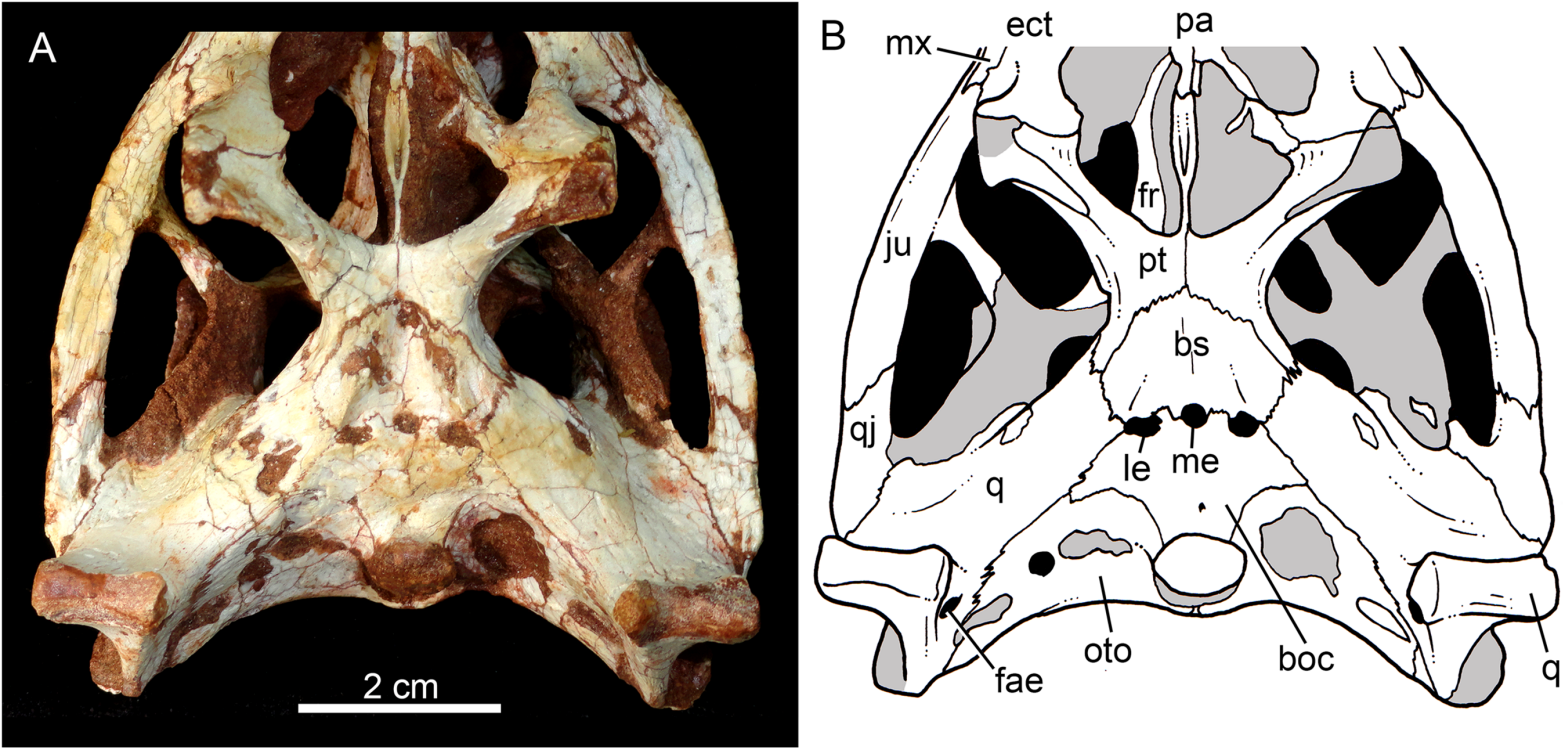
*Caipirasuchus mineirus*, CPPLIP 1463. Details of the primary palate and basicranium in ventral view (A) with schematic drawing (B). Abbreviations: bs, basisphenoid; boc, basioccipital; c, condyle; ect, ectopterygoid; fae, foramen aërum; fr, frontal; ju, jugal; le, lateral Eustaquian foramen; me, median Eustaquian foramen; mx, maxilla; oto, otoccipital; pt, pterygoid; pa, palatine; pt, pterygoid; q, quadrate; qj, quadrate-jugal.

**Figure 12:**
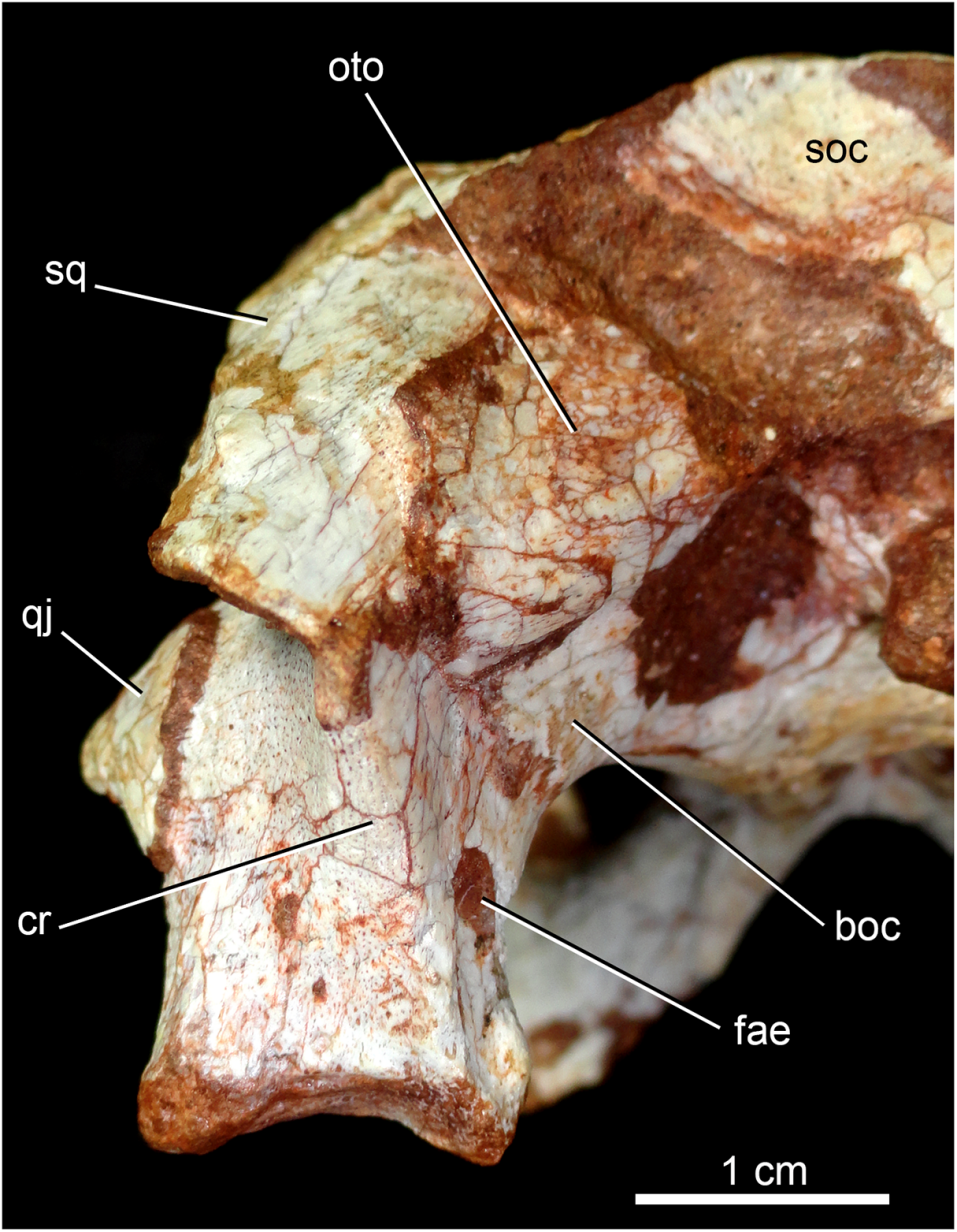
*Caipirasuchus mineirus*, CPPLIP 1463. Detail of the left posterolateral portion of the skull in occipital view. Abbreviations: boc, basioccipital; cr, crest; fae, foramen aërum; oto, otoccipital; q, quadrate; qj, quadrate-jugal; soc, supraoccipital; sq, squamosal.

The quadrates are well-preserved, with both articular regions and without any kind of deformation (Figs. 5A, 5B, 6A, 6B, 9A, 9B, 11A, 11B and 12). The distal body of the quadrate is ventrally projected, with its lowest point slightly above the alveolar level, if the maxillary alveolar edge is set horizontally (Figs. 5A, 5B, 6A and 6B). Accordingly to Pol et al. (2014), the articular region of the quadrate is positioned below the maxillary tooth row in *C. stenognathus*. Based on their Figures 10 and 11 (Pol et al., 2014: 11–12) and if the alveolar edge is positioned horizontally, the quadrate condyles are almost at the same level, very similarly to the condition of *C. mineirus*, which does not have any evidence of deformation. In posterior view, the distal body of the quadrate seems to be more laterally projected in *C. stenognathus* and *C. montealtensis* (Iori & Carvalho, 2018) than in *C. mineirus*; however, it could be result of the dorsoventral flattening of the skull of the former taxa. The medial condyle is positioned slightly ventral to the lateral one (Fig. 12), and it is anteroposteriorly smaller. In *C. paulistanus* the ventral development of the medial condyle is conspicuous, much more developed than in the other species (Iori & Carvalho, 2018), including *C. mineirus*. In ventral view, the quadrate articular surface is almost rectangular, about three times lateromedially broader than anteroposteriorly long, with a shallow intercondylar groove (Figs. 11A and 11B). This groove is considerably deeper in the other *Caipirasuchus* species (Iori & Carvalho, 2018). The quadrates bears a robust crest that starts at the center of the posteromedial edge of its body and projects posterolaterally (Fig. 12). In posterior view, the crest is wide at its base, forming a triangular depression above the intercondylar groove, and tapers dorsally until its contact with the squamosal (Fig. 12). In the other *Caipirasuchus* species, the posteromedial crest has a similar width along its extension, and ventrally extends to the medial condyle, a condition not seen in *C. mineirus*. The foramen aërum is placed on the quadrate body, anteromedially to the base of the posterolateral crest (Fig. 12). The foramen is relatively large, being of relatively greater dimensions than in *C. stenognathus*, and it is clearly seen in posterior view but mostly facing medially, as in most notosuchians (Pol et al., 2014). The dorsomedially projection of the quadrate contacts the otoccipital by means of a long and interdigitated suture, until it contacts posteromedially the basioccipital and anteromedially both basisphenoid and pterygoid (Figs. 11A and 11B).

The supraoccipital is well-preserved (Figs. 4A, 4B, 10A and 10B). This bone is only partially preserved in other *Caipirasuchus* species (Iori et al., 2013; Pol et al., 2014). It has two main components, the dorsal and occipital portions. The dorsal plate has a good contribution to the dorsal skull roof, with a subtriangular shape, and is more than three times broader transversely than anteroposteriorly (Figs. 4A and 4B). In other notosuchians, such as *N. terrestris*, the dorsal plate of the supraoccipital has a more equilateral triangular shape. The dorsal plate has a semicircular suture with the parietal and the contact with the squamosal is reduced in dorsal view, limited to the dorsolateral end of the bone. This suture is not interdigitated as in other bones. The dorsal plate is slightly concave, with two shallow grooves displayed, parallel to the suture with the parietal. The posterodorsal edge of the supraoccipital has a median, acute process and, laterally to it, the edges are gently concave. The median process has a faint crest that descends until the midheight of the occipital plate (Figs. 10A and 10B). The occipital portion of the supraoccipal is well-developed, forming a triangular wall.

The laterosphenoid is observed in both sides (Figs. 6A, 6B, 7A and 7B). It is a relatively large bone, with a triangular shape that has two main surfaces. One faces anterolaterally and contributes to the posterodorsal orbital cavity, contacting dorsally the frontal and the postorbital. The other surface projects posterolaterally and constitutes the anterolateral wall of the cerebral cavity. This portion contacts dorsally the parietal and posteriorly the quadrate, throught an interdigitated and long suture. At its ventral point, this latter suture is enclosed by a foramen for the cranial nerve V. The prootic bone is not properly discernible.

The basisphenoid is well-preserved (Figs. 11A and 11B). It has an isosceles-trapezoidal shape, with a conspicuous triangular median depression, bounded by distinctive sharp crests. It is a relatively large bone that faces posteroventrally, similar to most notosuchians (e.g., *Sphagesaurus huenei*, Pol, 2003; *Y. boliviensis*, Novas et al., 2009; *Mariliasuchus amarali*, Zaher et al., 2006). The basisphenoid contacts anteriorly the pterygoids, posterolaterally the quadrates, and posteriorly the basioccipital, where the three Eustachian foramina are placed (Figs. 11A and 11B). The median Eustachian foramen (= foramen intertympanicum) is circular-shaped, enclosed between the basisphenoid and the basioccipital. The lateral Eustaquian foramina are oval shaped, positioned slightly posterior to the level of the median Eustachian foramen (Figs. 11A and 11B). Conspicuously, in *C. stenognathus*, the left and median Eustachian foramina are placed at the same line, as in other sphagesaurids (Pol et al., 2014). They are bounded anteriorly by the basisphenoid, medially and posteriorly by the basioccipital, and laterally there is a small contribution of the quadrate (Figs. 11A and 11B), a condition not seen in *C. stenognathus* (Pol et al., 2014).

The basioccipital faces posteroventrally and bears the occipital condyle. The condyle is subspherical, slightly dorsoventrally depressed. It has a conspicuous neck in its ventral and lateral bases. The basioccipital contacts dorsally the otoccipital, ventrolaterally the quadrate, and anteriorly the basisphenoid. Basal tuberae are not observed. In the area of the Eustachian foramina (see above), the basioccipital develops anteriorly two square-shaped processes between the median Eustaquian foramen and the lateral ones (Fig. 11A and 11B).

The right otoccipital is better preserved than the left one. They occupy most of the occipital face of the skull (Figs. 10A and 10B). The paraoccipital process is tall and bears a prominent, rounded crest that starts from the dorsolateral edge of the foramen magnum and projects laterally. Above this crest, the triangular surface of the otoccipital contacts dorsomedially the supraoccipital and dorsolaterally the squamosal. Ventrally, the otoccipital contacts the basioccipital and the quadrate (Figs. 10A, 10B, 11A and 11B). The ventrolateral-most edge of the paraoccipital process and the main body of the otoccipital form a distinctive notch, laterally closed by the quadrate, which includes the cranioquadrate foramen (Figs. 10A and 10B). Just lateral to the occipital condyle base there is a small, circular foramen for the anterior ramus of the hypoglossal cranial nerve (XIIa). More laterally, there is a broken, oval surface that bears an small foramen on its medial edge (considered the foramen for the posterior ramus of the hypoglossal cranial nerve –XIIp–) and the other portion should correspond to the metotic foramen for passage of the cranial nerves IX–XI and internal jugular vein (Figs. 10A and 10B). Just lateral to this broken area, there is another oval foramen, interpreted as the internal carotid foramen (Figs. 10A and 10B).

The palatines are two discrete bones in the palate of *C. mineirus* (Fig. 9). They are rod-like in shape, with the anterior processes contacting the maxillae and the posterior processes contacting the pterygoids. The main body constitutes the floor and lateral borders of the nasopharyngeal duct (Fig. 9). In the right side, its contact with the descending process of the prefrontal can be observed. The main body of the palatine defines the medial edge of the palatal fenestra. Anteriorly, the palatine has two distinctive process. An anterolateral process that contacts the maxilla and forms the posterior edge of the small maxillo-palatine fenestra, and an anteromedial one, that also contacts the maxilla and forms the medial edge of the aforementioned fenestra (Fig. 9). On the other hand, the posterior portion of the palatines form a thin and short posteromedial process, that contacts the pterygoid median septum of the choanae, as well as a thin laminar posterolateral process, that projects posteroventrally into the pterygoid wing (Figs. 9A, 9B, 11A and 11B). This latter process is only preserved on the left side. It is long and thin, and contacts the pterygoid wing and the posteroventral process of the ectopterygoid. The area of contact between these three bones is conspicuously small in comparison to the pterygoid–ectopterygoid contact. In *C. stenognathus* and *C. paulistanus*, the palatines have a relatively large contribution to this wing.

The pterygoids are complex bones. The main body contacts posterodorsally the basisphenoid and dorsolaterally the quadrate and the laterosphenoid (Figs. 9A, 9B, 11A and 11B). Its medial process is very thin and long, and constitutes the choanal septum. The posterior portion, one third of the septum, is a single bony lamina, whereas the anterior portion is divided and bears a V-shaped groove, similar to that described for *C. stenognathus* (Pol et al., 2014). The pterygoid flanges are stout and projects anteroventrally, with their ventral-most point positioned ventrally to the level of the quadrate condyle. There is a conspicuous constriction at their bases. The anterior surface of each pterygoid flange has an elevated pedicel to hold the posteroventral process of the ectopterygoid. This anterior surface also has a small contribution of the posterolateral process of the palatine. Just posterior to the suture with the ectopterygoid, the pterygoid flange is slightly concave. The pterygoids do not form part of border of the palatal fenestra (Fig. 9).

The ectopterygoids are well-preserved. The main body has well-developed anterior and posterior processes (Fig. 9). The anterior process projects medially until the maxilla, extending beyond the level of the mesial edge of the last tooth. Also, this process contributes to the posterior border of the alveolus of the last tooth, a condition not observed in *Caipirasuchus paulistanus*. The posteroventral process starts with a rod-like shaft, that then expands onto the pterygoid flange. The ectopterygoid flange is squared-shaped and the ventrolateral corner expands to form a well-preserved acute and long projection, in the right side of the skull (Fig. 9). The anteromedial surface of the ectopterygoid flange bears a subtle crest, which slopes medially. A medial projection forms the contact area with the palatine. In lateral view, the ectopterygoid projects posteroventrally, with the ventral-most point positioned at the level of the posterior edge of the orbit.

### Hyoid apparatus

**Figure 13:**
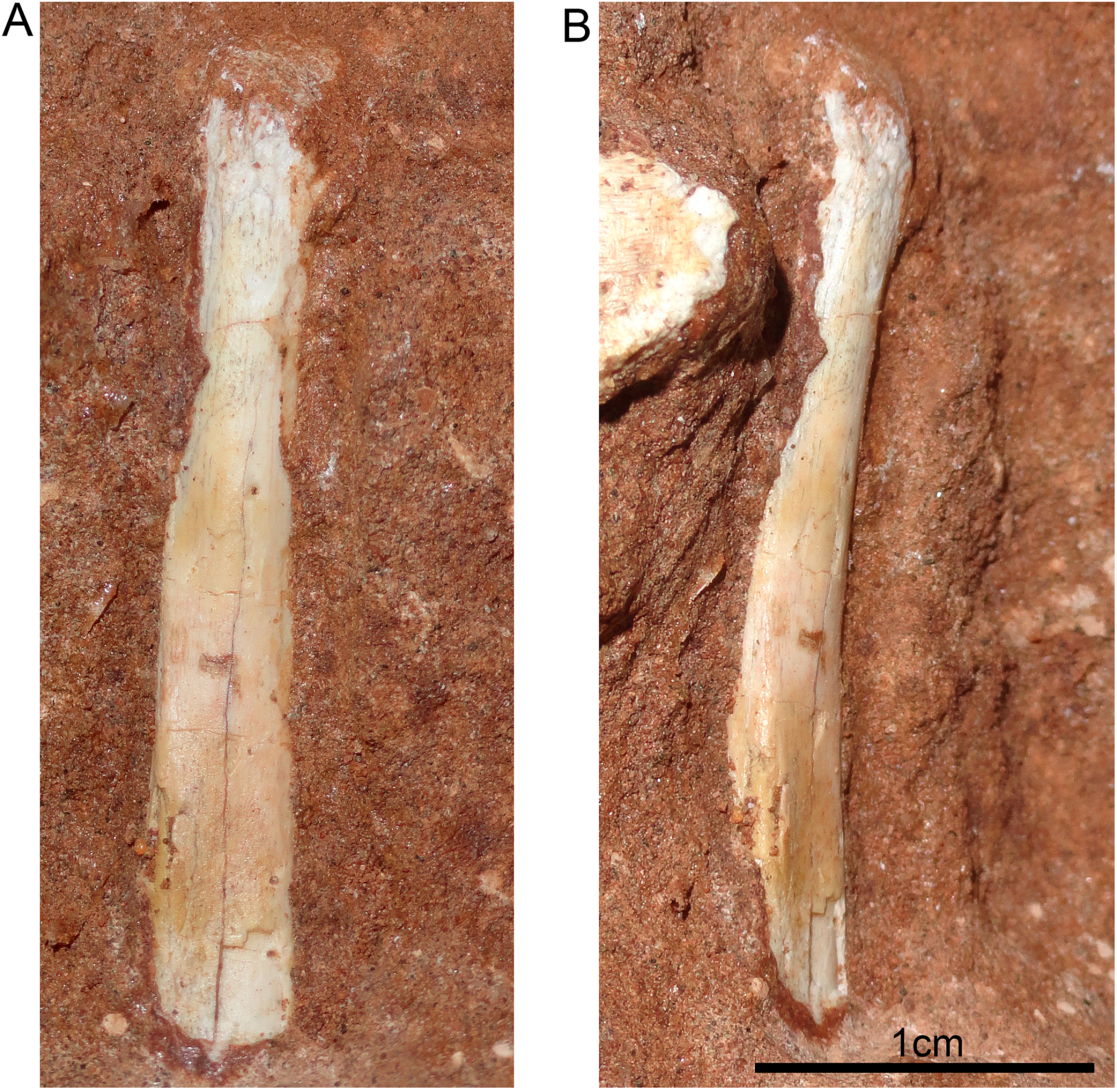
Hyoid apparatus of *Caipirasuchus mineirus* (CPPLIP 1463). First ceratobranchial in medial (A) and medioventral (B) views.

The hyoid bone is a long and flattened bone (Fig. 13A), which is slightly medially bent (Fig. 13B). Its proximal portion seems to be subcircular in cross-section, whereas its distal portion forms a flat and wide lamina, with almost parallel edges (Fig. 13A). For notosuchians, descriptions of hyoid apparatus are limited and based on elements referred as Ceratobranchialia I (e.g., *Simosuchus clarki*, Kley et al., 2010; *Baurusuchus albertoi*, Nascimento & Zaher, 2010) or Ceratohyalia (e.g., *Araripesuchus* spp., Turner, 2006). Accordingly to Schumacher (1973), the Cornu branchiale I is the only totally ossified element of the hyoid apparatus in crocodiles. Consequently, we consider this element as a Ceratobranchialia I, possibly the right element.

### Lower jaw

**Figure 14:**
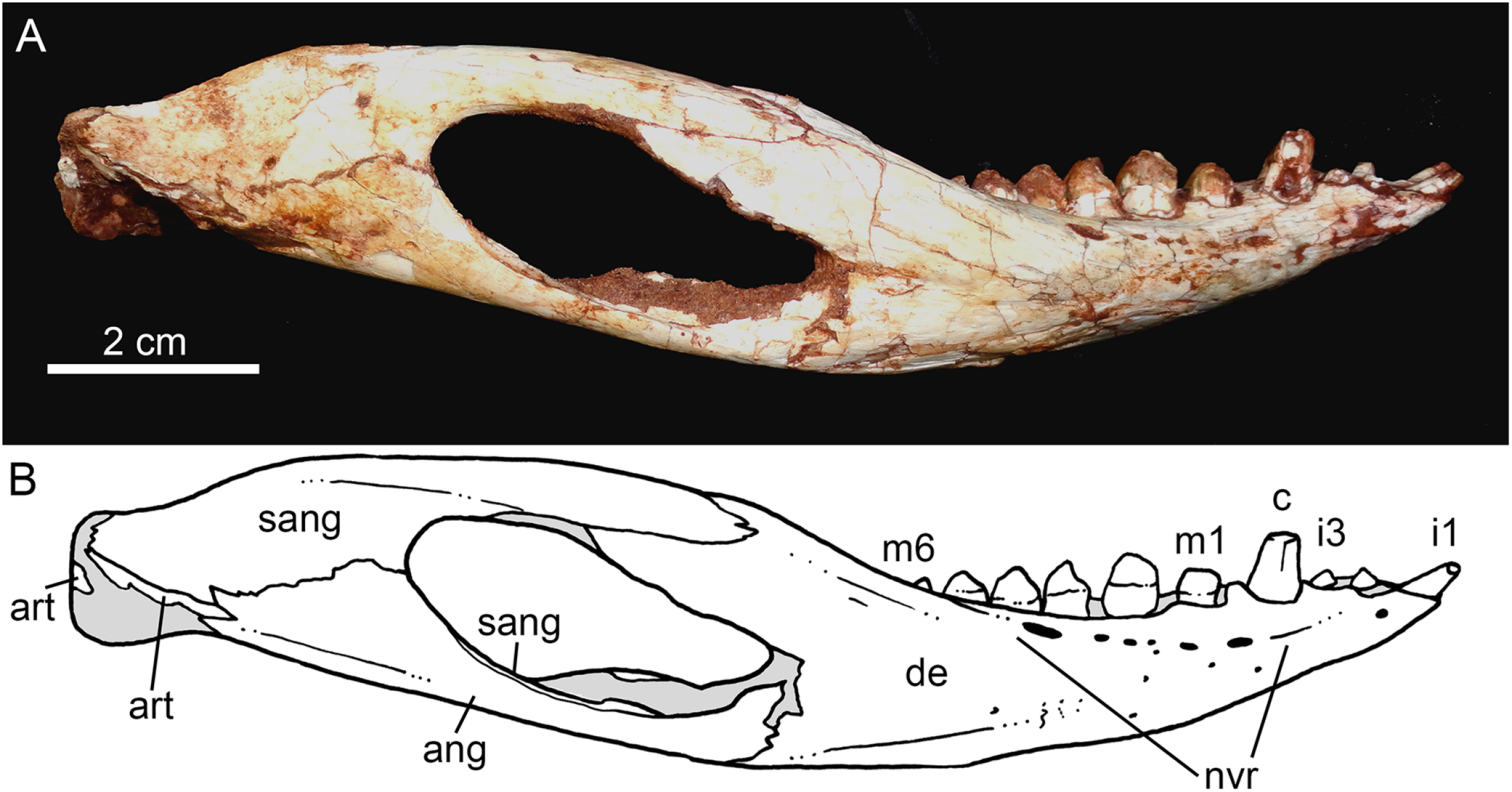
*Caipirasuchus mineirus*, CPPLIP 1463. Lower jaw in lateral view (A) with schematic drawing (B). Abbreviations: ang, angular; art, articular; c, lower caniniform; de, dentary; i, lower incisiviforms; m, lower molariforms; nvr, neurovascular foramina row; sang, surangular.

**Figure 15:**
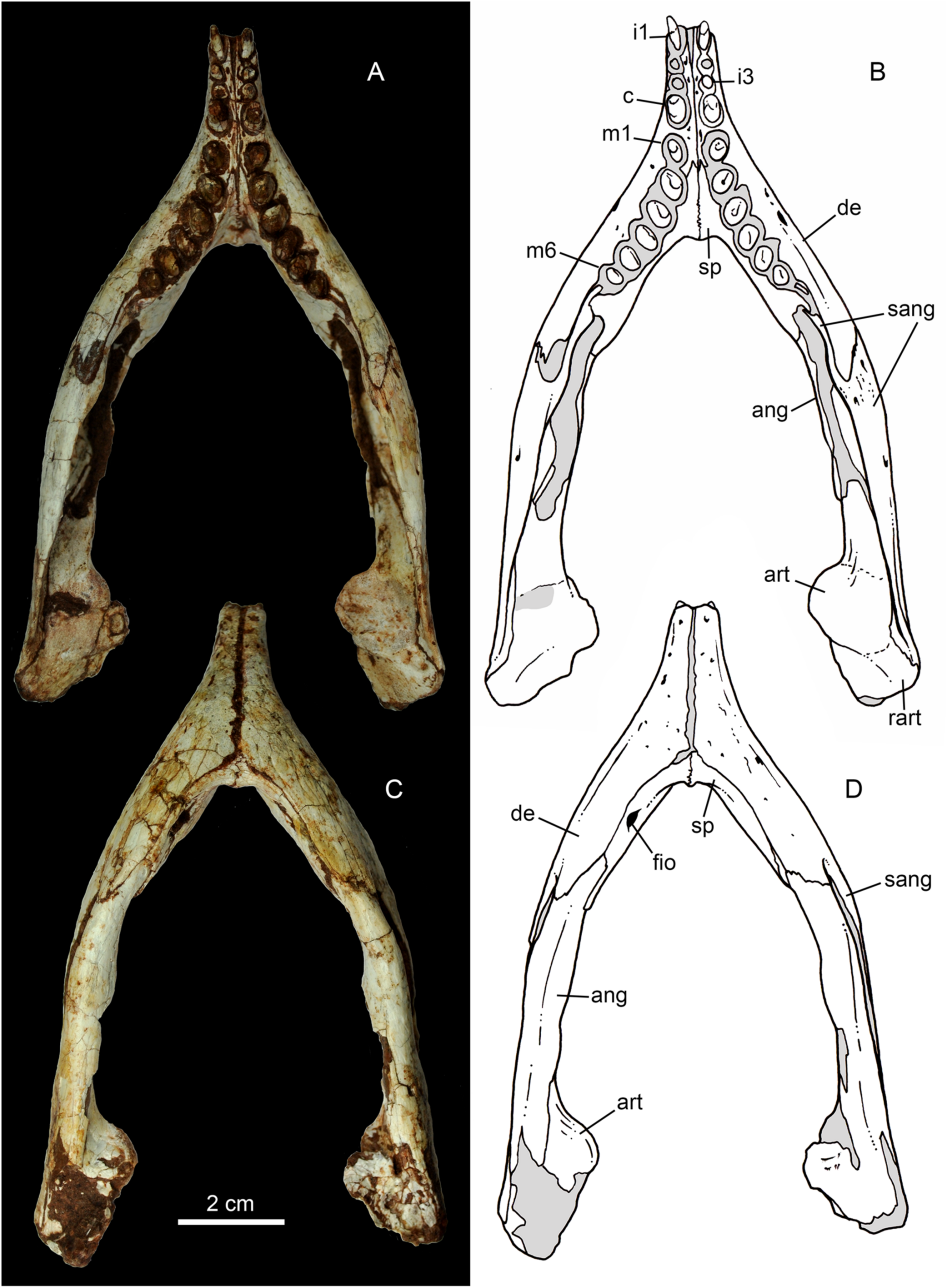
*Caipirasuchus mineirus*, CPPLIP 1463. Lower jaws with schematic drawings in dorsal (A and B) and ventral (C and D) views. Abbreviations: ang, angular; art, articular; c, lower caniniform; de, dentary; fio, foramen intermandibularis oralis; i, lower incisiviforms; m, lower molariforms; nvr, neurovascular foramina row; rart, retroarticular process; sang, surangular; sp, splenial.

Both hemimandibles are preserved (Figs. 14A, 14B and 15A–15D), sharing a similar morphology with other *Caipirasuchus* species (Iori et al., 2013; Pol et al., 2014; Iori & Carvalho, 2018). They are strongly attached to one another at the symphysis, which has a clear suture (Figs. 15A–15D). The symphysial suture is long and extends from the anterior tip of the lower jaw up to the level of the distal edge of the seventh tooth. The splenial constitutes the posterior one third of the symphyseal suture in dorsal view, but only one tenth in ventral view (Figs. 15C and 15D). The symphysis is narrow, with almost parallel edges, until the fourth tooth. Then the dentaries diverge posterolaterally, with an angle of aproximately 60°. Posteriorly to the toothrow, the lower jaws curve medially. Thus, in dorsal view, each dentary has a sigmoidal shape. The dentary forms a well-developed ventral surface, which is almost flat. There are several very small nutritious foramina, associated with shallow grooves. The lateral surface of the dentary is slightly convex and slopes medially, with an horizontal plataform that develops lateral to the tooth row, from the fourth to the tenth tooth (the last one) (Figs. 14A and 14B). This plataform results in a toothrow more medially positioned. From the the first to the fifth tooth, the dentary is extremely narrow, making both toothrows close to one another and to the median line. In lateral view, there is a line of seven foramina ventral to the toothrow, of which the first and the last are the larger ones. This line goes from the level of the medial edge of the forth tooth up to the eight tooth. Below this line, there are other randomly distributed foramina. The dentary forms the anterior half of the dorsal edge of the mandibular fenestra in lateral view (Figs. 14A and 14B). In medial aspect, the dentary does not contribute to the fenestra. The posterodorsal process of the dentary is tall and long, ending in two distinctive acute projections (Figs. 14A and 14B). The dorsal process projects posteromedially onto the surangular, and the ventral one sutures with the surangular, forming a V-shaped pattern, with the ventral process longer than the dorsal one. The posterodorsal process of the dentary has a larger contribution on the mandibular fenestra than in *C. stenognathus* (Pol et al., 2014). The posteroventral process of the dentary is short and displays on the angular bone. It does not form part of the mandibular fenestra.

**Figure 16:**
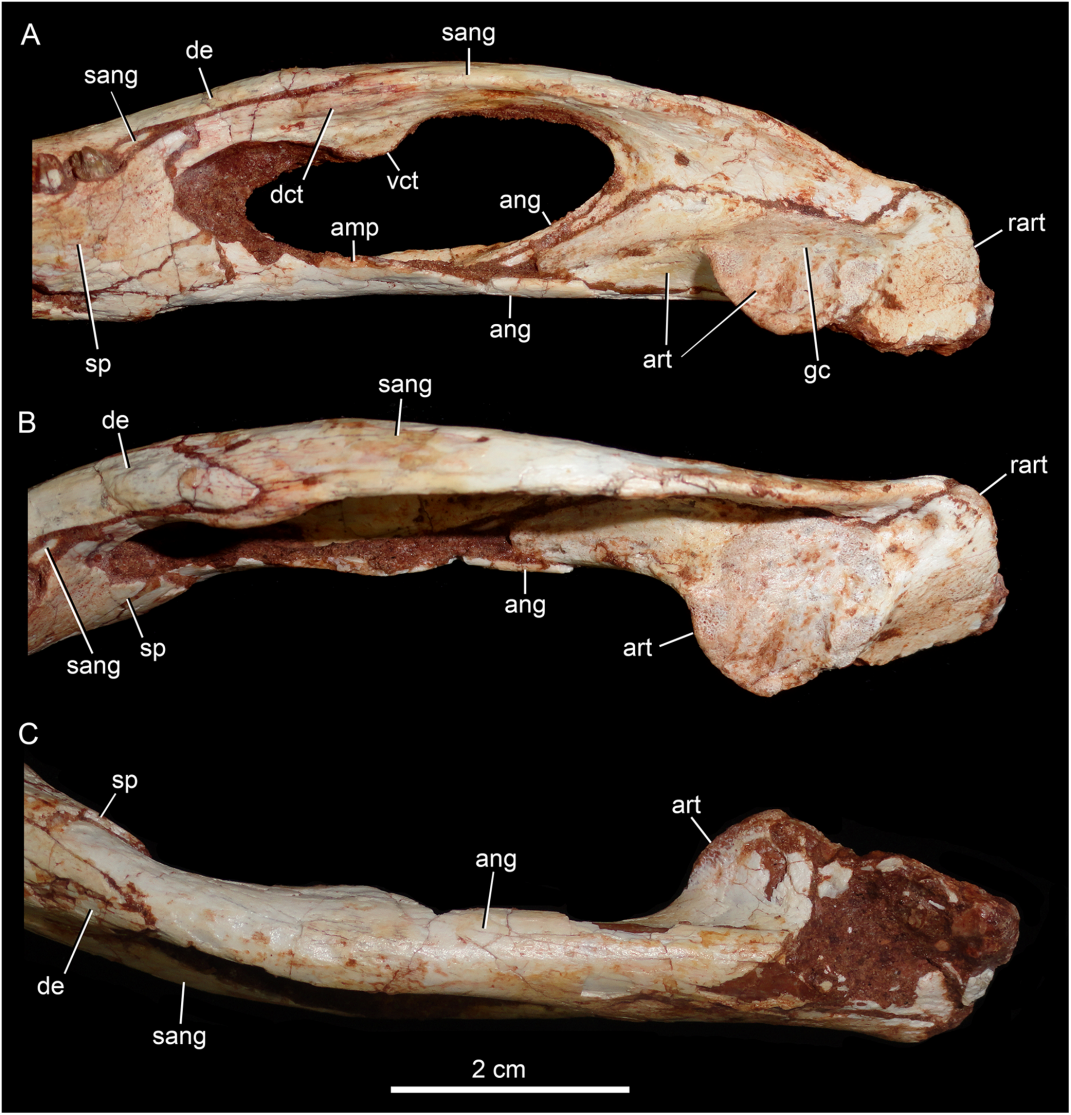
*Caipirasuchus mineirus*, CPPLIP 1463. Detail of posterior portion of right lower jaw in mediodorsal (A), dorsal (B), and ventral (C) views. Abbreviations: amp, ascending medial process of angular; ang, angular; art, articular; dct, dorsal coronoid tuberosity of surangular; de, dentary; gc, glenoid crest; rart, retroarticular process; sang, surangular; sp, splenial; vct, dorsal coronoid tuberosity of surangular.

The splenial covers most of the medial side of the lower jaw, from the symphysis to the anteroventral edge of the internal side of the mandibular fenestra. It is dorsoventrally tall and almost flat. The dorsal edge delimites the medial margin of the alveoli, and the splenial also contributes to form the medial interalveolar processes. The oval foramen intermandibularis oralis is placed below the level of eighth tooth, near the ventral edge of the jaw. The splenial has a small posterodorsal process that displays on the surangular, without touching the posterodorsal process of the dentary. The posteroventral process of the splenial rests on the angular and forms the internal anteroventral edge of the manbibular fenestra (Fig. 16A).

The surangular is a dorsally convex bone that links the articular area to the dentary bone (Figs. 15A–15D and 16A–16C). It forms the posterodorsal edge of the mandibular fenestra. It is dorsventrally convex above the fenestra and almost flat at its posterior portion. The posterior medial surface is thin and enclosed between the angular and articular bones. The anterior medial surface is separated from the lateral surface by a U-shaped sulcus. On the medial surface, just below the level of the posterodorsal process of the dentary, the dorsal and ventral coronoid tuberosities are observed. The former projects medially and the latter projects ventrally. Anteriorly, the surangular precludes the splenial–dentary contact, posterior to the toothrow, and it reaches the posterior border of the last alveolus (Fig. 16A).

The angular forms the ventral edge of the mandibular fenestra (Figs. 14A, 14B, 15C, 15D and 16A–16C). It wedges anteriorly between the dentary and the splenial. Its ventral surface is convex and defines an internal U-shaped longitudinal groove, which corresponds to the adductor mandibular fossa. There is a sharp crest, the angular crest, on the lateral edge of the angular, just at the middle of the mandibular fenestra. Posteriorly to the fenestra, the angular expands dorsoventrally to contact the surangular and the articular (Fig. 16A).

The articular is the more robust bone of the postdentary complex. It rests medially to the surangular, with which it forms a long suture, and dorsally to the angular. The glenoid region projects medially as a large subcircular surface bearing a subrectangular retroarticular process (Figs. 15A–15D and 16B). The glenoid area is slightly dorsally convex and there is no well-defined crest, possibly due to bad preservation. The retroarticular process is better preserved in the right side. It is squared-shaped, about two times transversely broader than anteroposteriorly long. It has a small lateral flange, with a shallow concavity (Figs. 16A and 16B). The medial flanges project posteroventrally and face posteromedially. In the right side, there is medial notch separating the glenoid region and the retroarticular process. This notch, not observed in other *Caipirasuchus* species, would be a preservational artifact. The ventral surface of both articular bones are broken.

### Dentition

**Figure 17:**
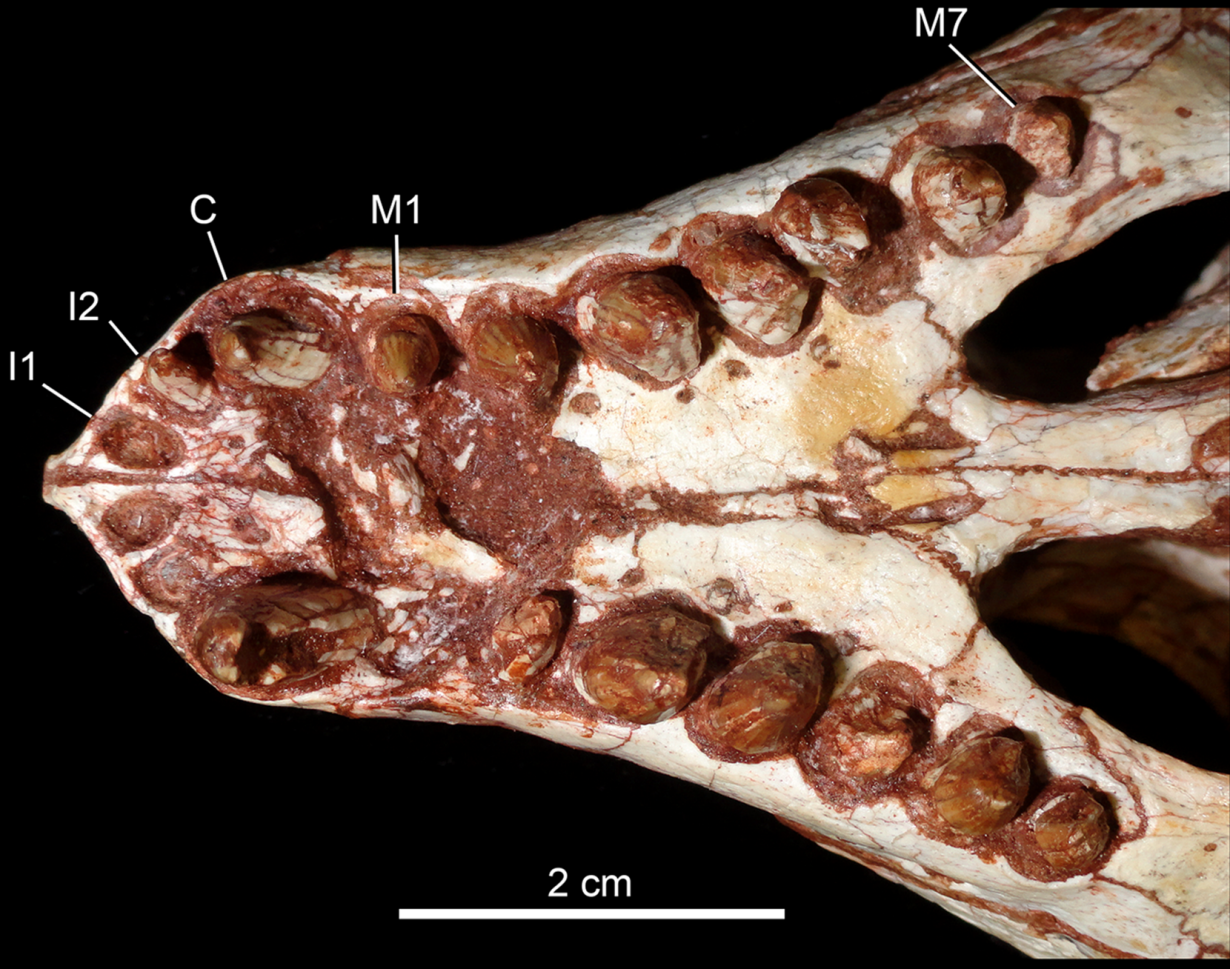
*Caipirasuchus mineirus*, CPPLIP 1463. Detail of the upper dentition in occlusal view. Abbreviations: C, upper caniniform; I, upper incisiviforms; M, upper molariforms.

**Figure 18:**
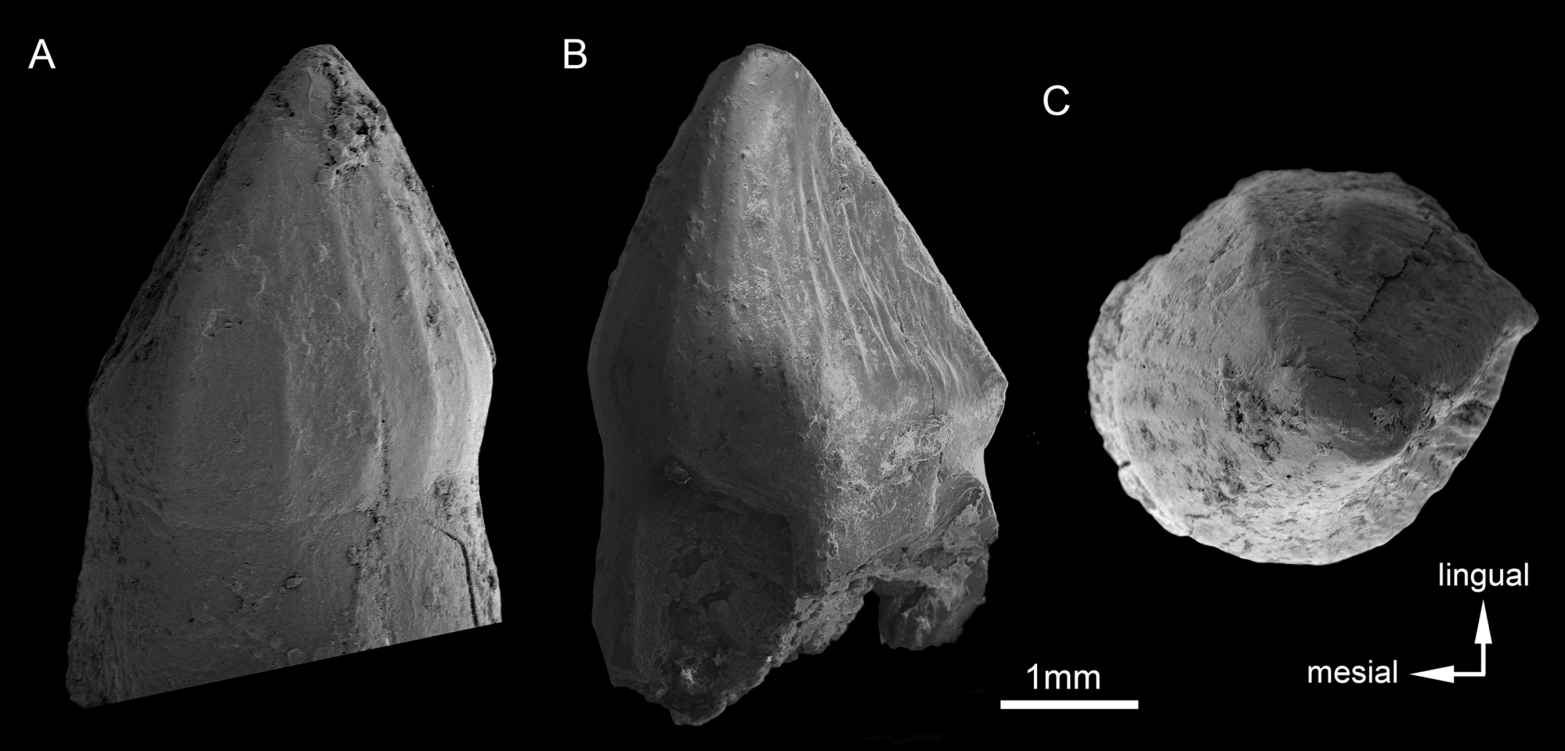
*Caipirasuchus mineirus*, CPPLIP 1463. SEM photographs of first right upper molariform tooth (M1) detached during preparation in mesiolabial (A), distolingual (B), and occlusal (C) views.

The dentition of *C. mineirus* is not very well-preserved, but the general morphology can still be accessed (Figs. 17 and 18). There is a conspicuous heterodonty in this taxon, as in other “advanced notosuchians” (Lecuona & Pol, 2008; De Andrade & Bertini, 2008c; Pol et al., 2014), which allow us to differentiate them in incisiviforms (I/i), caniniforms (C/c), and molariforms (M/m), also known as “sphagesauriform teeth” (Iori & Carvalho, 2018).

**Upper dentition.** The upper dentition consists of four premaxillary teeth and six maxillary teeth in each side (Fig. 17). The first incisiviform tooth is not preserved on either side, and the second one is only preserved on the left side. However, based on the sizes of these teeth alveoli, as well as on the size of the left I2, they represent the upper incisiviforms. The left I2 is conical, without a constricted root, and considerably smaller than the remaining teeth. It is implanted in a vertical position. The third tooth represents the caniniform (Fig. 17). It is the largest tooth of the upper series, even larger than any tooth in the lower jaw. It is a subconical tooth, transversely compressed, with an oval cross-section at the base. There is no carinae in either the mesial nor the distal edges. The mesial edge is gently convex whereas the distal one is slightly concave. The fourth and last premaxillary tooth (i.e., M1) was interpreted as a transitional tooth in other species of *Caipirasuchus* by Pol et al. (2014). It is considerably smaller than the caniniform, with almost the same size of the first molariform of the maxilla (i.e., M2). This tooth is only preserved on the left side of the skull. The crown is poorly preserved. It is conical and there is evidence of a broken crest on the mesiolingual corner of the crown. The crown is wide at the base and its ridges on the enamel layer are shallower in comparison with those present in more posterior teeth. The right M1 was detached during mechanical preparation of the skull, thus it is isolated (Figs. 18A–18C). It is almost circular in cross section at its crown base, with convex mesial and concave distal surfaces. It has a conspicuous triangular wear facet on the linguodistal crown wall, without evidence of denticules. The mesial and mesiolabial portions of the crown wear spaced ridges on the enamel (Fig. 18A). However, on the labiodistal surface, the enamel is more rugose, with closely positioned ridges. The wear facet is large, extended from the apex to the crown base, exposing the dentine (Fig. 18C). In the maxilla, M2, M3, and M4 are the largest molariforms, M1 and M5 have similar sizes, and M6 is the smallest (Fig. 17). The molariforms are drop-shaped in cross-section, with the major axis anterolaterally to posteromedially oriented, and the sharp corner pointing distolingually.

The molariform tooth rows diverge slightly posteriorly, but in a lesser degree than in the lower jaw. In all molariforms the crowns are bulbous, with a narrower root. Most crowns are poorly preserved, but the basic plan consists of a main bulbous cusp, positioned in the mesiolabial side of the crown, with a conspicuous crest bearing discrete cusps that descend distolingually. The main conical cusp has a strongly convex mesiolabial surface, and the descending crest defines both the mesiolingual and the distolabial surfaces. Conspicuous parallel ridges ornamentate the enamel layer. These are more marked on the lingual half of the crown. The first left maxillary tooth (M2) is the best preserved molariform, which have the aforementioned features. In this element, the crest has at least five discrete cuspules, that decrease in size toward the base (opposite to the condition of the lower molariforms, see below). This cuspules are eroded, but seem to be conical.

Wear facets are properly observed in the isolated right M1, right M5 and in the left M1, M2, and M5 (Fig. 17). In the remaining teeth, it is difficult to access this information due to bad preservation. The left caniniform is not fully erupted, as occurs in the right M4.

**Figure 19:**
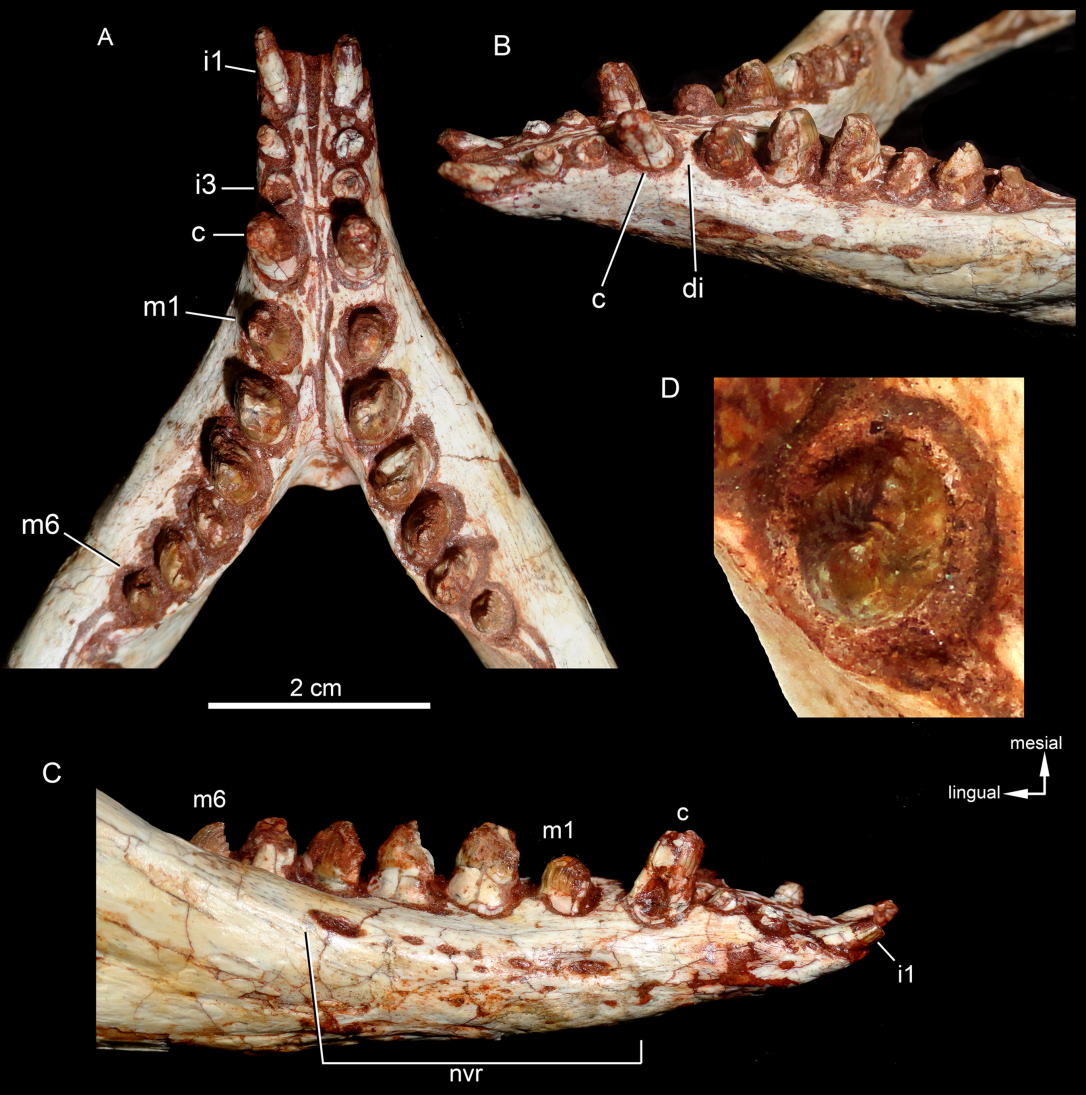
*Caipirasuchus mineirus*, CPPLIP 1463. (A) Detail of lower left and right tooth rows in occlusal view. (B) Left tooth row in laterodorsal view. (C) Left tooth row in lateral view. (D) Sixth right molariform in occlusal view. Abbreviations: c, lower caniniform; di, diastema; i, lower incisiviforms; m, lower molariforms; nvr, neurovascular foramina row.

**Lower dentition.** There are 10 teeth in each lower jaw (Fig. 19A). The first three teeth are considered incisiviforms. They have conical crowns and are smaller than the caniniform (fourth tooth). Tooth i1 is larger than teeth i2 and i3 and procumbent, whereas i2 and i3 are vertically positioned (Figs. 19A–19C). Tooth i1 seems to be relatively larger than in the other species of *Caipirasuchus*, but it is still comparatively smaller than in *Y. boliviensis* (Novas et al., 2009) and *Adamantinasuchus navae* (UFRJ-DG 107-R; Nobre & Carvalho, 2006). Based on the preserved crowns of left i1 and i2, there is no carinae on the crown, either there is a neck between the crown and the root. The fourth tooth is considered a caniniform (Figs. 19A–19C). It is conical and considerably taller than the previous i1–i3 teeth, and also than m1, with the crown slightly anterodorsally inclined. The caniniform exposes part of its root which is continuous with the crown, without constriction or neck. The first four dentary teeth (i.e., from the i1 tooth until the caniniform) have merged alveoli. They have interalveolar processes delimiting each alveolus, but these processes do not contact one another at the midpoint, precluding a closed alveolus. Posterior to the caniniform there is an small diastema formed by a clear dentary ridge (Fig. 19B). This diastema is the only point in the tooth row where the left and right interalveolar processes meet one another. This diastema is smaller than the space for a regular tooth (e.g., m1–m6). The last six teeth are considered molariforms, but the first tooth has a simpler morphology than the remaining ones. It has an oval shape, with a rounded crown at the base and a central cusp that bears longitudinal ridges. Teeth m2–m6 are drop-shaped at cross-section, with major axis anterolaterally to posteromedially oriented, and the sharp edge pointing mesiolabially (Fig. 19A). They increase in size until the m3, and then decrease considerably, with m6 being smaller than m1. Tooth m1 is slightly separated from m2, but there is not a real diastema and the alveolus is not completely closed. From m2 to m6, the teeth are more closely spaced, and the sharp edge of a posterior tooth displays lateral to the distal part of the preceding tooth, as observed in many “advanced notosuchians” (Gasparini, 1971; Bonaparte, 1991; Pol et al., 2014). The molariform row is slightly arched, with the last teeth pointing posterolaterally. In all molariforms, the crown is slightly bulbous, forming a conspicuous step at the contact with the root. Unfortunately, the crown of most molariforms are poorly preserved, precluding the recognition of several features present in sphagesaurids (Pol, 2003; Novas et al., 2009; Iori et al., 2013; Pol et al., 2014; Iori & Carvalho, 2018). The basic plan is the presence of a main bulbous cusp positioned at the distolingual side of the crown, with a conspicuous crest that descends mesiolabially and defines two slightly concave surfaces, on the mesiolingual and distolabial sides. Nonetheless, the right m6 preserves the crown better than in the remaining teeth (Fig. 19D). It has a conical cusp on the distolingual side of the crown, and a cuspidate crest that descends mesiolabially. The main cusp has a strongly convex distolingual surface, and tapers toward the base. The crest bears five discrete cuspules in addition to the main cusp. The cuspules increase in size toward the base, but the basal-most cuspule is slightly smaller than the preceding one (Fig. 19D). The cuspules are conical, without distinctive carinae or ridges. The crest divides two surfaces. The mesiolingual surface has an abrupt angulation between the crest and the rest of the crown, giving it a deeply concave area. Differently, the distolabial surface is more vertically oriented. Both surfaces have deep and parallel grooves on the enamel surface. Such ornamentation is very shallow to inexistent in the distolingual surface of the main cusp (Fig. 19D).

Along the tooth rows, apical wear is observed in the main cusp of right and left m2 and a large wear facet expands on the distolingual surface of the crown of both right and  left m5, suggesting a similar occlusal pattern between right and left sides. The left m4  seems to be not completely erupted, although the crown is not well-preserved and the last right and left teeth seems to represent newly erupted molariforms. Finally, between right i1 and i2, there is an small section of tooth that can be a replacement element or the tip of an upper tooth, as before preparation the lower jaws were occluded with the skull.

The presence of a large fourth tooth in *C. mineirus* (larger than i1–i3 and m1, and interpreted here as a caniniform tooth) indicates a different condition to that of *C. stenognathus*, in which the first five teeth increase in size gradually. Also, in *C. stenognathus*, the fifth tooth is implanted in an isolated alveolus, with small anterior and posterior diastemata. Contrarily, in *C. mineirus* the posterior diastema is not present and the alveolus of the fifth tooth (i.e., m1) is collapsed with that of the remaining molariforms.

### Postcranium

**Figure 20:**
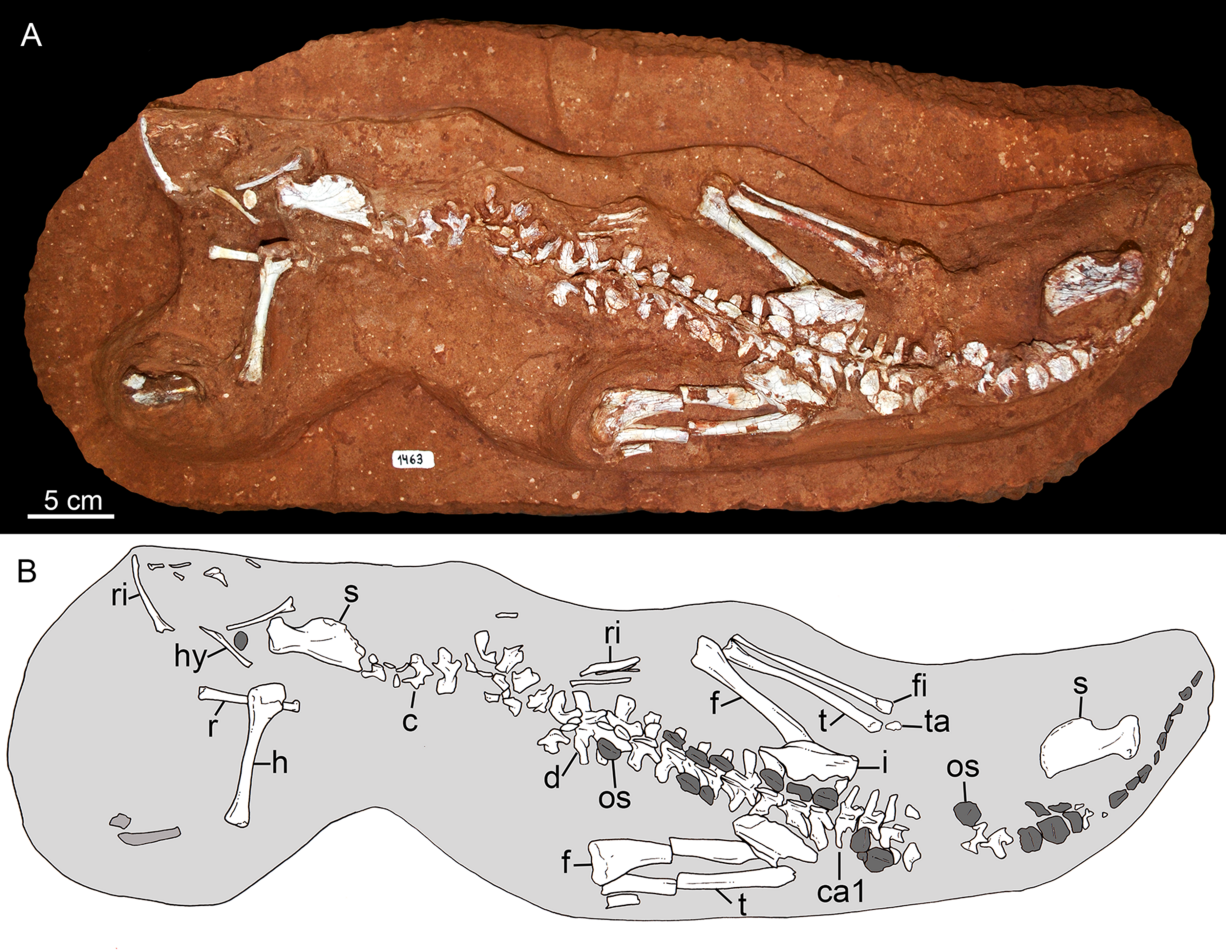
*Caipirasuchus mineirus*, CPPLIP 1463. Postcranial skeleton (the skull was removed) (A) with schematic drawing (B). Abbreviations: c, cervical vertebra; ca, caudal vertebra; d, dorsal vertebra; f, femur; fi, fibula; h, humerus; ta, tarsal bones; hy, hyoid bone; il, ilium; os, osteoderm; r, radius; ri, rib; s, scapula; t, tibia; ta, tarsal bones.

The holotype of *C. mineirus* includes a partially articulated skeleton, still resting on the rock matrix (Figs. 20A and 20B). It consists of 16 articulated presacral and three sacral vertebrae, complete tail caudals (covered with osteoderms), about 30 osteoderms, five ribs, left humerus, left radius, both scapulae, both femora, both tibiae, complete right fibula, proximal part of left fibula, both ilia, right calcaneum, distal tarsal 4, and metatarsal V. The vertebral column is preserved in an arched position, with the tail twisted to the right side (Figs. 20A and 20B). The hindlimbs are flexed with the tibia and fibula parallel to the femur. The anterior half of the skeleton suffered more disarticulation than the posterior one. Most of the cervical region, the right forelimb and part of the left forelimb are missing. Only a few ribs are preserved. The right scapula is near the left humerus, but the left scapula is resting above the middle of the tail (Figs. 20A and 20B). Other shoulder girdle bones are not preserved. From the pelvic girdle, only right and left ilia are partially preserved.

### Presacral vertebrae

**Figure 21:**
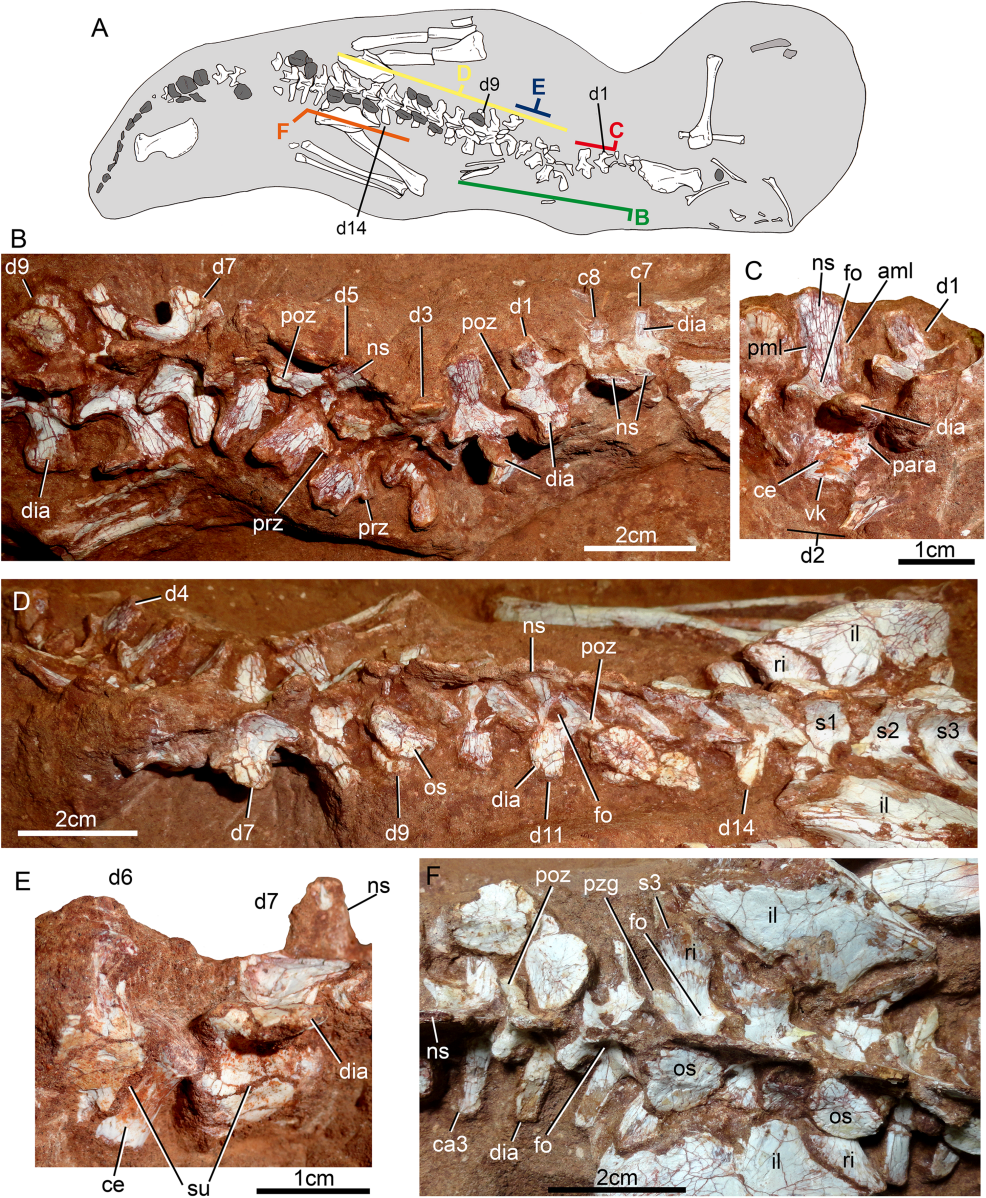
Vertebral column of *Caipirasuchus mineirus*, CPPLIP 1463. Skeleton indicating the position of the illustrated vertebrae (A). Last cervical (c7–c8) to ninth dorsal (d9) vertebrae as positioned in the block (B). Detail of first (d1) and second (d2) dorsal vertebrae in right lateral view (C). Fourth (d4) to third sacral (s3) vertebrae as positioned in the block (D). Detail of partial sixth (d6) and seventh (d7) vertebrae in left lateral view (E). Detail of sacral and first caudal vertebrae in dorsal view (F). Abbreviations: aml, anterior medial lamina; c, cervical vertebra; ca, caudal vertebra; ce, vertebral centrum; d, dorsal vertebra; dia, diapophysis; fo, fossa; il, ilium; ns, neural spine; os, osteoderm; para, parapophysis; pml, posterior medial lamina; poz, postzygapophysis; prz, prezygapophysis; ri, rib; s, sacral vertebra; su, neural suture, vk, ventral keel.

CPPLIP 1463 preserves 16 articulated presacral vertebrae (Figs. 20A, 20B and 21A). We considered the first two preserved vertebrae as cervical seventh (c7) and eight (c8), whereas the following vertebrae are considered dorsals, from d1 to d14. They are partially exposed and only in d2, d6, and d7 the vertebral centra are observed (Figs. 21C and 21D). They show unfused neural central sutures between the arch and the centrum, possibly indicating a subadult condition of the individual (CA Brochu, 1992, unpublished data; Irmis, 2007). This is also supported by the lack of fussion between diapophyses and their respective rib in sacral vertebrae, between sacral ribs and ilium, and the relatively small size of the specimen, but further studies (e.g., bone histology) are needed to settle this issue.

The cervical series is poorly known, as only part of the neural arch of c7 and c8 is preserved. They are smaller than d1, with a transversely wide and anteroposteriorly short neural arch (Fig. 21B). The diapophyses are laterally well-projected and anteroposteriorly short, forming a laminar process. The pre- and postzygapophyses are well separated from the midline of the arch. There is a concave notch between the prezygapophysis and the neural spine, whereas the postzygapophysis connects the spine by means of a thin lamina. The neural spines of both vertebrae are dorsally incomplete, although laminar, and less posteriorly inclined than the first dorsals. Evidence of spinoprezygoapophyseal and spinopostzygoapophyseal laminae is limited due to the preparation of the specimen. There is no well-marked anterior and posterior medial laminae as in d2. The neural spine of c7 is slightly anteroposteriorly shorter than in c8 (Fig. 21B).

The dorsal series is almost complete, including 14 articulated vertebrae, with some elements partially preserved and still within the rock matrix, limiting the access to their information. The series increases in size posteriorly, being d3–d9 the largest vertebrae (Figs. 21B and 21D). The anterior dorsals seem to be taller than the posterior ones, which are transversely broader and dorsoventrally lower.

The vertebral centra are is visible in d2, d6, and d7 (Figs. 21C and 21E). It is anteroposteriorly shorter in d2 than in d6–d7, and in the former there is a ventral keel not seen in d6–d7. In d2 the lateral surface of the centrum is slightly excavated, whereas in d6–d7 it is convex. The neurocentral suture is also visible in these three vertebrae. In d2, a broken parapophysis is seen just at this point (Fig. 21C), as occurs in anteriormost dorsal vertebrae of some notosuchians (e.g., *N. terrestris*, *Sebecus icaeorhinus*, *Y. boliviensis*; Fiorelli & Calvo, 2008; Pol et al., 2012; Leardi et al., 2015b). A parapophysis is not observed in the centrum of d6–d7.

The neural spines are not properly preserved in the sequence, especially in the middle and posterior portions. Vertebra d2 exhibits the largest spine, and spines seem to decrease in size posteriorly. In d2, the neural spine represents half of the vertebral height. It is very narrow transversely, almost laminar, but it is also anteroposteriorly broad. There is no evidence for a dorsal expansion in the neural spines. There are conspicuous anteromedial and posteromedial laminae extended along the spines (Fig. 21C). Moreover, there is a lamina connecting the postzygapophysis to the spine (i.e., spinopostzygoapophyseal lamina) that extends dorsally until midheight. The neural spine of d2 is vertically positioned and seems to be slightly posteriorly oblique in successive elements.

The pre- and postzygapophyses are widely spaced from the midline. They are placed at almost the same level, especially in the anterior dorsals. The anterior dorsals (e.g., d1–d2) have pre- and postzygapophyses positioned above the level of diapophysis, whereas posteriorly these three structures are more leveled. The articular surface of the prezygapophysis is not clearly seen in most elements, but in d1 and d5 it is straight and faces ventrally (Fig. 21). The postzygapophyses also have a flat articular surface, facing dorsally. Particularly, in mid and posterior dorsals (e.g., d7) the postzygapophyseal processes are long and are posterolaterally projected. The postzygapophysis and the diapophysis form a wide plataform in dorsal view, larger in mid and posterior dorsal vertebrae, with a posterolaterally concave edge. On this platform, between the base of the postzygapophysis and diapophysis and near the base of the neural spine, there is a conspicuous fossa that becomes deeper posteriorly (Fig. 21F), similar to the condition of most other notosuchians (e.g., *N. terrestris*; Pol, 2005; Turner, 2006; Pol et al., 2012) but not seen in *Y. bolivienis* (Leardi et al., 2015b). The diapophyses (transverse processes in mid and posterior dorsals by the junction of diapophysis and parapophysis) are laterally shorter and anteroposteriorly longer in mid- and posterior dorsals than in anterior ones.

**Sacral vertebrae.** There are three sacral vertebrae (s), in addition to their sacral ribs, articulated to the ilia (Fig. 21F). Only part of the neural arches are prepared; thus, the fusion between s2 and s3 is difficult to evaluate. This makes it impossible to assert if the condition is similar to that of *N. terrestris*, in which these vertebrae are fused (Pol, 2005; Fiorelli & Calvo, 2008). However, the number of sacral vertebrae in *C. mineirus* is clear (i.e., three vertebrae), due to their direct articulation with the ilia. The neural spines are low, as in the posterior dorsals, and considerably anteroposteriorly long (Fig. 21F). Due to its anteroposterior length, the neural spine of s2 contacts that of s3. The postzygapophyses are seen in s3, similar in orientation to those of the last dorsal. There is a fossa anterior to the postzygapophyses and lateral to the base of the neural spine, which is still seen in the sacral vertebrae, but it is shallower than in the dorsal vertebrae. The transverse processes are subrectangular in dorsal view and are articulated with the sacral ribs by means of an interdigitated suture. The sacral ribs contact the ilia, but are not fused. In s1, the ribs are triangular and point anteriorly, with a large and oblique suture with the anteromedial wall of the iliac blade (Fig. 21F). The sacral ribs of s2 and s3 are less acute than that of s1 and point posteriorly. The transverse process of s2 and s3, as well as their ribs, are parallel to one another and projected posterolaterally, whereas in s1 the transverse process and the rib are projected anterolaterally, delimiting a rounded space between processes of s1 and s2. The vertebral centra cannot be accessed as the vertebrae are still in articulation.

**Caudal vertebrae.** The information on caudal vertebrae (ca) is mostly limited to ca1 to ca3, and the exact number of caudals is unknown. Based on the posteriormost distribution of osteoderms (i.e., at the tip of the tail) (Figs. 20A and 20B), we can speculate that the tail represented less than the half of total body length.

The first caudal is articulated to the sacrum and it seems to be slightly smaller than ca2. The transverse processes are long and anteroposteriorly narrow, posterolaterally projected in ca1 and laterally projected in ca2 and the following vertebrae (Fig. 21F). The pre- and postzygapophyses are also widely spaced from the midline of the vertebral series, as in the other vertebrae (Fig. 21F). The postzygapophyseal processes are long and posterolaterally projected, starting from the base of the neural spine, without a connecting lamina. The neural spines are also laminar, but anteroposteriorly less developed than in the sacral and dorsal vertebrae. Particularly in ca3, the tip of the neural spine seems to be slightly expanded laterally, as this is the portion of the vertebral series with the largest osteoderms (Fig. 21F).

### Ribs

**Figure 22:**
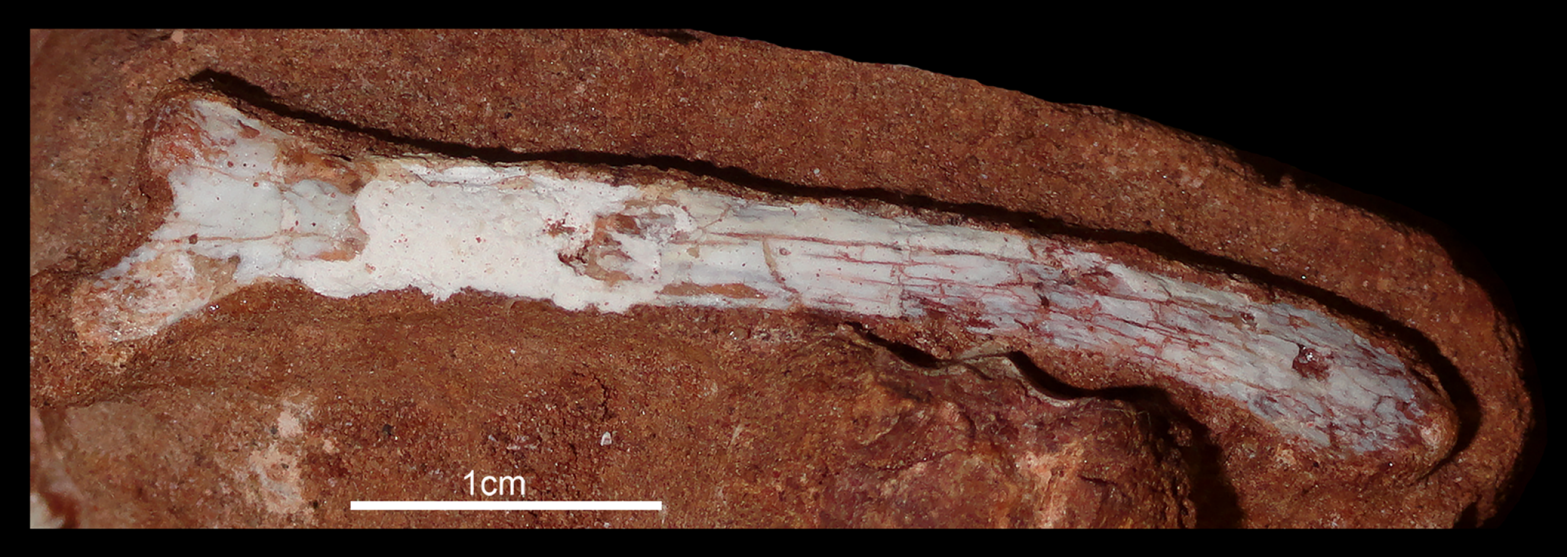
*Caipirasuchus mineirus*, CPPLIP 1463. Left rib of the dorsal series in lateral view.

Dorsal ribs are partially preserved. They are wide and blade-shaped, with almost parallel anterior and posterior edges that expand slightly at the the distal-most portion (Fig. 22). The lateral and medial surfaces are almost flat. The tuberculum and capitulum are separated by a distinctive U-shaped notch (Fig. 22).

### Osteoderms

**Figure 23:**
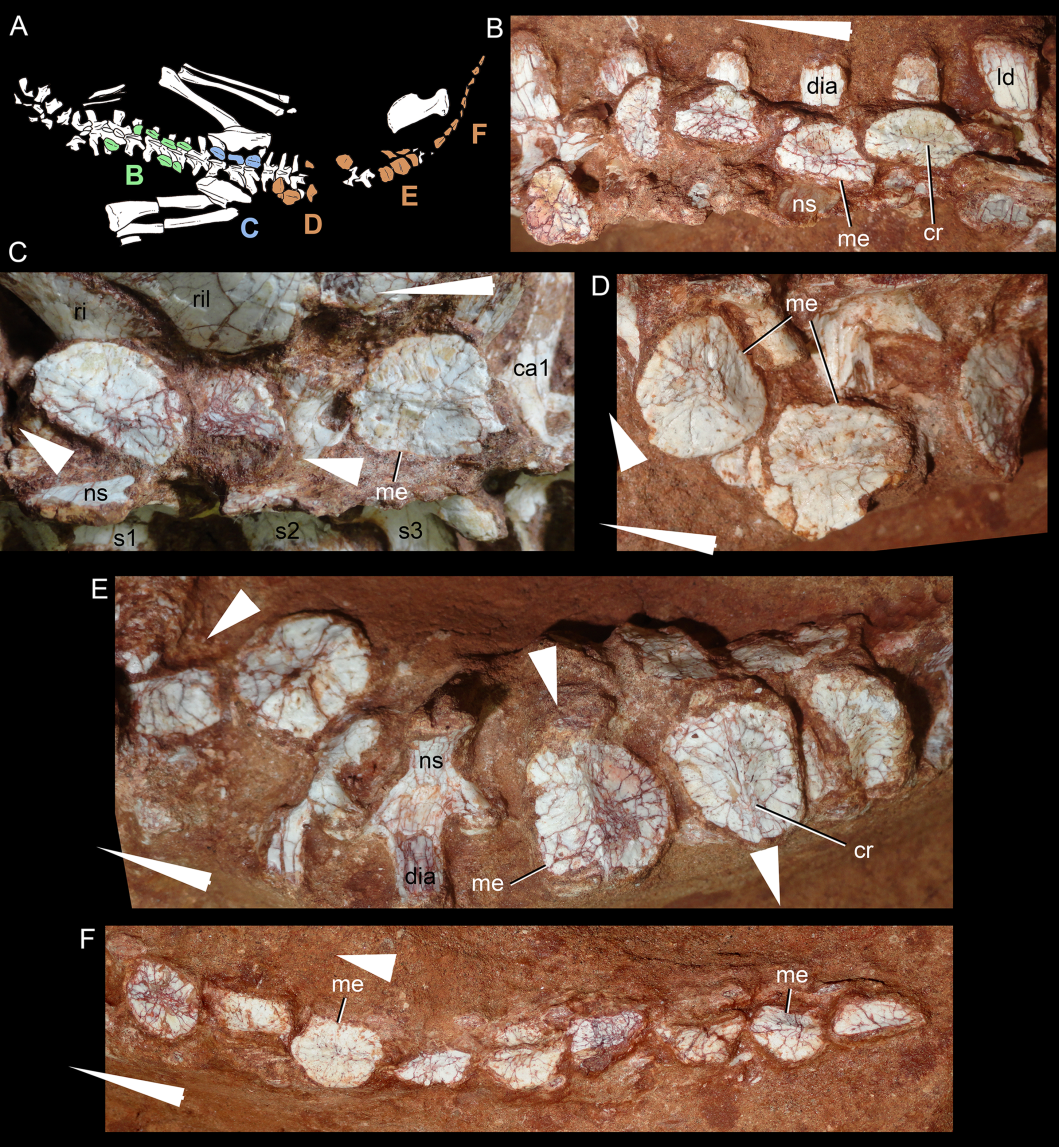
Osteoderms of *Caipirasuchus mineirus*, CPPLIP 1463. (A) The paramedial osteoderms. They are divided in parts as shown in (A): (B) presacral. (C) Sacral. (D) Anterior part of the tail. (E) Middle part of the tail. (F) Posterior part of the tail. Abbreviations: ca1, first caudal vertebra; cr, longitudinal crest; dia, diapophysis; ld, last dorsal vertebra; me, medial edge; ns, neural spine; ri, rib; ril, right ilium; s1–3, sacral vertebra 1–3. The long arrows indicate the anterior part of the animal, whereas the short arrows indicate the anterior side of a particular osteoderm.

There are almost 30 disarticulated osteoderms, next to the neural arches. Even having a continuous distribution, some of the osteoderms are rotated or slightly shifted (Figs. 20A, 20B and 23A). Osteoderms are restricted to the last six dorsal vertebrae (starting from d9), the sacrum and all the caudal region. Their distribution is remarkable because there is no evidence of osteoderms in the anterior half of the body, in opposition to what is observed in other *Caipirasuchus* species in which postcranial remains are known (i.e., *C. paulistanus* and *C. montealtensis*; Iori, Marinho & Carvalho, 2016) and have osteoderms all along the vertebral series. The absence of other bones of the shoulder girdle, forelimbs, and cervical elements could indicate that the anterior osteoderms were also lost during the taphonomic processes. Nonetheless, the osteoderms present on the posterior dorsal vertebrae are considerably smaller than the sacral and anterior caudal osteoderms, indicating a reduction in size toward the anterior portion of the body, at the posterior thoracic region. Examination of a *Caipirasuchus* specimen from São Paulo (MPMA 07-0011/00; Iori, Marinho & Carvalho, 2016) shows that osteoderms above dorsal vertebra are the largest of the series, squared-shaped, differing considerably from the condition preserved in *C. mineirus*. Such differences are here considered of taxonomic significance for *C. mineirus* and we considered, with the evidence at hand, that the lack of osteoderms in the cervical and anterior half of the thoracic region is a diagnostic feature of this species (see also Discussion).

**Figure 24:**
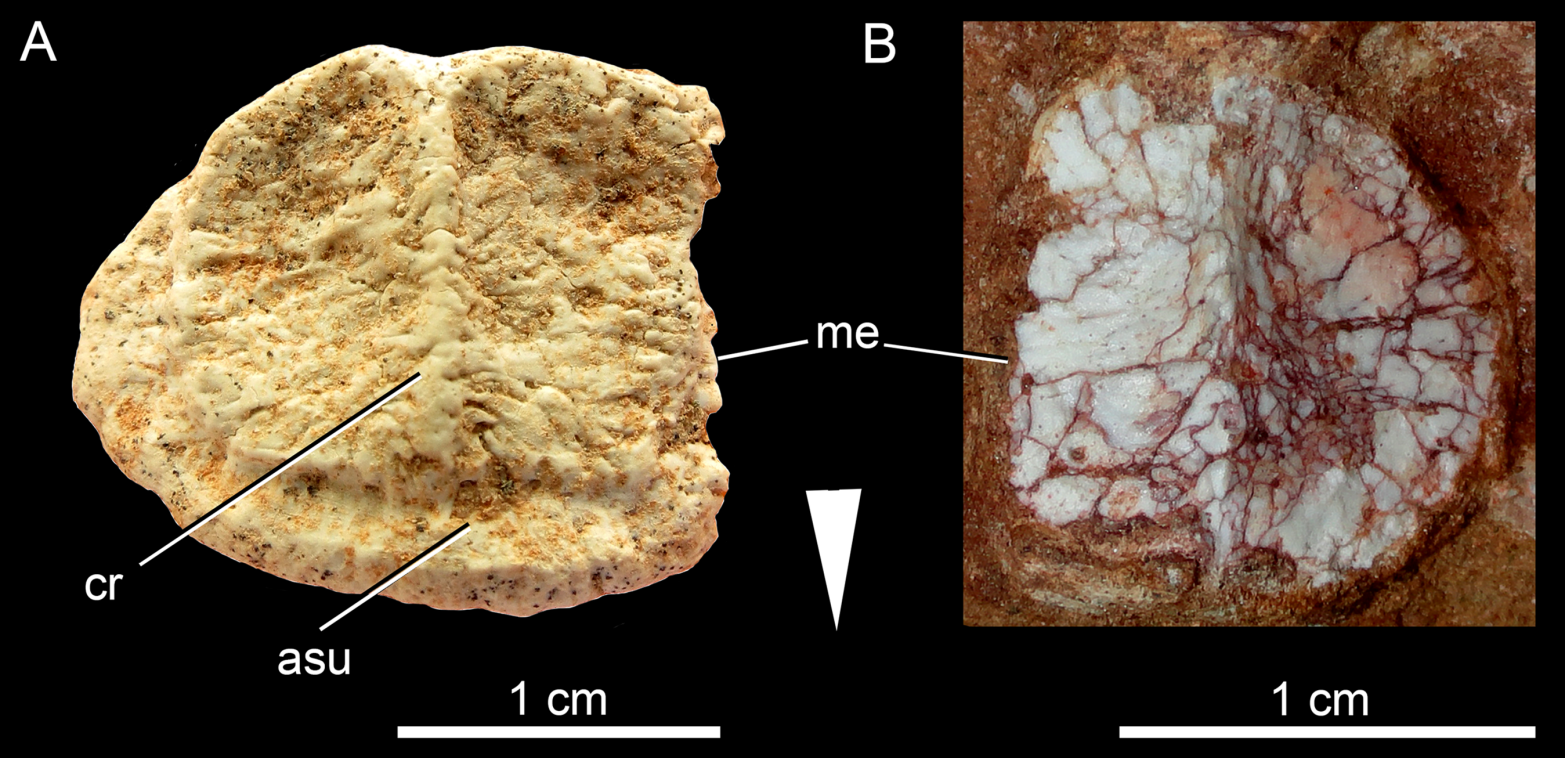
Comparison of a caudal osteoderm in *Caipirasuchus* species in dorsal view. Right osteoderm (A) of *C. montealtensis* (MPMA 68-0003/12) and left osteoderm (B) of *C. mineirus* (CPPLIP 1463). Abbreviations: asu, articular surface for anterior osteoderm; cr, longitudinal crest; me, medial edge. The arrow indicates the anterior side of the osteoderms.

*Caipirasuchus mineirus* has a paramedian shield formed by two parallel rows of osteoderms. In *C. montealtensis* (MPMA 68-0003/12; Iori, Marinho & Carvalho, 2016) (Fig. 24A) there are also two rows of paramedian osteoderms, in which each osteoderm is articulated with its transversal counterpart and overlaps with the anterior and posterior osteorderms (Iori, Marinho & Carvalho, 2016: fig. 6). In *C. mineirus*, most osteoderms have a medial rugose area to suture with its counterpart (Fig. 24B), similar to *C. montealtensis*. There is no positive evidence to support the idea that osteoderms of each longitudinal row articulate by means of overlapping surfaces, due to the absence of these surfaces. All osteoderms are weakly ornamented, bearing a main longitudinal crest, and several small nutritious foramina.

Presacral osteoderms are D-shaped, with a the flat edge constituting the medial side and the convex edge the lateral one (Fig. 23). They are about two times anteroposteriorly larger than transversely broad; therefore, they are more elongated than caudal osteoderms. Near the medial straight edge there is a subtle longitudinal crest, that defines the medial and the lateral surfaces. In presacral osteoderms, the medial surface is considerably smaller than the lateral one. The crest slopes gradually at both anterior and posterior edges of the osteoderm (Fig. 23E).

Sacral and proximal caudal osteoderms are subsquared in shape, with a medial straight edge and anterior, posterior, and lateral edges defining an rounded contor. Different from the elongated presacral ones, these osteoderms are just slightly anteroposteriorly longer than transversely broad (Fig. 23F). The longitudinal crests in these osteoderms have a more medial position and are slightly higher than in the presacral ones. Toward the middle and distal portions of the caudal region, the osteoderms become smaller, although still keeping a squared shape.

### Scapula

**Figure 25:**
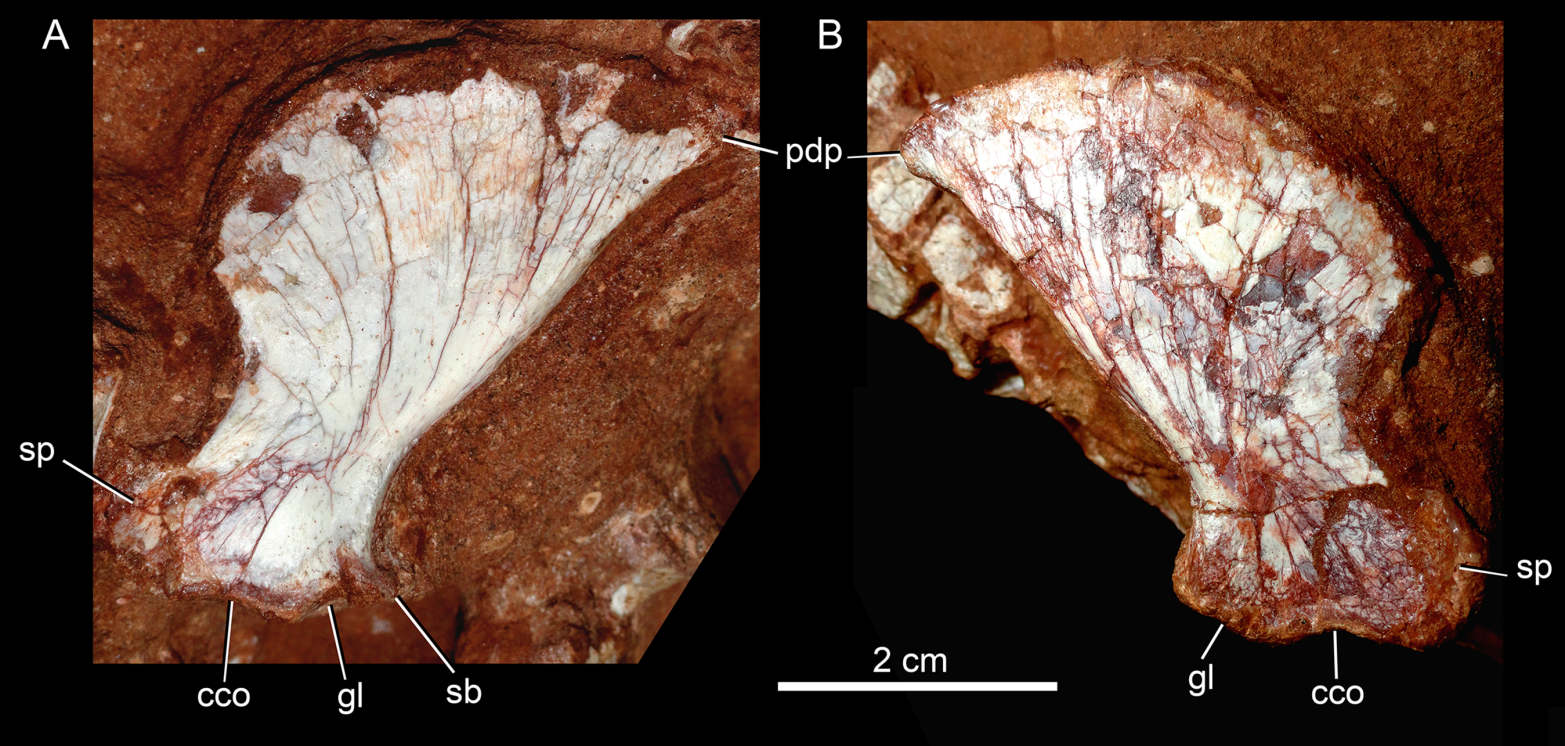
Scapulae of *Caipirasuchus mineirus*, CPPLIP 1463. Right (A) and left (B) scapula in medial view. Abbreviations: cco, contact surface for coracoid; gl, glenoid; pdp, posterodorsal projection; sb, scapular buttress; sp, scapular prominence.

Both scapulae are preserved and are laying on their lateral surfaces, hampering the observation of external features. The scapular blade is dorsally well-developed, with the medial surface almost flat and slightly convex at its posterodorsal portion (Fig. 25). The dorsal profile of the scapular blade is strongly convex, with the anterodorsal point below the midline of the dorsoventral height of the scapula. The dorsal edge of the scapular blade is less convex in *Y. boliviensis* (Leardi et al., 2015b) than in *C. mineirus*, producing a larger angle. The posterodorsal projection is acute (Fig. 25), differing from the more rounded angle of *Y. boliviensis*. The anterior profile is also notched, with an square-shaped scapular prominence (=acromial process). The acromial region is dorsoventrally shorter than in *Y. boliviensis* and *Simosuchus clarki*, but larger than in *N. terrestris* (Pol, 2005). The contact surface for the coracoid is relatively large and slightly concave. Posterior to this point, the glenoid is gently concave and posteriorly bordered by the scapular buttress, forming a conspicuous crest on the medial side of the scapula. The glenoid facet of the scapula is ventrally oriented, as in most notosuchians (Leardi et al., 2015b), with an anterior bulge to the glenoid. The scapular buttress projects posteroventrally, defining a concave posterior edge of the scapular blade (Fig. 25).

### Humerus

**Figure 26:**
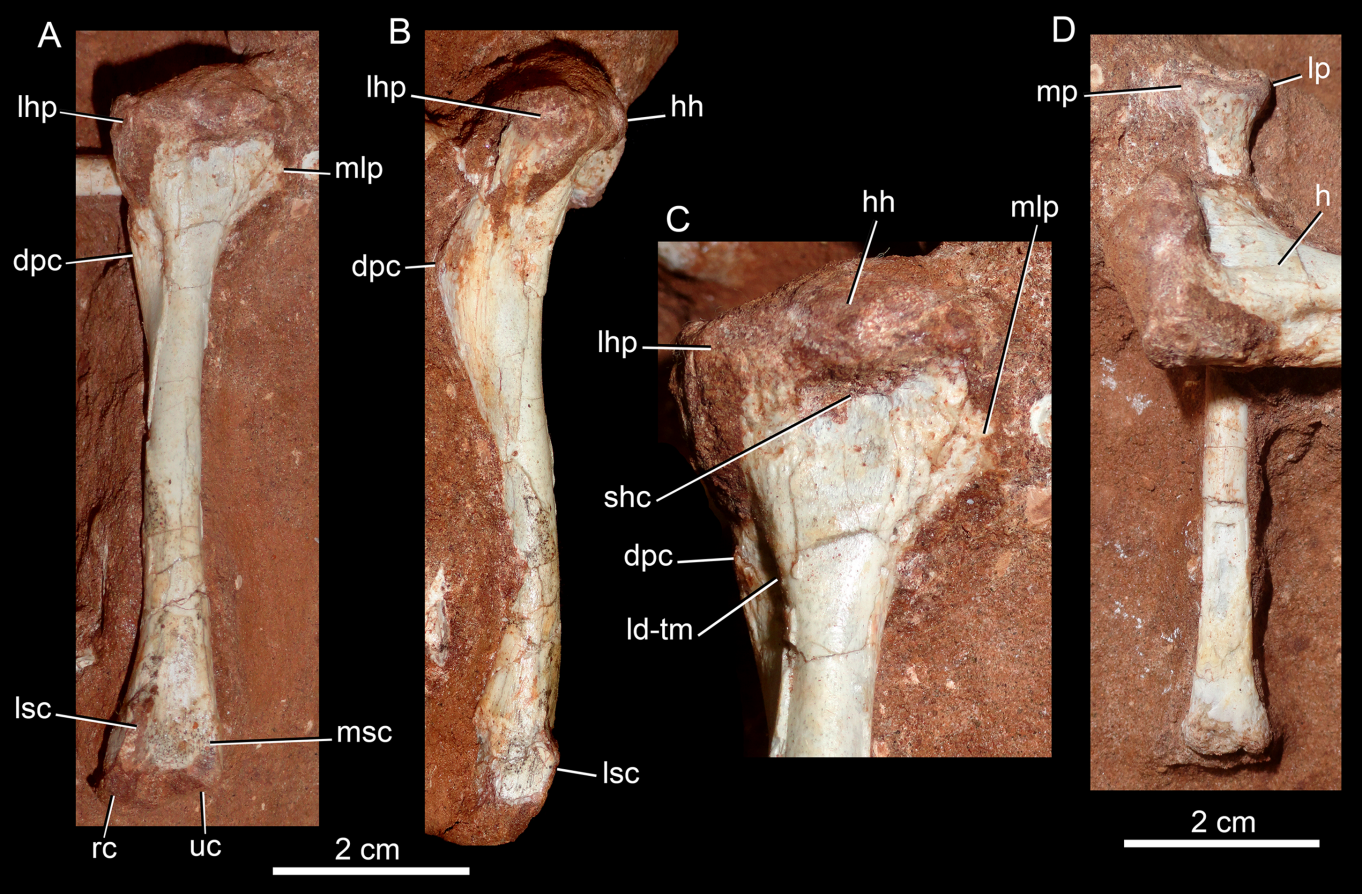
Humerus and radius of *Caipirasuchus mineirus*, CPPLIP 1463. Left humerus in posterior (A) and lateral (B), with details of its proximal its proximal half in posterior view (C), and left radius in anterior view (D). Abbreviations: dpc, deltopectoral crest; h, humerus; hh, humeral head; ld-tm, fossa for the common insertion of the M. latissimus dorsi and M. teres major; lsc, lateral supracondylar crest; lhp, lateral humeral process; lp, lateral process; mlp, medial humeral process; mp, medial process; msc, medial supracondylar crest; rc, radial condyle; shc, depression for the M. scapulohumeralis caudalis; uc, ulnar condyle.

The left humerus is almost complete, missing only part of the distal articular surface (Fig. 26), although its anterior surface is still within the rock matrix. It is an slender bone, with a long shaft and relatively narrow proximal and distal ends (Fig. 26A). In lateral view, the bone has a slight sigmoidal shape (Fig. 26B). The proximal end is almost 1.6 times lateromedially wider than the preserved distal end. The humeral head is longer lateromedially than anteroposteriorly with an anteroposterior convex surface. Distal to the humeral head there is a thin medial tuberosity that produces a gently concave medial outline of the proximal portion of the humerus. Distal to the posterior border of the humeral head, there is a conspicuous and well-delimited depression for the M. scapulohumeralis caudalis (Meers, 2003). Lateral to this depression, there is a rounded crest that descends from the humeral head on the posterolateral edge of the bone and slopes the shaft gradually. A shallow and oval depression is defined between this crest and the deltopectoral crest, for the insertion of the M. latissimus and M. teres major (Fig. 2; Meers, 2003). The deltopectoral crest is well developed and anteriorly projected (Fig. 26B). The lateral surface of this crest is gently convex, sloping the shaft gradually. It is restricted to the proximal half of the bone, with its apex at its midheight, giving an symmetric aspect to the deltopectoral crest in lateral view. Below the level of the deltopectoral crest, the humeral shaft narrows considerably, with almost parallel lateral and medial edges and a circular cross-section (Fig. 26A). Toward the distal end, the lateral, and medial edges slightly diverge from each other, but most of the condyle is not preserved. In this portion, the lateral supracondylar crest is sharp and high, whereas the medial one is shallow, represented by an acute edge formed by the posterior and medial surfaces of the distal end. Both lateral and medial surfaces are flat.

The general morphology of the humerus is similar to that of *Caipirasuchus montealtensis* (Iori, Marinho & Carvalho, 2016) and does not differ significantly from that of *Y. boliviensis* (Leardi et al., 2015b), whereas in *Armadillosuchus*, the humerus is stouter (UFRJ DG 303-R).

### Radius

The left radius is placed perpendicular to and below the humerus, laying on its posterior surface. The radius is also a slender element, with about 80% of the length of the humerus (Fig. 26D). The proximal end is mediolaterally asymmetrical, with the medial process slightly more projected than the lateral one. The long shaft has almost parallel lateral and medial edges. The distal end is transversely shorter than the proximal one (Fig. 26D). The radiale articular surface is small and all the distal articular surface is at the same plane, perpendicular to the major axis of the bone. Although preserved in the *Caipisaruschus* sp. specimen MPMA 68-0003/12 (Iori, Marinho & Carvalho, 2016), the radius was not described. This element is similar to that described for *Y. boliviensis*, although in the latter taxon the medial and lateral proximal processes are more developed (Leardi et al., 2015b).

### Ilium

**Figure 27:**
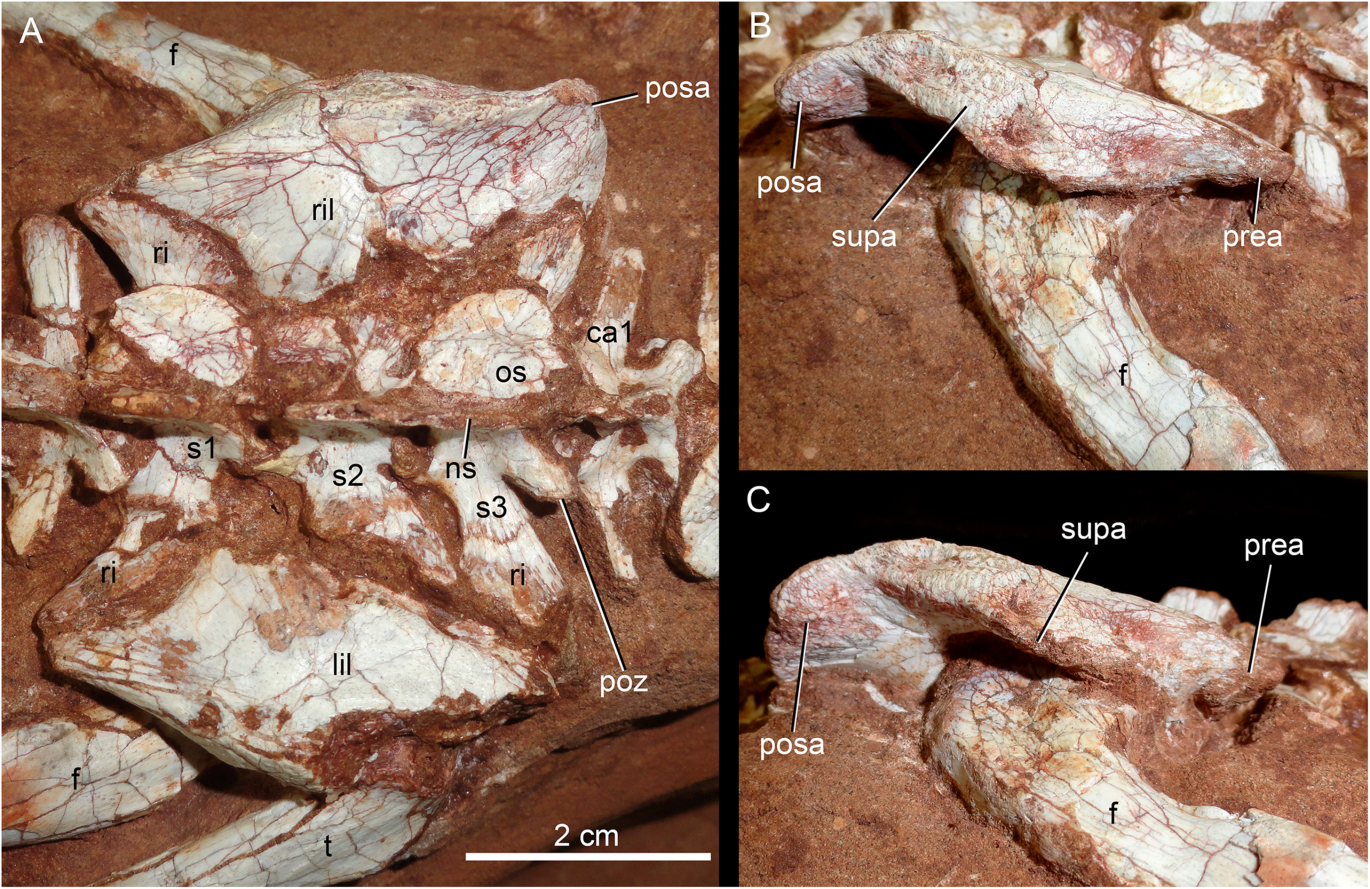
Ilia of *Caipirasuchus mineirus*, CPPLIP 1463. Ilia and sacrum in dorsal view (A) and detail of right ilium in laterodorsal (B) and lateral (C) views. Abbreviations: ca1, first caudal vertebra; f, femur; lil, left ilium; ns, neural spine; os, osteoderm; posa, postacetabular process; poz, postzygapophysis; prea, preacetabular process; ri, rib; ril, right ilium; supa, supracetabular crest; s, sacral vertebra; t, tibia.

Both ilia are preserved and articulated to the sacrum (Fig. 27), with both femoral heads positioned at the acetabulum. For this reason, most of the ventral and acetabular portions of the ilium can not be accessed. The ilium has a short preacetabular process (Fig. 27C), as in most mesoeucrocodylians (Pol, 2005; Pol et al., 2012). Its external surface is triangular-shaped and ventrolaterally projected. The supraacetabular crest is transversely wide and deflected (Figs. 27B and 27C). Its maximum width is at the middle of the acetabular area and it tapers anteriorly and posteriorly. This crest and the dorsal surface of ilium are almost undifferentiated. It has a flat surface with a rugose texture and two conspicuous depressions. The lateral orientation and development of both the supracetabular crest and of the dorsal edge of the ilium produce a transversely wide dorsal surface, between the dorsolateral edge of the ilium and the area for contact of the transverse processes and the ribs of the sacral vertebrae (Fig. 27A). Moreover, the acetabular area faces almost ventrally, permitting a more upright position of the femur, as interpreted for other notosuchians (Pol, 2005; Riff & Kellner, 2011; Iori, Marinho & Carvalho, 2016). The robust supracetabular crest is similar to other notosuchians, such as *N. terrestris* (Pol, 2005) and *Mariliasuchus amarali* (Nobre & Carvalho, 2013). This structure is even more developed in sebecosuchians, such as *B. albertoi* (Nascimento & Zaher, 2010), *Stratiotosuchus maxhechti* (Riff & Kellner, 2011) and *Sebecus icaeorhinus* (Pol et al., 2012). The posteroacetabular process is larger than the preacetabular process, and forms an almost horizontal projection that bends posteroventrally (Figs. 27B and 27C), as in several notosuchians, such as *Uruguaysuchus aznarezi* (Rusconi, 1933), *N. terrestris* (Pol, 2005), and *Chimaerasuchus paradoxus* (Wu & Sues, 1996). Most of the transverse process of the third sacral vertebra attaches at this point.

### Femur

**Figure 28:**
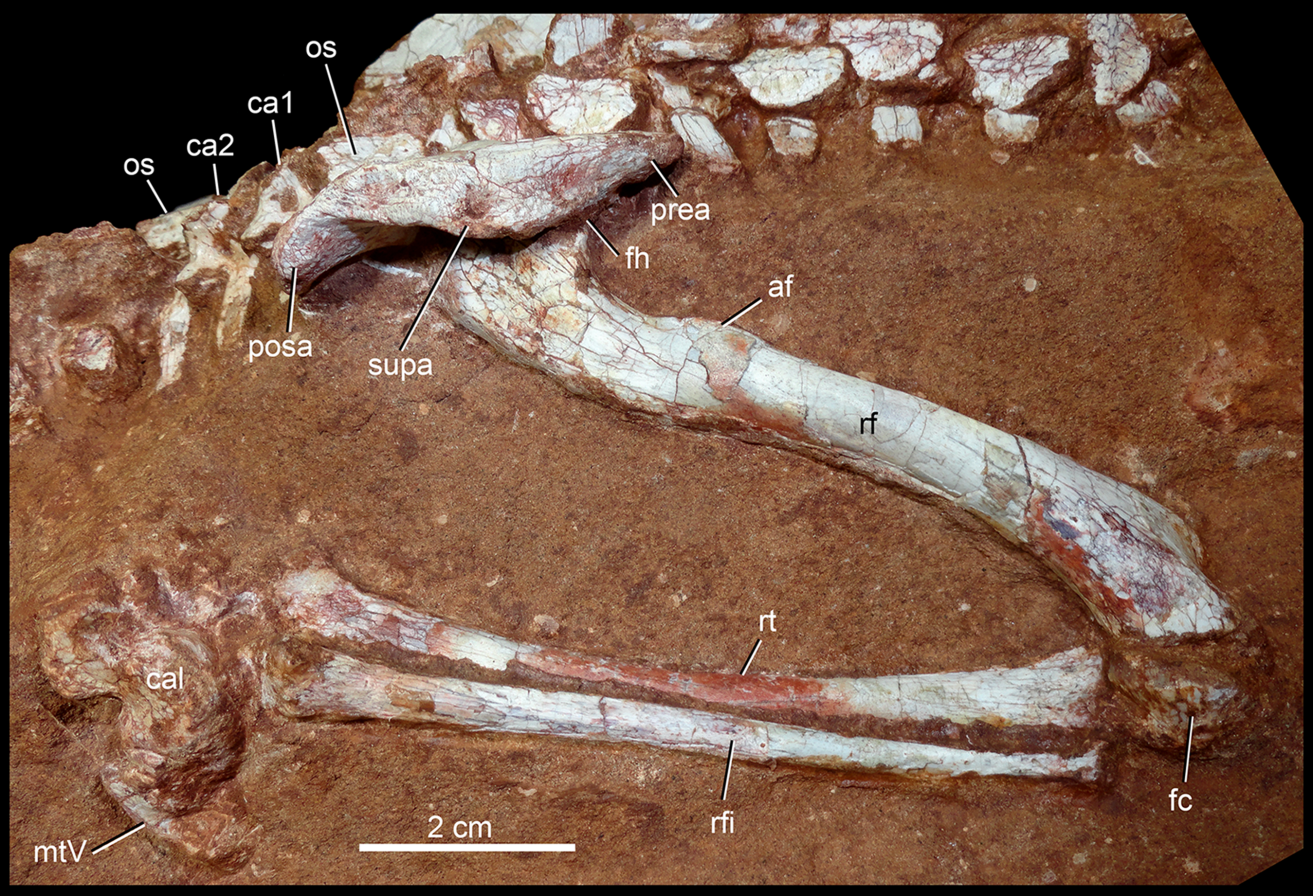
Right hindlimb of *Caipirasuchus mineirus*, CPPLIP 1463. Flexed right hindlimb articulated to the ilium in laterodorsal view. Abbreviations: af, anterior flange; cal, calcaneum; c1–2, caudal vertebra 1 and 2; fc, fibular condyle; fh, femoral head; il, ilium; rf, right femur; rfi, right fibula; rt, right tibia; mtV, metatarsal V; os, osteoderm; posa, postacetabular process; prea, preacetabular process; supa, supraacetabular crest.

**Figure 29:**
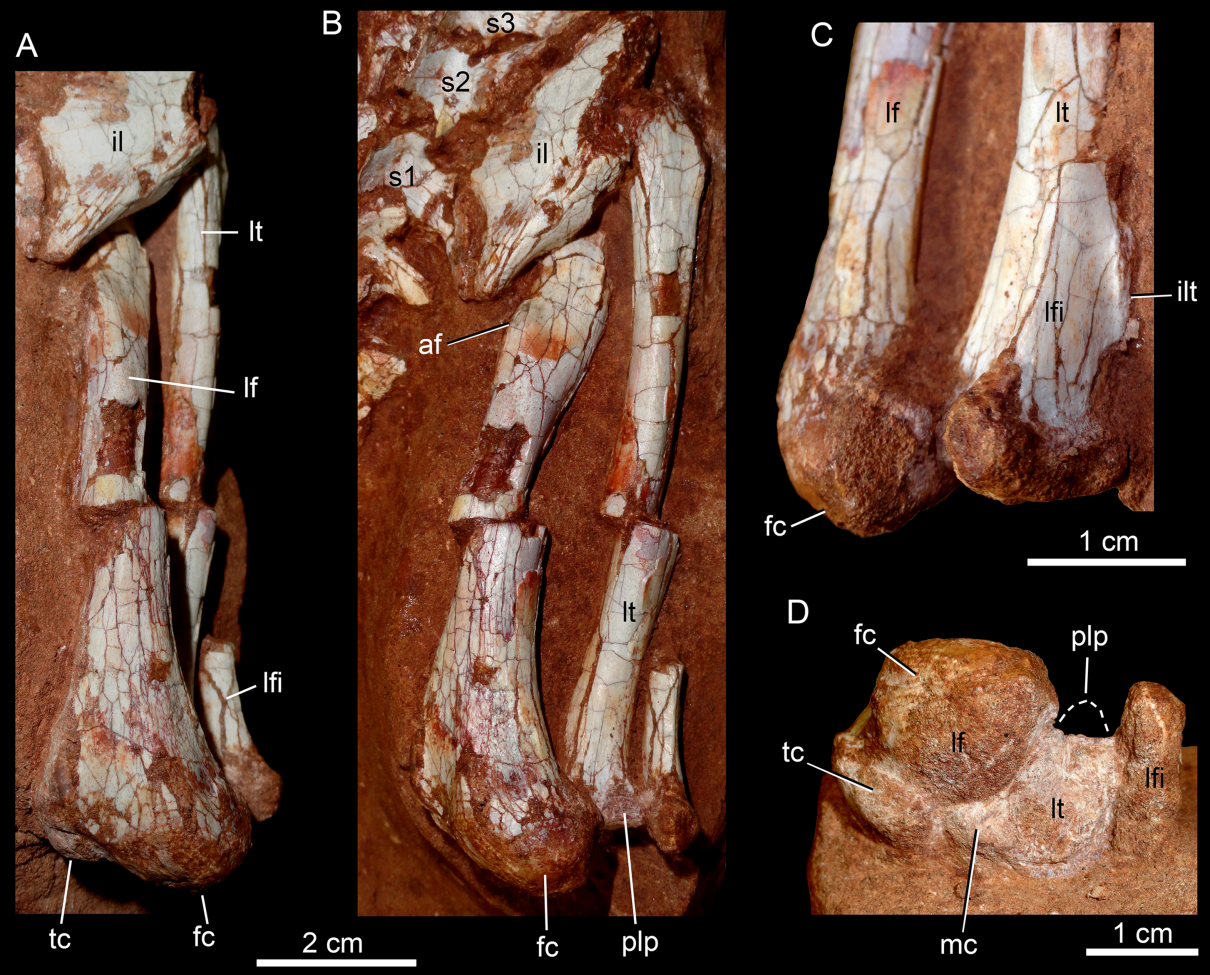
Left hindlimb of *Caipirasuchus mineirus* CPPLIP 1463. (A) Flexed left hindlimb, articulated to the ilium with the femur in anterior view. (B) Flexed left hindlimb, articulated to the ilium with the femur in anterolateral view. (C) Details of the knee in lateral view. (D) Femur in distal view in articulation with tibia and fibula in proximal view. Abbreviations: af, anterior flange; fc, fibular condyle; i, ilium; ilt, iliofibularis trochanter; mc, medial condyle; lf, left femur; lfi, left fibula; lt, left tibia; s1–3, sacral vertebra 1–3; plp, posterolateral process of the tibia; tb, tibial condyle.

Both femora are preserved, resting on their posterior surface and with the femoral head articulated to the pelvis, which prevents the observation of some features. The femur is slender as the other long bones of *C. mineirus*. It is about 30% proximodistally longer than the humerus. It is almost straight in anterior view, and slightly sigmoidal in lateral view (Fig. 28), without the strong sigmoidal aspect of most crocodyliforms (Mook, 1932). Although the femoral head is not observed, it is clear the absence of a distinctive neck. The poorly developed greater trochanter emerges as a rounded ridge on the anterolateral corner of the proximal portion of the bone (Fig. 28). Clear muscle scars are not evident. The anteromedial flange, located below the level of the greater trochanter, marks the insertion for the M. puboischiofemoralis internus (Leardi et al., 2015b) . This flange projects anteromedially as a sharp crest, and produces a marked convex profile between itself and the femoral head base. In *Y. boliviensis,* the anteromedial flange is very reduced to absent (Leardi et al., 2015b), whereas in taxa such as *Simosuchus clarki*, it is well-developed (Sertich & Groenke, 2010). The shaft diverges its lateral and medial edges along the extension of its distal-most portion. In anterior view, the supracondylar groove is flat to slightly concave (Fig. 29A). The fibular condyle develops more distally than the tibial one, and is conspicuously larger in distal view (Fig. 29D), as in most crocodyliforms (Mook, 1932; Sertich & Groenke, 2010).

The femur of *C. mineirus* resembles that of *Y. boliviensis*, but in the latter taxon the anteromedial flange and the width of the distal end are less developed (Leardi et al., 2015b). The femur in other *Caipirasuchus* species is unknown or too fragmentary (Iori, Marinho & Carvalho, 2016).

### Tibia

Both tibiae are preserved, but with limited information available on their distal ends. The tibia is slender and slightly proximodistally shorter than the femur (Figs. 28 and 29). Clear muscle scars are not seen on the tibia. The proximal facet is subtriangular with a rounded medial process (Fig. 29D). This shape is different to the roughly rhomboidal aspect of *Yacarenani* (Leardi et al., 2015b), more similar to the triangular-shape present in *Araripesuchus tsangatsangana* (Turner, 2006) and some sebecosuchians (e.g., *Sebecus icaeorhinus*; Pol et al., 2012). The broken rounded base of the posterolateral process is observed in posterior view. The articular surface for the fibula is slightly concave. In the right side, the tibia is slightly shifted from its natural position. Although shifted, the shaft seems to be slightly arched laterally as in most non-neosuchian mesoeucrocodylians (Nascimento & Zaher, 2010; Leardi et al., 2015b; Pol et al., 2012). The shaft is subcircular in cross-section, with an smaller diameter than the femoral shaft. The distal end is slightly expanded transversely, with the medial condyle larger and more distally positioned than the lateral condyle, as seen in the left tibia (Fig. 29), as occurs in most crocodyliforms.

### Fibula

The right fibula is articulated with the tibia, whereas only a small proximal portion of the left fibula is preserved, also in articulation (Figs. 28 and 29). The fibula is also a long and slender bone, slightly proximodistally shorter than the tibia. It is lateromedially compressed, especially in its proximal half. The fragmentary left fibula exhibits an anteroposterior elongate fibular head, with a conspicuous posterior projection. On the anterolateral corner of the bone there is a marked crest, the iliofibularis trochanter, in which a long lateral ligament is inserted (Turner, 2006). Posterior to this crest, the bone surface is flat. The left fibula preserves its distal end, which is is slightly anteroposteriorly wider than the shaft, with an almost flat distal end (Fig. 29D). As in other notosuchians, such as *Yacarerani*, *Araripesuchus tsangatsangana*, and baurusuchids (Turner, 2006; Leardi et al., 2015b; Riff & Kellner, 2011), the distal end lacks a medial hook.

### Tarsals and metatarsal

**Figure 30:**
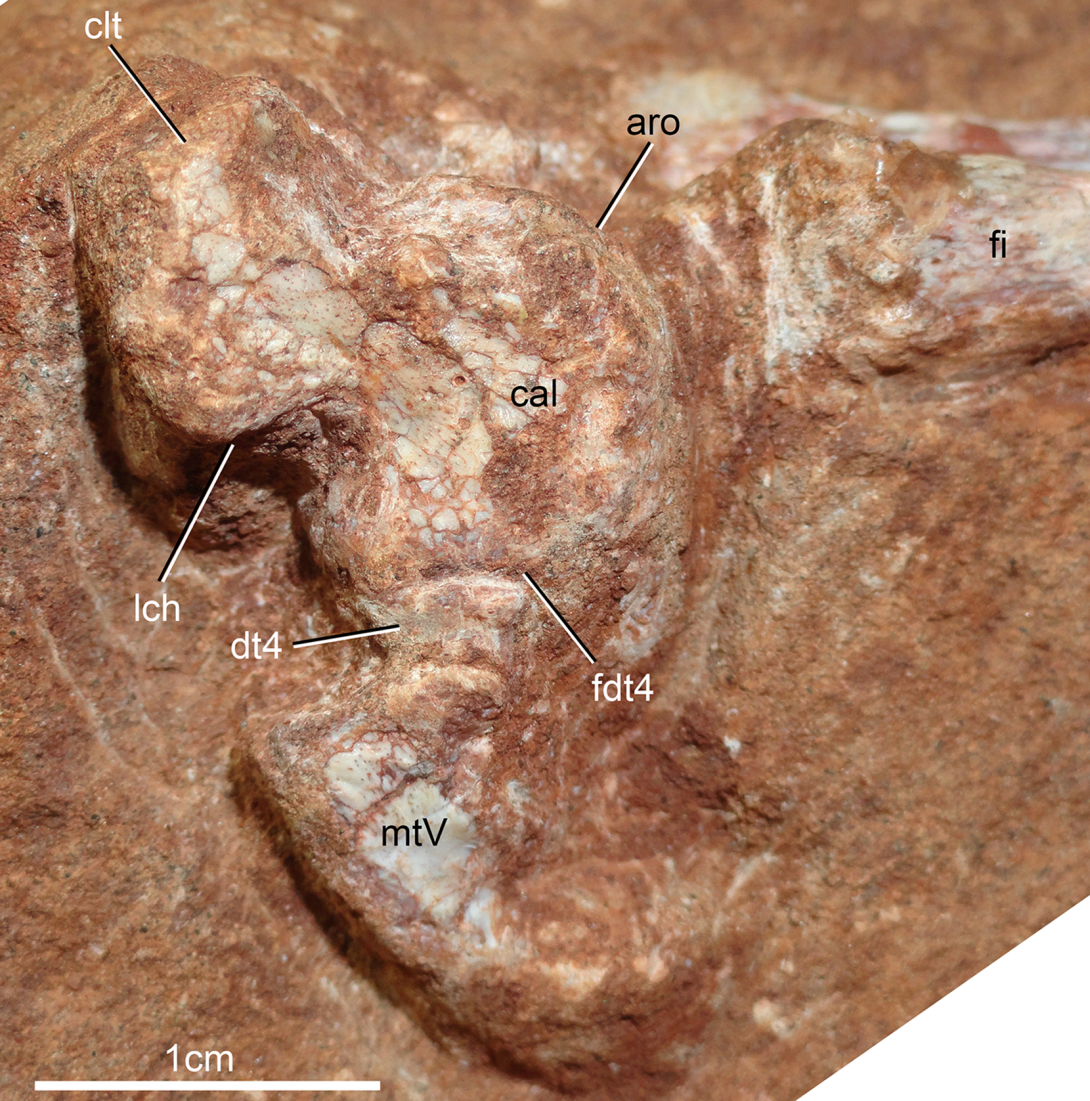
Right tarsals (calcaneum and distal tarsal 4) and metatarsal V of *Caipirasuchus mineirus*, CPPLIP 1463 in lateral view. Abbreviations: aro, anterior roller (condyle); cal, calcaneum; clt, calcanear tuber; dt4, distal tarsal 4; fi, fibula; lch, lateral channel; fdt4, facet for distal tarsal 4; mtV, metatarsal V.

The right calcaneum is in articulation with the fibula and the distal tarsal 4, which in turn is in articulation with the metatarsal V (Fig. 30). The calcaneum is exposed in lateral view, and bears two main structures: the rounded anterior roller (or condyle) and the posterior calcaneal tuber. The roller is anteriorly convex, with a lateral elevated rim that limits a posterolateral fossa (Fig. 30). The ventral facet for the distal tarsal 4 is slightly concave, differing from the straight to slightly convex surface seen in *Y. boliviensis* (Leardi et al., 2015b) and *Mariliasuchus amarali* (Nobre & Carvalho, 2013). The calcaneal tuber seems to be complete and is lower than the height of the anterior roller, being more developed dorsoventrally in *C. montealtensis* (Iori, Marinho & Carvalho, 2016), *Y. boliviensis* (Leardi et al., 2015b), and *Mariliasuchus amarali* (Nobre & Carvalho, 2013). The lateral channel (Sertich & Groenke, 2010) is deep and develops from the posteroventral border of the calcaneal tuber to the anteroventral portion of the body. In general aspect, although poorly preserved, the anterior roller of *Caipirasuchus mineirus* looks more robust than in *C. montealtensis* and *Y. boliviensis*, whereas the opposite is seen for the calcaneal tuber. Also, the body of the calcaneum, between the anterior roller and calcaneal tuber, is more elongated in *Y. boliviensis* than in *C. mineirus*.

The information on the distal tarsal 4 is limited. It is small, with an inverted trapezoidal shape in lateral view (Fig. 30). The element identified as metatarsal V is a reduced triangular-shaped bone in lateral view, with the anterior surface slightly concave (Fig. 30), although poorly preserved. This element was no yet described for any “advanced notosuchian,” although present (but not usually preserved) in most crocodylomorphs (Romer, 1956).

## Discussion

### Taxonomy of *C. mineirus*

Some of the features present in *C. mineirus* were previously used for the diagnoses of the other *Caipirasuchus* species (Pol et al., 2014). Nonetheless, the holotype of *C. mineirus* cannot be referred to any of these species, due to the presence/absence of other specific traits. The holotype of *C. mineirus* shows several features that are indicative of juvenile or subadult stage, such as: relatively small size of the specimen, large orbits, poorly ornamental skull bones, unfused neural central sutures between the arch and the centrum, and lack of fussion between diapophyses and their respective rib in sacral vertebrae, between sacral ribs and ilium. However, it is not an extremely small individual in comparison to the ones known for other *Caipirasuchus* species, all sharing a similar skull shape (e.g., very young indiviuals of alligators exhibit considerably changes with respect to subadult/adult individuals; Iordansky, 1973). Consequently, we consider that the holotype of *C. mineirus* has a combination of characters that is unique and diagnostic.

**Figure 31:**
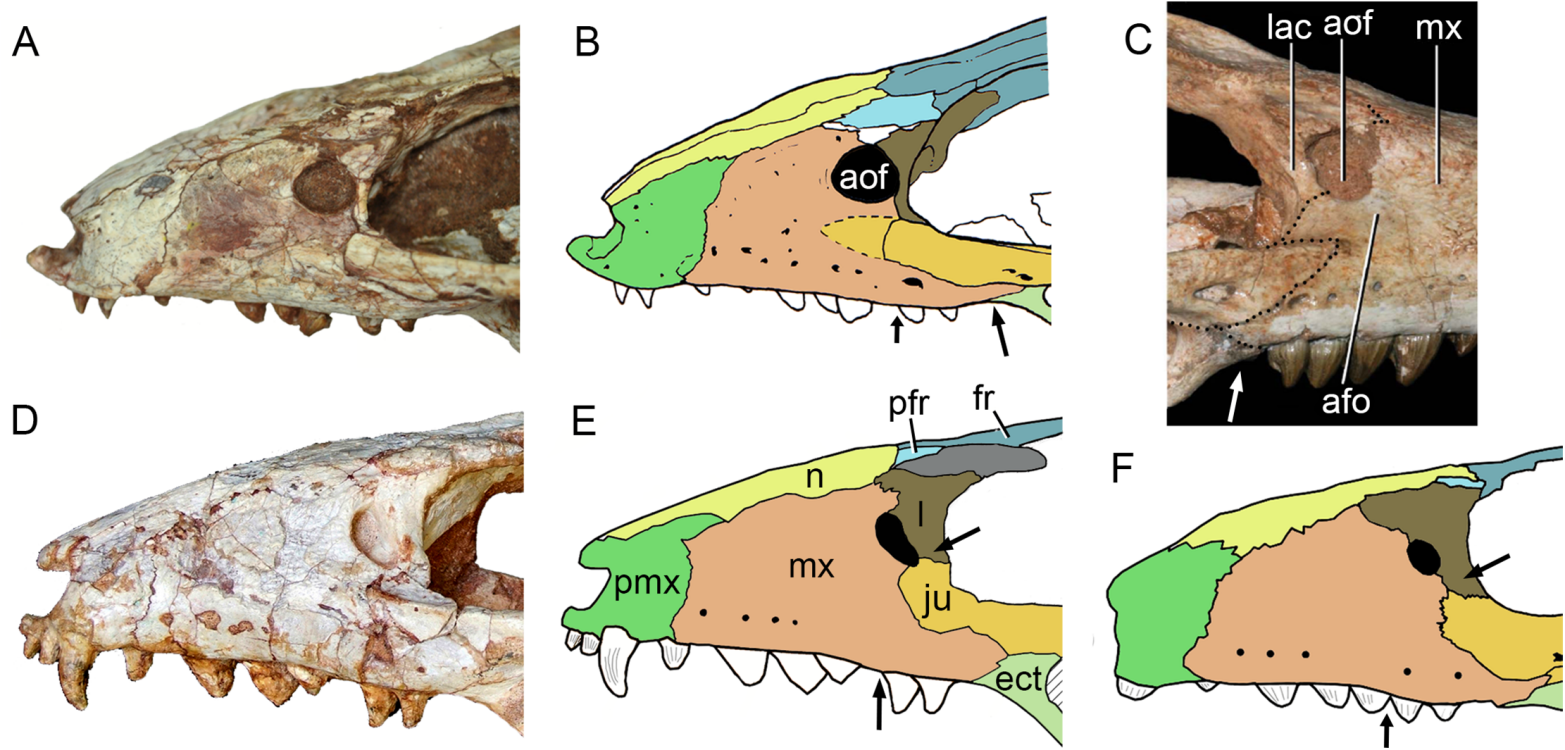
Comparison of the snout in *Caipirasuchus* spp. in lateral view. Photograph of *Caipirasuchus mineirus* (holotype, CPPLIP 1463) (A) with schematic drawing (B). Photograph of *Caipirasuchus stenognathus* (holotype, MZSP-PV 139) (C). Photograph of *Caipirasuchus paulistanus* (holotype, MPMA 67-0001/00) (D) with schematic drawing (E). Schematic drawing of *Caipirasuchus montealtensis* (holotype, MPMA 15-0001/9) (F). Arrows indicate main differences in this portion of the skull. B was modified from Pol et al. (2014). Abbreviations: afo, antorbital fossa; aof, antorbital fenestra; ect, ectopterygoid; fr, frontal; ju, jugal; lac, lacrimal; mx, maxilla; n, nasal; pfr, prefrontal; pmx, premaxilla.

**Figure 32:**
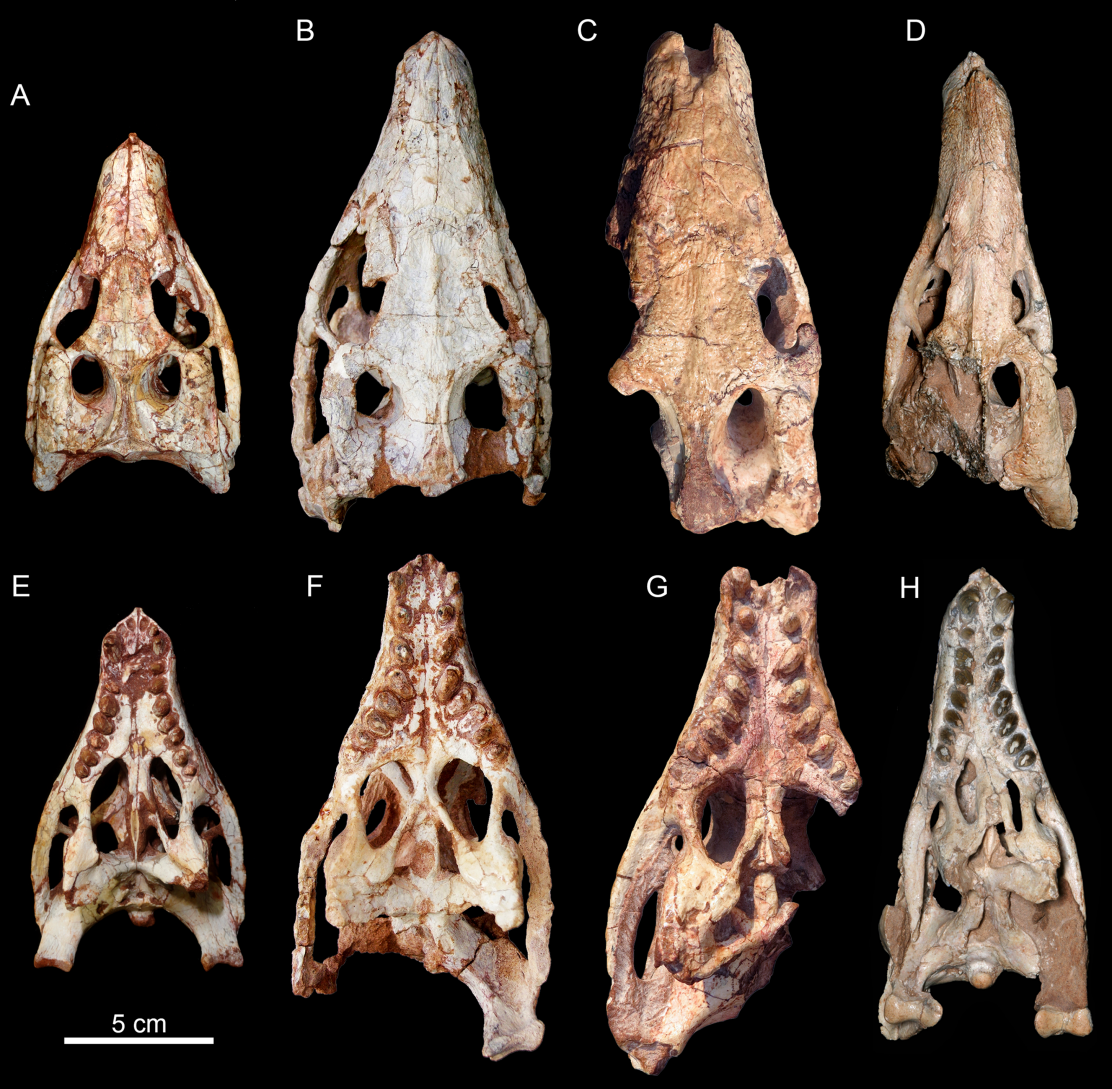
Comparisons among *Caipirasuchus* species. (A and E) Skull of *Caipirasuchus mineirus* (Holotype CPPLIP 1463). (B and F) *Caipirasuchus paulistanus* (Holotype MPMA 67-0001/00). (C and G) *Caipirasuchus montealtensis* (Holotype MPMA 15-0001/9). (D and H) *Caipirasuchus stenognathus* (Holotype MZSP-PV 139). (A–D) are in dorsal view and (E–H) in ventral view. (D and H) were modified from Pol et al. (2014).

**Figure 33:**
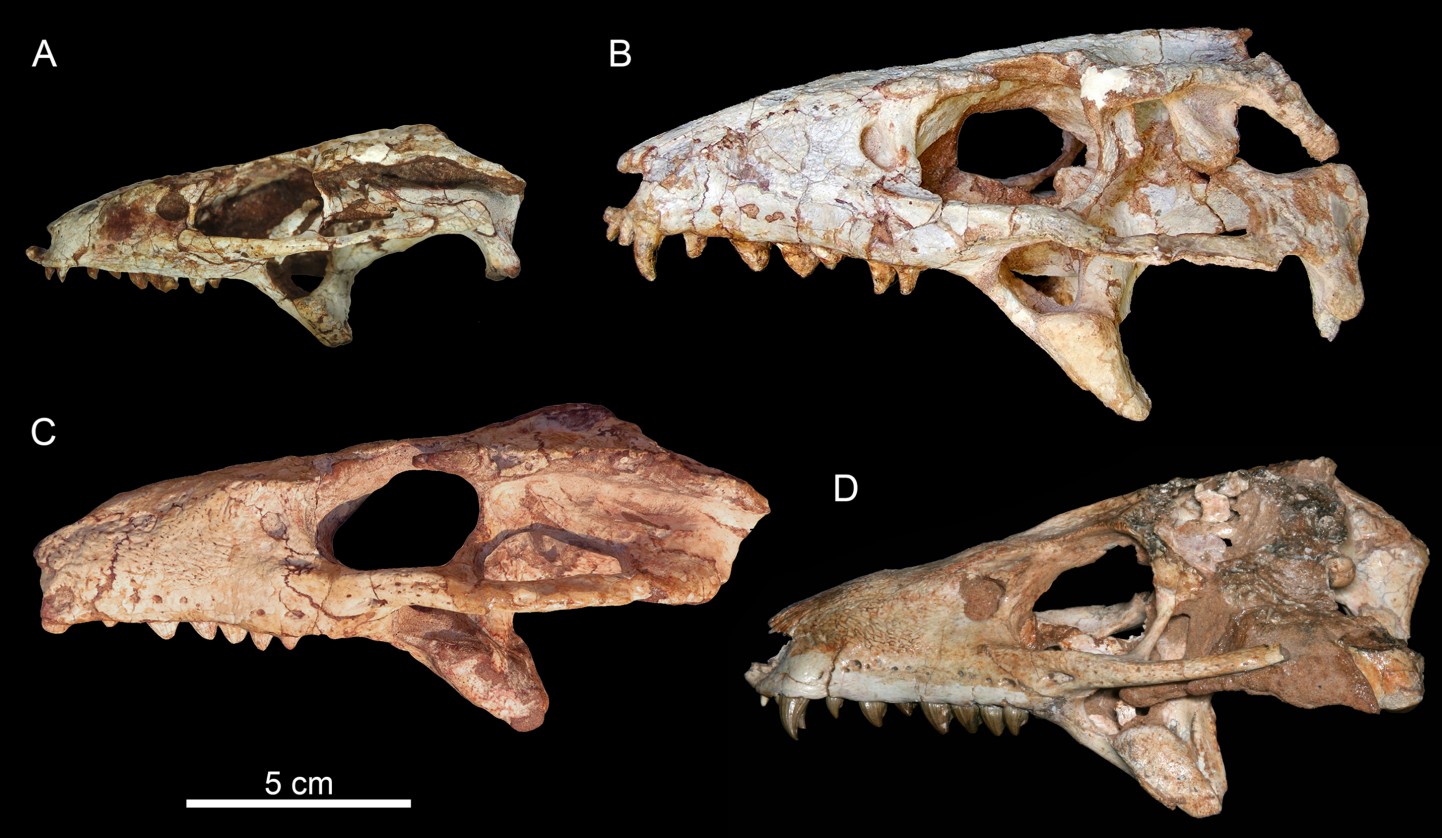
Comparisons among *Caipirasuchus* species. (A) skull of *Caipirasuchus mineirus* (Holotype CPPLIP 1463). (B) *Caipirasuchus paulistanus* (Holotype MPMA 67-0001/00). (C) *Caipirasuchus montealtensis* (Holotype MPMA 15-0001/90; inverted image). (D) *Caipirasuchus stenognathus* (Holotype MZSP-PV 139). All in lateral view. (D) was modified from Pol et al. (2014).

**Figure 34:**
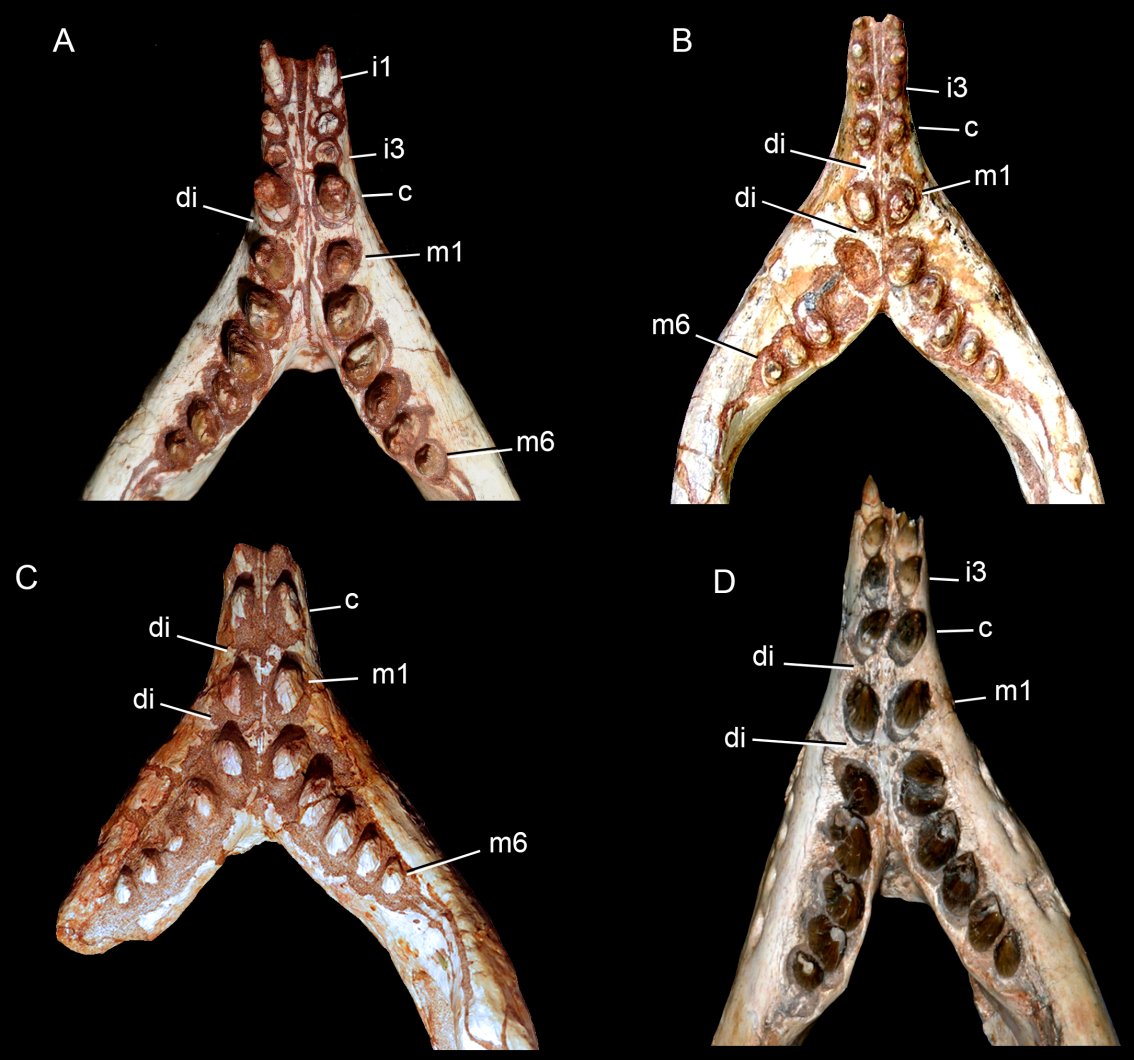
Comparisons among *Caipirasuchus* species. (A) Lower dentition of *Caipirasuchus mineirus* (Holotype CPPLIP 1463). (B) *Caipirasuchus paulistanus* (Holotype MPMA 67-0001/00). (C) *Caipirasuchus montealtensis* (Holotype MPMA 15-0001/90). (D) *Caipirasuchus stenognathus* (Holotype MZSP-PV 139). All in occlusal view. (D) was modified from Pol et al. (2014). Not to scale. Abbreviations: c, caniniform; di, diastema; i, incisiviform; m, molariform.

*Caipirasuchus mineirus* differs from *C. paulistanus* by having a rounded antorbital fenestra, without a distinct posteroventral projection (Fig. 31); a conspicuous longitudinal groove on choanal septum; the lack of anterodorsal process of the jugal; the ectopterygoid forming part of posterior edge of the last alveolus; and by lacking the well-developed diastema anterior and posterior to fifth dentary tooth (Figs. 32–34).

The presence of a posteriorly concave semicircular ridge on the posterior margin of the nasals, associated to a transversal groove between the nasals and the fused frontal, was considered as an autapomorphy of *C. paulistanus* (Pol et al., 2014). This condition is also present in *C. mineirus*; therefore, this feature should be excluded as autapomorphic in the emended diagnosis of *C. paulistanus* (Pol et al., 2014). Consequently, the distinctive posteroventral projection of the antorbital fenestra of *C. paulistanus* (Fig. 31) remains as its only autapomorphy (Pol et al., 2014).

The frontal sagittal crest reaching the anterior end of the bone was considered diagnostic for *C. paulistanus* (since in *C. montealtensis* and *C. stenognathus* the crest is restricted to the two-third posterior portions of the bone) (Pol et al., 2014). In *C. mineirus*, the crest is developed all along the bone, making this a condition shared with *C. paulistanus* (Fig. 32). Consequently, it is no longer exclusive of the latter species.

Pol et al. (2014) mentioned that the anterior end of the palatine, at the contact with the palatal process of the maxilla, is more flared in *C. paulistanus* than in the other *Caipirasuchus* species (*C. stenognathus* and *C. montealtensis*). CPPLIP 1463 has a similar configuration to that of *C. paulistanus*. Furthermore, we agree with Pol et al. (2014), in that the condition seen in *C. montealtensis* (MPMA 15-0001/90) and C. *stenognathus* (Pol et al., 2014) are different from that of *C. paulistanus*. In *C. montealtensis*, the right palatal suture between maxilla and palatine is flared as in *C. paulistanus*. In *C. stenognathus* there is also a flared contact, although, the lateral compression of the skull has deformed this portion of the skull (Fig. 32). For this reason, we do not considered this feature as diagnostic of *C. paulistanus*.

In addition to the combination of features listed as diagnostic for *C. mineirus*, this species differs from *C. montealtensis* by lacking a single, rounded and deep parachoanal fossa on the pterygoid flanges (De Andrade & Bertini, 2008a; Iori et al., 2013; Pol et al., 2014) and by having the ectopterygoid forming part of posterior edge of the last alveolus (Fig. 32).

*Caipirasuchus mineirus* differs from *C. stenognathus* by having a thin descending process of the lacrimal which contacts the jugal and excludes the maxilla from the orbital rim (see also below) (Figs. 31 and 33); the fourth dentary tooth is the largest of the series; and by lacking a diastema between the fifth dentary tooth and the remaining ones (Fig. 34).

For Pol et al. (2014), the maxilla forms part of the orbital rim, precluding a jugal-lacrimal contact, and it would be a diagnostic character for *C. stenognathus*. On the left side of CPPLIP 1463, the lacrimal has a thin descending process that contacts the anterior process of the jugal. This contact is also observed in *C. paulistanus* and *C. montealtensis*. However, the condition is quite different in these two taxa, with the descending process of the lacrimal dorsoventrally shorter and more robust, and a well-developed anterodorsal process of the jugal, features not present in *C. mineirus*. On the right side of the orbital region of *C. mineirus*, however, the descending process of the lacrimal is long and almost reaches the jugal. Therefore, an extremely small portion of the maxilla is involved in the anteroventral rim of the orbit (see Fig. 7A). Both sides of the skull of *C. mineirus* are remarkably diferrent to the condition of *C. stenognathus*, in which a considerable portion of the maxilla forms part of the orbital rim (Pol et al., 2014).

*Caipirasuchus mineirus* has well-developed maxillo-palatine fenestrae, suggesting that this features should be removed from the diagnosis of *C. stenognathus* (Pol et al., 2014). In addition, Pol et al. (2014) proposed as autopomorphic for *C. stenognathus* a pointed posterodorsal process of the lacrimal at the anterodorsal corner of orbit that overhangs the posterior opening of lacrimal duct. This feature is also seen in *C. mineirus*, thus this character is not exclusive of *C. stenognathus*. Furthermore, in *C. stenognathus* the pointed posterodorsal process of the lacrimal is evident on the left side; however, this condition is not seen on the right side, where the lacrimal has a likely equilateral triangle-shape in dorsal view, without a conspicuous posterodorsal process (see Pol et al., 2014: fig. 9).

Other cited autopomorphies of *C. stenognathus* include: (1) the anterolateral corner of the pterygoid flange forms an elevated platform that projects medially and is overlapped by posterior end of palatine bar; (2) a smooth and recessed face for posterior palpebral bone in the postorbital that extends posteriorly underneath ear-flap groove; (3) anteroposterior constriction at the medial origin of pterygoid flanges (lateral to choanal groove); and (4) the choanal septum of pterygoid tapering posteriorly and bearing marked groove on its ventral surface (Pol et al., 2014). Features 1, 3, and 4 are seen in *C. mineirus*, thus they should no longer be autapomorphies of *C. stenognathus*. However, the choanal septum is transversely narrower and anteroposteriorly elongated in *C. mineirus*. Feature 2 is still autapomorphic for *C. stenognathus*, whereas *C. mineirus* has a reduced facet, fairly similar to that present in *C. paulistanus*.

We also observed that in *C. stenognathus* the transversal suture between the postorbital and the squamosal, in dorsal view, is positioned far posteriorly than in the remaining species of *Caipirasuchus*, being located at the level of the posterior rim of the supratemporal fenestra. In *C. paulistanus*, *C. montealtensis*, and *C. mineirus* the dorsal postorbital-squamosal suture is at the midpoint of this fenestra. Accordingly, this condition is here considered as a new diagnostic feature for *C. stenognathus*.

Another important difference between the *C. mineirus* and the other *Caipirasuchus* species is that they come from distantly related outcrops of the Adamantina Formation. *C. mineirus* was found in an outcrop (in the state of Minas Gerais) that is 150 km north than the northernmost outcrop with the other three species (in the state of São Paulo), and we have no definitive evidence to unambiguously consider the outcrops from these different states are coeval. Thus, the differences among close relative species, as in the case of *Caipirasuchus*, would be the result of slightly different ages for each deposit (see discussions on taxonomic aproches versus stratigraphic control in Kellner, 2010) (see below) rather than geographic variations. Other approach to be noted is that *Caipirasuchus mineirus* occurs in a stratigraphic layer below the level of *Campinasuchus dinizi*. This latter species is considered a pissarrachampsine (Montefeltro, Larsson & Langer, 2011), a clade positioned as the sister taxon of baurusuchines (*Baurusuchus* spp. and *Stratiotosuchus maxhechti*). Particularly, *Baurusuchus* occurs in the state of São Paulo with *Caipirasuchus stenognathus* and other sphagesaurid material (e.g., *Armadillosuchus arrudai*), contrary to what is seen in Triângulo Mineiro. Recently, Pinheiro et al. (2018) identified four Crocodyliformes Assemblages Zones (AZ) within levels of the Adamantina and correlated or partially correlated formations in Minas Gerais and São Paulo states within a sequence stratigraphic framework. The authors also mentioned the lack of appropriated biostratigraphic correlations and the difficult to perform it, in part due to taxonomic problem with some taxa and also biases in the fossil record. In this scheme, the “FTA” site and other outcrops (e.g., General Salgado) are not included at the moment but the presence of more than one AZ with sphagesaurids and baurusuchids of different ages is highlighted. This preliminary result can add support to the idea that the fossiliferous outcrop of “FTA” (Campina Verde, Triângulo Mineiro) has a different age with regard to the outcrops from western São Paulo (i.e., General Salgado), but further combined studies are still needed.

Finally, as for other notosuchians from the Bauru Group (e.g., species of *Baurusuchus* and *Mariliasuchus*), the morphological disparity seen among the different species of *Caipirasuchus* could be the result of different ontogenetic stages of the specimens. However, as multiples individuals from a same species (ideally from the same outcrop) are necessary for studying the morphological differences associated to ontogenetic changes, this subject is still poorly explored for notosuchians in general (apart from a few exceptions, such as *Mariliasuchus amarali*; Vasconcellos & Carvalho, 2005, 2006). In the case of *Caipirasuchus*, more than one specimen is known only for *C. montealtensis* and *C. paulistanus*. Thus, at the moment, we regard the exclusive features of *C. mineirus* as diagnostic for a new species, even though we acknowledge that more specimens could change this scenario. The same scenario could be valid for other notosuchians. For example, the two known species of *Mariliasuchus* come from the same outcrop and are mainly differentiated by cranial robustness (Carvalho & Bertini, 1999; Nobre et al., 2007). Consequently, they were considered synonyms (Geroto, 2015). *Baurusuchus* includes three species from closely related outcrops, in western São Paulo state (Price, 1945; Carvalho, Campos & Nobre, 2005; Nascimento & Zaher, 2010) and intraspecific variations of these species are poorly known. For peirosaurids, for example, *Peirosaurus torminni* and *Uberabasuchus terrificus* come from the same outcrop in Minas Gerais state and were considered as possibly synonyms (Martinelli et al., 2012b).

Based on the combination of features seen in *Caipirasuchus mineirus*, its stratigraphical relationship with *Campinasuchus dinizi* (and the relationship of it with baurusuchines), the diferent faunal associatios, its geographical locations and the the poor knowledge of ontogenetic changes in notosuchians, we justify at the moment the taxonomic proposal here presented.

### The first sphagesaurid in minas gerais state?

Sphagesaurid remains from the Triângulo Mineiro region were reported at least two times previously. The first is a brief mention of “*Sphagesaurus* material” in an conference abstract (Kellner, Campos & Price, 1995), without further specification of the available remains. The material, under collection number MN 3840-V, is from Iturama (Minas Gerais), next to Campina Verde municipality, where extensive outcrops of the Adamantina Formation are found. At the moment of that abstract, the available data on *Sphagesaurus* (and sphagesaurids in general) was extremely limited when compared with the current knowledge on the group, and only the formal description of specimen MN 3840-V can clarify its taxonomy.

The second possible sphagesaurid is *Labidiosuchus amicum*, from the Serra da Galga Member of the Marília Formation, in Peirópolis, Uberaba municipality (Kellner et al., 2011b). The holotype includes an incomplete lower jaw with partial dentition. Due to its incompleteness, *L. amicum* was originally included within Notosuchia, as closely related to *Adamantinasuchus*, *Mariliasuchus*, and *Notosuchus*. However, the phylogenetic studies of Pol et al. (2014) placed *L. amicum* in a polytomy with *Mariliasuchus* and Sphagesauridae (also obtained in our current analysis; see below). Accordingly, due to its geological provenance it would be the youngest record of an “advanced notosuchian.” Only further specimens will elucidate its taxonomy and, consequently, *C. mineirus* is the best unequivocal occurrence of sphagesaurids in Minas Gerais.

### Phylogenetic placement of *C. mineirus*

**Figure 35:**
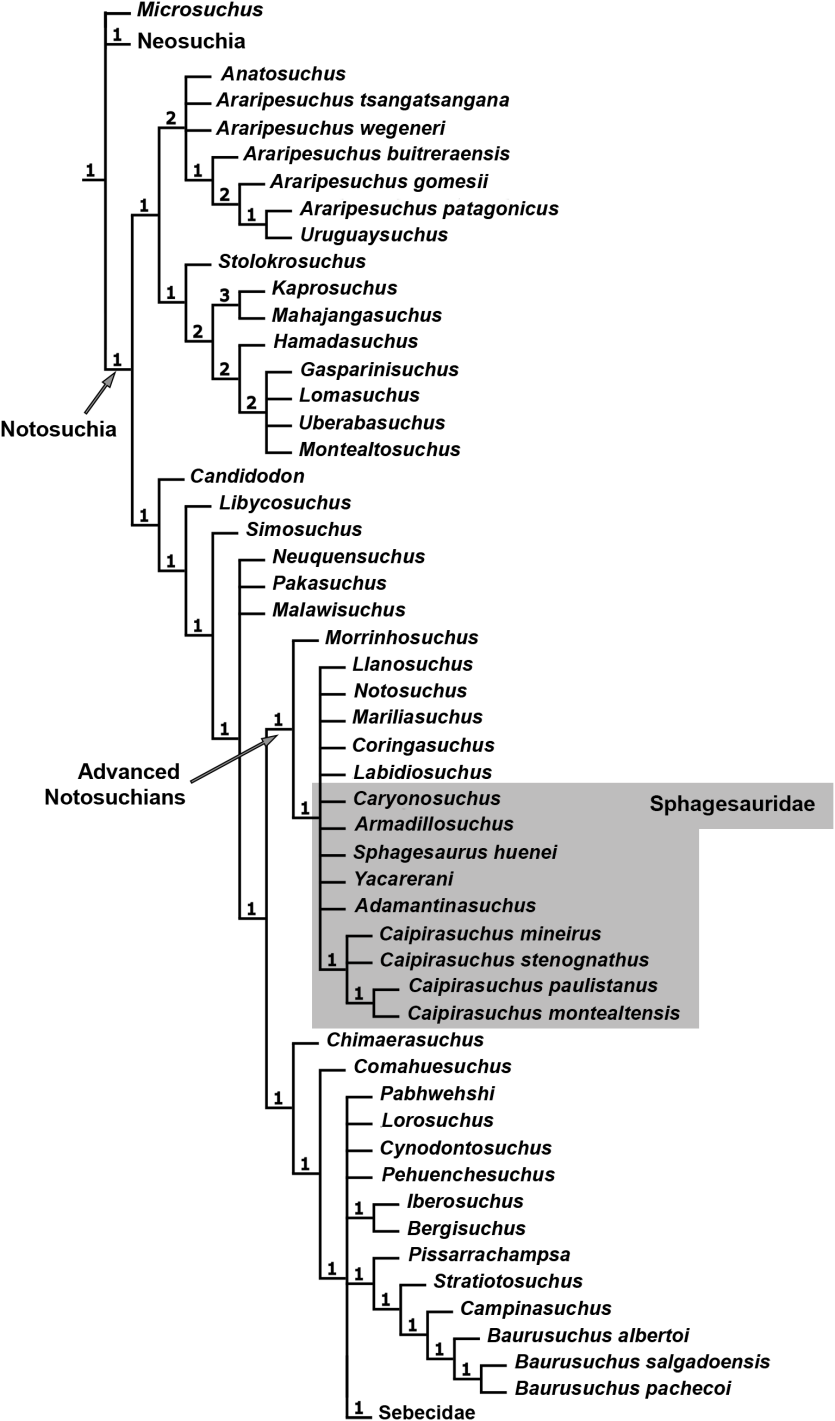
Phylogenetic position of *C. mineirus*. Reduced strict consensus tree depicting the phylogenetic relationships of notosuchians.

The phylogenetic analysis, with the inclusion of the holotype of *C. mineirus* in the dataset of Fiorelli et al. (2016), resulted in 133,920 MPCs. The MPCs have 1,702 steps, with an ensemble consistency index (CI) of 0.31, an ensemble retention index (RI) of 0.74, and a rescaled consistency index (RC) of 0.23. The phylogenetic relationships of notosuchians are shown in Fig. 35, including Bremer values (the complete strict consensus cladogram is available in File S2).

As in Fiorelli et al. (2016), Sphagesauridae and most members of the “advanced notosuchians” are collapsed (Fig. 35). *Morrinhosuchus* is placed as the sister taxon to all other “advanced notosuchians,” represented in our results by a massive politomy including *Notosuchus*, *Mariliasuchus*, *Labidiosuchus*, *Yacarerani*, *Adamantinasuchus*, *Sphagesaurus*, *Caryonosuchus*, *Armadillosuchus* and a clade with the *Caipirasuchus* species. The *Caipirasuchus* clade shows unresolved the relationships between *C. mineirus*, *C. stenognathus* and the clade formed by *C. montealtensis* and *C. paulistanus*. The placement of the holotype (CPPLIP 1463) of *C. mineirus* within the *Caipirasuchus* genus is supported by the present analysis (see list of synapomorphies in File S2). Even though the relationships of their members are not totally understood, *C. mineirus* can be diagnosed by a mosaic of features (as demonstrated above) that support a new species.

With the exception of uruguaysuchids and some peirosaurid subclades, all other clades have low Bremer values (i.e., 1) (Fig. 35). Sphagesauridae is recovered as monophyletic in most of the recent phylogenetic analyses (Pol et al., 2014; Leardi, Fiorelli & Gasparini, 2015a; Leardi et al., 2015b; Fiorelli et al., 2016), especially when some extremely incomplete based taxa are removed (e.g., *Microsuchus*, *Neuquensuchus*, *Coringasuchus*). Some other contributions have showed this clade as paraphyletic or with a distinctive composition (O’Connor et al., 2010; Turner & Sertich, 2010; Iori et al., 2013). The inclusion of *C. mineirus* mostly added scores on postcranial data for sphagesaurids, which is well known for *Yacarerani* (Leardi et al., 2015b) and unknown or extremely limited in other members of the clade (e.g., *Sphagesaurus*, *Caryonosuchus*, *Armadillosuchus*, *Caipirasuchus* species).

**Figure 36:**
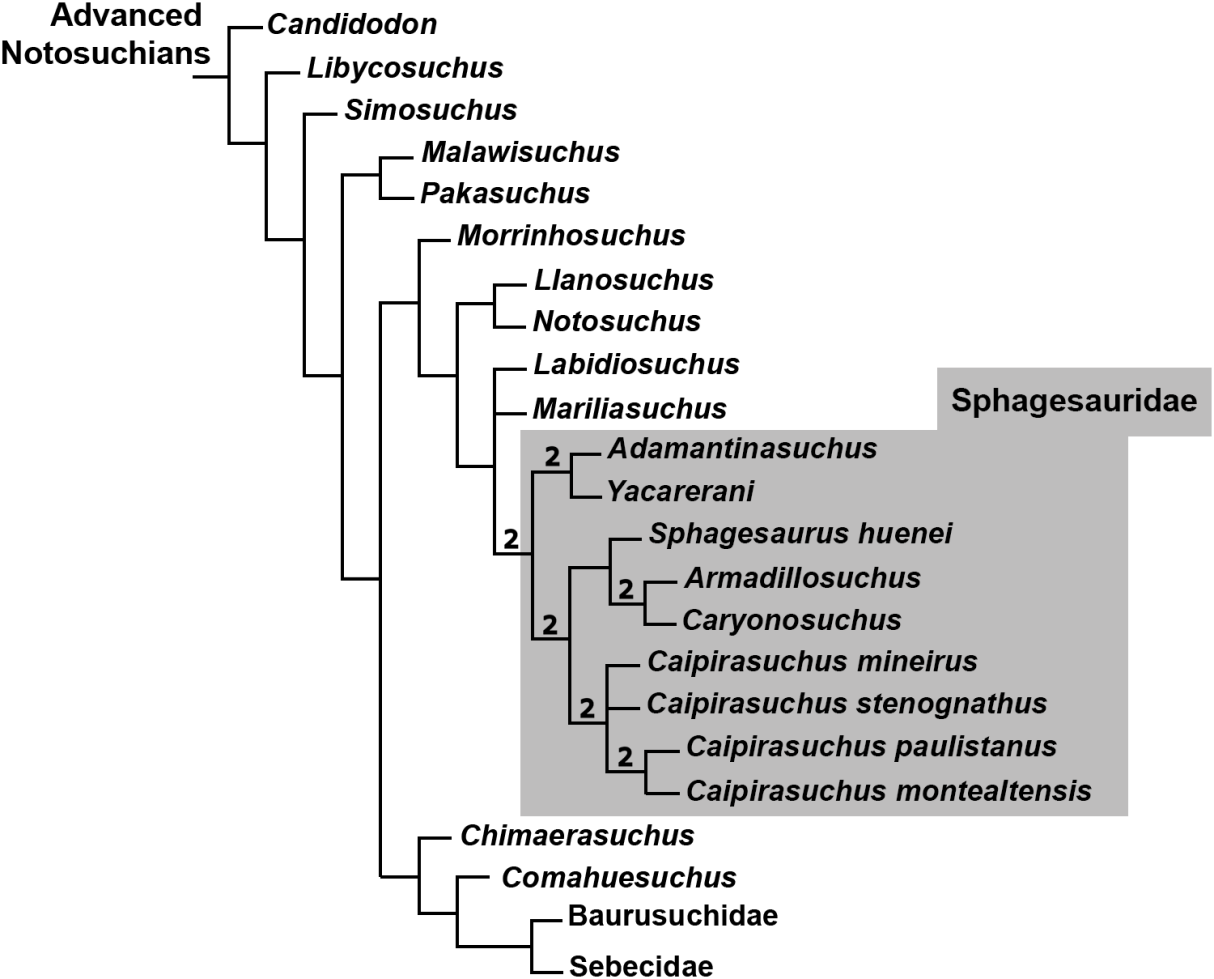
Phylogenetic position of *C. mineirus*. Reduced strict consensus after pruning *Pehuenchesuchus*, *Microsuchus*, *Neuquensuchus*, *Coringasuchus*, and *Pabwehshi*.

After pruning incomplete taxa (i.e., *Pehuenchesuchus*, *Microsuchus*, *Neuquensuchus*, *Coringasuchus*, and *Pabwehshi*), following the protocol used by Fiorelli et al. (2016), a new analysis resulted in 360 MPCs, with 1,687 steps, a ensemble CI of 0.31, a ensemble RI of 0.75, and a RC index of 0.23. In the strict consensus (Fig. 36), as in the previous analysis, *Caipirasuchus mineirus* and *C. stenognathus* form a polytomy with *C. paulistanus* and *C. montealtensis*. Apart from this polytomy, however, the relationships within “advanced notosuchians” are fully resolved, as in previous analyses (Pol et al., 2014; Leardi, Fiorelli & Gasparini, 2015a; Leardi et al., 2015b; Fiorelli et al., 2016), with higher Bremer values (Fig. 36) than in the complete analysis (Fig. 35). The postcranial data added for *C. mineirus* in the matrix does not influence in the resolution of “advanced notosuchians,” suggesting that within this group the postcranium seems to be more conservative than craniodental features. Although, missing data on postcranial characters is still large for several notosuchians, and the great cranial and dental disparity observed among this clade clearly determinate their phylogenetic topologies. However, for the case of peirosaurids, sebecids and uruguaysuchids the postcranium has a significant contribution to determinate their notosuchian affinities (Godoy et al., 2016).

### Postcranial anatomy of *C. mineirus*

The postcranial anatomy of sphagesaurids is still poorly known. A few appendicular bones were published for *Adamantinasuchus navae* (Nobre & Carvalho, 2006), but more complete material awaits its formal publication. *Armadillosuchus arrudai* includes the anterior portion of the dorsal armor (Marinho & Carvalho, 2009), in addition to a few postcranial bones not described yet. *Y. boliviensis* includes several elements of the axial and appendicular skeleton (Leardi et al., 2015b), being one of the best described sphagesaurid skeletons. *Caipirasuchus* postcrania include a partial skeleton assigned to *C. montealtensis*, another referred as *C. paulistanus*, and a third as *Caipirasuchus* sp., that were used to estimate corporal proportions for the genus (Iori, Marinho & Carvalho, 2016). Finally, *C. mineirus* is represented by the partial skeleton above described. Postcranial remains of closely related notosuchians are available for *Mariliasuchus amarali* (Nobre & Carvalho, 2013) and *N. terrestris* (Pol, 2005; Fiorelli & Calvo, 2008).

The postcranial skeleton of *C. mineirus* does not differs significantly from that of other “advanced notosuchians.” The limbs are slightly slender than in *Mariliasuchus* and *Notosuchus*, but not to the degree seen in uruguaysuchids (e.g., *Araripesuchus*; Turner, 2006) or *Neuquensuchus* (Fiorelli & Calvo, 2007; Lio et al., 2018). The postcranial skeleton also has some typically notosuchian features, such as, among others, a tall and laminar neural spine, with anterior and posterior medial laminae in anterior dorsal vertebrae; postzygapophysis and diapophysis forming a wide plataform, bearing a conspicuous fossa at the base of the neural spine in dorsal and caudal vertebrae; and three sacral vertebrae (possibly 2° and 3° caudals fused); femur almost straight in anterior view; and a semicircular calcaneal anterior roller in lateral view, with an anterodorsally rounded edge. The iliac morphology is partially accessed, but it is similar to other “advanced notosuchians” (e.g., *Notosuchus*, *Mariliasuchus*), with a lateral expansion of its dorsal surface, forming a more developed acetabular roof, indicative of a parasagittal (erect to semierect) posture in these forms (e.g., Pol, 2005; Riff & Kellner, 2011; *contra* Nobre & Carvalho, 2013).

According to Iori, Marinho & Carvalho (2016), the tail of *Caipirasuchus* (i.e., *C. montealtensis* and *C. paulistanus*) is long, representing about half of total body length. However, in the holotype of *C. mineirus* the tail represents less than the total body length (including the head), resulting in a relatively short tail, although not as short as seen in *Simosuchus* (Georgi & Krause, 2010; Hill, 2010).

Another conspicuous difference is in the presence of two parallel rows of parasagittal osteoderms, which in *C. mineirus* seems to be present from about the ninth dorsal vertebra to the end of the tail. As aforementioned, it could be due to taphonomic processes but the size reduction of dorsal osteoderms in comparison to sacral and anterior caudal ones would support the idea that osteoderms are absent in cervical and anterior dorsal regions. Consequently, this taxon exhibits an incomplete dorsal armor, different to what is seen in *C. montealtensis* and *C. paulistanus* (Iori, Marinho & Carvalho, 2016). Additionally, we interpreted that there is no antero-posterior interlocking among osteoderms of the same row, a condition also seen in *Mariliasuchus* (Nobre & Carvalho, 2013) and *Adamantinasuchus* (AGM, 2017, personal observation). On the other hand, *C. montealtensis*, *C. paulistanus* (Iori, Marinho & Carvalho, 2016) and *Notosuchus* (Pol, 2005) have osteoderms that overlap one another along the row. The dorsal armor in notosuchians seems to have a complex evolutionary history, from heavily armored forms (e.g., peirosaurids, some sebecosuchians, *Simosuchus*; Marinho & Carvalho, 2009; Hill, 2010; Nascimento & Zaher, 2010; Tavares, Ricardi-Branco & Carvalho, 2015) to animals which tend to reduce its dorsal armor (e.g., *Araripesuchus*, *Adamantinasuchus, Caipirasuchus mineirus*; *Campinasuchus*; Turner, 2006; Cotts et al., 2017; AGM, 2017, personal observation) or it is apparently lost (e.g., *Pissarrachampsa*; Godoy et al., 2016). Among notosuchians, Godoy et al. (2016) discussed the lack of osteoderms in the baurusuchid *Pissarrachampsa*, but they did not find positive evidence to justify its loss as related to body size reduction or related to bracing system for terrestrial locomotion. Among sphagesaurids, the osteoderm yield is remarkably diverse: *Armadillosuchus* has a massive yield bearing several rows of osteoderms (Marinho & Carvalho, 2009), *C. montealtensis* and *C. paulistanus* have two parallel rows of imbricated parasagittal osteoderms (Iori, Marinho & Carvalho, 2016), and *Adamantinasuchus* has few disminute oval-shaped osteoderms, that are not in contact with each other (AGM, 2017, personal observation). Osteoderms are not known for *Sphagesaurus*, *Yacarerani*, *Caryonosuchus*, and *Caipirasuchus stenognathus*. We have no unambiguous data to explain the reduction of the osteoderm yield particularly in *C. mineirus*, and can not relate it to corporal size reduction (based on baurusuchids it seems not related to size; Godoy et al., 2016; Cotts et al., 2017), postural anatomy or regional environment conditions (most of these diverse animals were found in the same geological formation, under similar climate conditions; see Carvalho et al., 2010).

With regard to the limbs proportions, *C. mineirus* is similar to *C. montealtensis* and *C. paulistanus*, with the hindlimbs twice as long as the forelimbs (Iori, Marinho & Carvalho, 2016). Nonetheless, both the shorter tail and the reduction of the parasagittal osteoderms, in comparison to *C. montealtensis* and *C. paulistanus* (Iori, Marinho & Carvalho, 2016), are of taxonomic value and also suggests that *C. mineirus* was more gracile than these two *Caipirasuchus* species (in *C. stenognathus*, the postcranium is unknown). However, as aforementioned, the holotype of *C. mineirus* is represented by a juvenile to subadult individual and further specimens are needed to corroborate these differences. Gracile forms seem to be a common feature among notosuchians, which was adquired convergently in diferent lineages, such as in uruguaysuchids (Turner, 2006), *Pakasuchus* (O’Connor et al., 2010), *Caipirasuchus* species (Iori, Marinho & Carvalho, 2016), and *Adamantinasuchus* (Nobre & Carvalho, 2006).

## Conclusions

We fully describe the first *Caipirasuchus* skeleton from the state of Minas Gerais, southeastern Brazil. The new specimen constitutes the holotype of a new species, which can be differentiated from the three previously known species of the genus. The new species, *C. mineirus*, provides features in the skull, lower jaws and postcranium that are poorly known in the other *Caipirasuchus* species due to bad preservation. Moreover, some features considered diagnostic for the other three species are now demostrated to have a broader distribution among the genus, reducing the number of autapomorphies for each species, especially for *C. paulistanus* and *C. montealtensis*, which are only differentiated by the presence of a fossa on the pterygoid in the latter. Further specimens and more studies will permit to evaluate the taxonomy of the four species of *Caipirasuchus*. This number is likely an overestimation for a medium-sized terrestrial taxon in a relatively restricted area, but the chronostratigraphic relationship of each outcrop yielding *Caipirasuchus* material is also unknown. As such, we cannot unambiguously assert that all species are coeval and that the observed differences are result of intraspecific variations. The phylogenetic analysis supports the placement of CPPLIP 1463 as part of the genus *Caipirasuchus* and its postcranial anatomy is similar to that of other “advanced notosuchians,” such as *Mariliasuchus*, *Notosuchus*, and *Yacarerani*.

## Supplemental Information

10.7717/peerj.5594/supp-1 Supplemental Information 1 S1. Data matrix including *Caipirasuchus mineirus*.

10.7717/peerj.5594/supp-2 Supplemental Information 2 S2. Data score for *Caipirasuchus mineirus*, full strict consensus cladogram including all taxa, and list of synapomorphies.

## Institutional abbreviations

CPPLIP: Centro de Pesquisas Paleontológicas “Llewellyn Ivor Price,” Universidade Federal do Triângulo Mineiro (UFTM), Peirópolis, Uberaba County, Minas Gerais, Brazil
MACN: Museo Argentino de Ciencias Naturales “Bernardino Rivadavia” (PV, Paleovertebrate Collection; N, Neuquén Province; RN, Río Negro Province), Buenos Aires, Argentina
MN: Museu Nacional, Rio de Janeiro, Brazil
MPMA: Museu de Paleontologia “Prof. Antonio Celso de Arruda Campos”, Monte Alto, São Paulo, Brazil
MZSP-PV: Museu de Zoologia, Universidade de São Paulo, São Paulo, Brazil
RCL: Museu de Ciências Naturais, Pontifícia Universidade Católica de Minas Gerais, Belo Horizonte, Minas Gerais, Brazil
UFRJ: Museu de Paleontologia e Estratigrafia, Universidade Federal de Rio de Janeiro, Rio de Janeiro, Brazil.

## Additional Information and Declarations

### Data Availability

The following information was supplied regarding data availability: The Supplementary Files include a complete cladogram and the data matrix. Additionally, Specimen CPPLIP 1463 is housed in the Centro de Pesquisas Paleontológicas L. I. Price (CPPLIP) of the Universidade Federal do Triângulo Mineiro (UFTM), Uberaba, Minas Gerais, Brazil.

### New Species Registration

The following information was supplied regarding the registration of a newly described species: **Publication LSID:** urn:lsid:zoobank.org:pub:EF45EECD-02FD-433C-B747-21341A7CF7C2. **Caipirasuchus mineirus sp. nov. LSID:** urn:lsid:zoobank.org:act:8A54B326-3323-4EAC-9811-A8146AE110B5
