# Supplementary material for: The first Caipirasuchus (Mesoeucrocodylia, Notosuchia) from the Late Cretaceous of Minas Gerais, Brazil: new insights on sphagesaurid anatomy and taxonomy
Source: PeerJ. 2018 Sep 5;6:e5594. doi: 10.7717/peerj.5594 (PMC6129144; doi:10.7717/peerj.5594)
Supplement: Supplemental Information 2 [file peerj-06-5594-s002.docx]

**S2 File.** Supporting Information for Martinelli et al. “The first *Caipirasuchus* (Mesoeucrocodylia, Notosuchia) from the Late Cretaceous of Minas Gerais, Brazil: new insights on sphagesaurid anatomy and taxonomy”

**The data score for *Caipirasuchus mineirus* is as follow:**

101?00?1020000?11000111111001110110221101100?1?12011?1100??0?0?0311111110100110001?1??1????00?000[12]0001112112??110012?01201101?110100001011[01]11011010111???01000000101000?001100???000010?11100011211100100011?101100000000?000?00?0?11101011000000?00000?000100000???10?000?01?10?1000010??0??00?0001000???0?011?1????0100???0???????01111?00?1000??????00??00?11111?10?0[01]012101211101011011110[01]1111??00111101010000?000000111??????????00?????????1??100

**Bremer values.** Strict consensus cladogram of 133,920 MPTs, with Bremer support values, depicting the phylogenetic relationships of *Caipirasuchus mineirus*, based on the data matrix of Fiorelli et al., (2016).


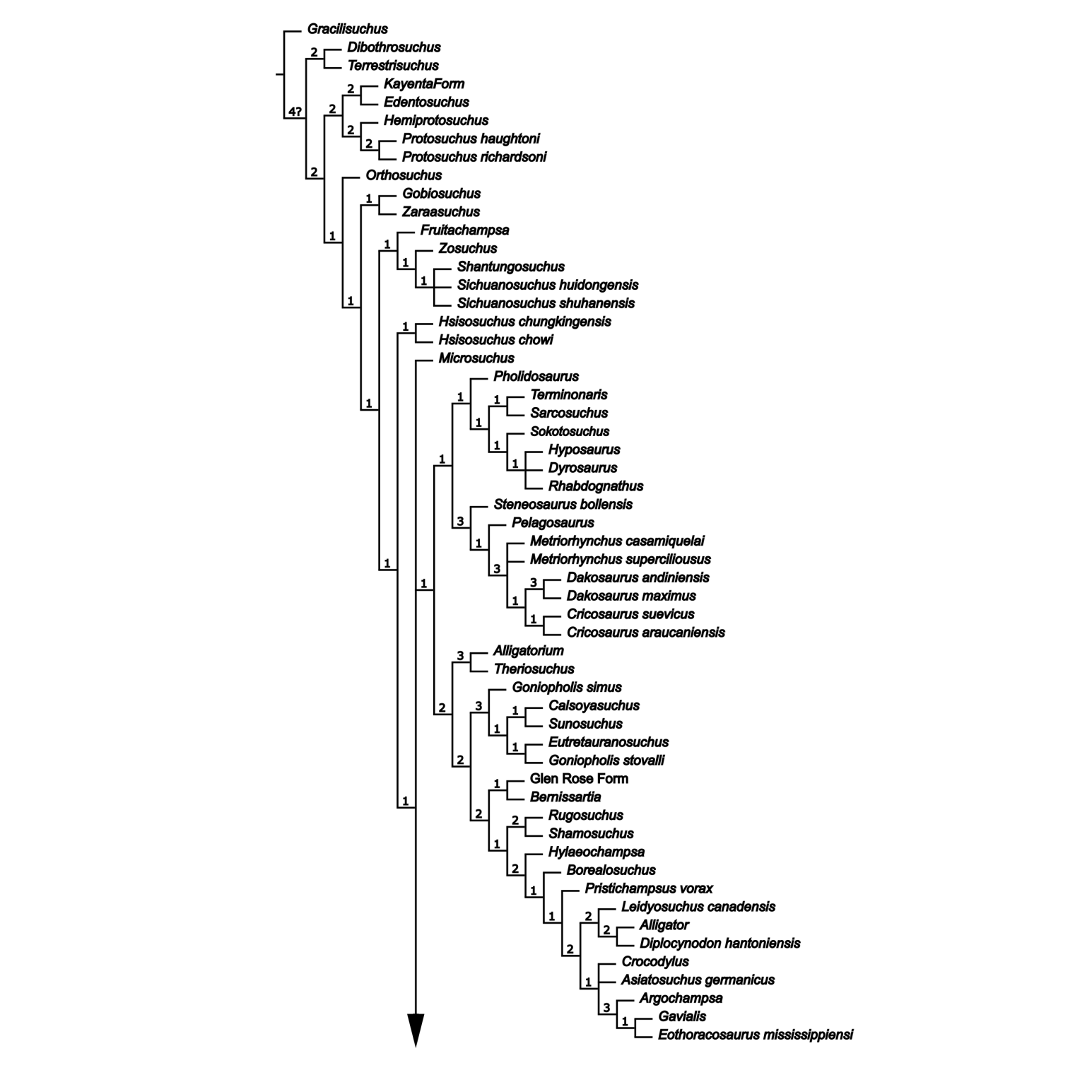


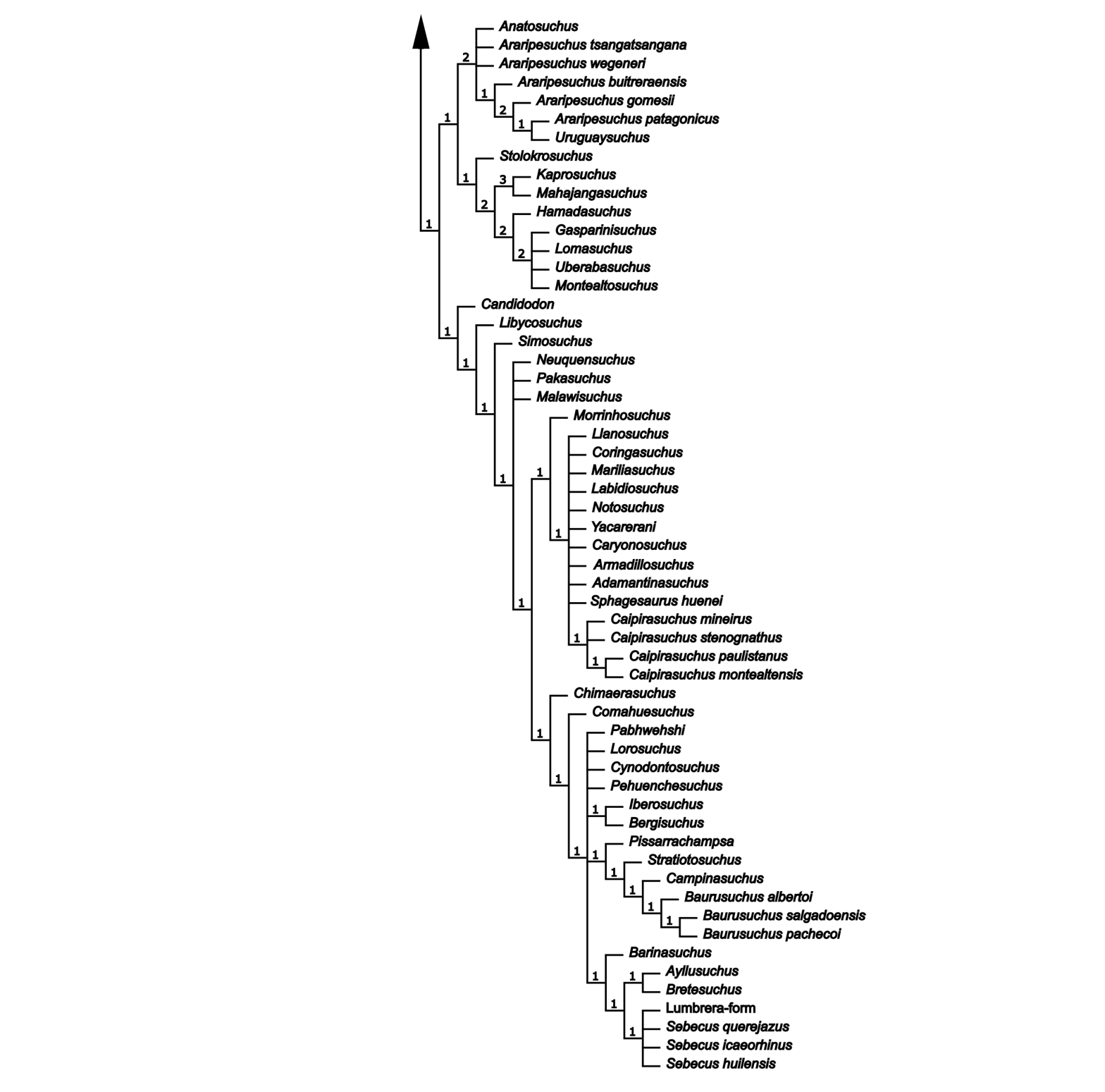


**Synapomorphies.** Strict consensus cladogram with numbers of the nodes for the list of synapomorphies.

ÚÄÄ0 Gracilisuchus

³ ÚÄÄ2 Dibothrosuchus

³ ÚÄÄ114ÁÄÄ1 Terrestrisuchus

ÃÄÄ113´ ³ ÚÄÄ8 KayentaForm

³ ³ ÚÄÄ121ÁÄÄ7 Edentosuchus

³ ³ ÚÄÄ118´ ÚÄÄ5 Hemiprotosuchus

ÀÄÄ115´ ³ ÀÄÄ117´ ÚÄÄ4 Protosuchus_haughtoni

³ ³ ÀÄÄ116ÁÄÄ3 Protosuchus_richardsoni

³ ³ ÚÄÄ6 Orthosuchus

ÀÄÄ119´ ³ ÚÄÄ10 Gobiosuchus

³ ³ ÚÄÄ122ÁÄÄ9 Zaraasuchus

³ ³ ³ ÚÄÄ17 Fruitachampsa

ÀÄÄ120´ ³ ÚÄÄ126´ ÚÄÄ14 Zosuchus

³ ³ ³ ÀÄÄ125´ ÚÄÄ13 Shantungosuchus

³ ³ ³ ÀÄÄ124ÅÄÄ12 Sichuanosuchus_huidongensis

³ ³ ³ ÀÄÄ11 Sichuanosuchus_shuhanensis

ÀÄÄ123´ ³ ÚÄÄ16 Hsisosuchus_chungkingensis

³ ³ ÚÄÄ128ÁÄÄ15 Hsisosuchus_chowi

³ ³ ³ ÚÄÄ110 Microsuchus

³ ³ ³ ³ ÚÄÄ105 Pholidosaurus

³ ³ ³ ³ ³ ÚÄÄ107 Terminonaris

ÀÄÄ127´ ³ ³ ÚÄÄ195´ ÚÄÄ196ÁÄÄ106 Sarcosuchus

³ ³ ³ ³ ÀÄÄ194´ ÚÄÄ102 Sokotosuchus

³ ³ ³ ³ ÀÄÄ193´ ÚÄÄ104 Hyposaurus

³ ³ ³ ³ ÀÄÄ192ÅÄÄ103 Dyrosaurus

³ ³ ³ ÚÄÄ187´ ÀÄÄ101 Rhabdognathus

³ ³ ³ ³ ³ ÚÄÄ94 Steneosaurus_bollensis

³ ³ ³ ³ ³ ³ ÚÄÄ93 Pelagosaurus

³ ³ ³ ³ ÀÄÄ186´ ³ ÚÄÄ96 M._casamequelai

³ ³ ³ ³ ÀÄÄ185´ ÃÄÄ95 M._superciliousus

ÀÄÄ129´ ³ ³ ÀÄÄ188´ ÚÄÄ100 D._andiniensis

³ ³ ³ ³ ÚÄÄ191ÁÄÄ99 D._maximus

³ ÃÄÄ166´ ÀÄÄ190´ ÚÄÄ98 C._suevicus

³ ³ ³ ÀÄÄ189ÁÄÄ97 C._araucaniensis

³ ³ ³ ÚÄÄ74 Alligatorium

³ ³ ³ ÚÄÄ164ÁÄÄ73 Theriosuchus

³ ³ ³ ³ ÚÄÄ75 Goniopholis_simus

³ ³ ³ ³ ³ ÚÄÄ83 Calsoyasuchus

³ ³ ÀÄÄ165´ ÚÄÄ167´ ÚÄÄ171ÁÄÄ78 Sunosuchus

³ ³ ³ ³ ÀÄÄ170´ ÚÄÄ77 Eutretauranosuchus

³ ³ ³ ³ ÀÄÄ169ÁÄÄ76 Goniopholis_stovalli

³ ³ ÀÄÄ168´ ÚÄÄ82 Glen_Rose_Form

³ ³ ³ ÚÄÄ175ÁÄÄ80 Bernissartia

ÀÄÄ136´ ³ ³ ÚÄÄ108 Rugosuchus

³ ÀÄÄ174´ ÚÄÄ172ÁÄÄ79 Shamosuchus

³ ³ ³ ÚÄÄ81 Hylaeochampsa

³ ÀÄÄ173´ ³ ÚÄÄ84 Borealosuchus

³ ÀÄÄ176´ ³ ÚÄÄ85 Pristichampsus_vorax

³ ÀÄÄ177´ ³ ÚÄÄ88 Leidyosuchus_canadensis

³ ÀÄÄ178´ ÚÄÄ183´ ÚÄÄ92 Alligator

³ ³ ³ ÀÄÄ184ÁÄÄ91 Diplocynodon_hantoniensis

³ ÀÄÄ182´ ÚÄÄ90 Crocodylus

³ ³ ÃÄÄ89 Asiatosuchus_germanicus

³ ÀÄÄ181´ ÚÄÄ109 Argochampsa

³ ÀÄÄ180´ ÚÄÄ87 Gavialis

³ ÀÄÄ179ÁÄÄ86 Eothoracosaurus

³ ÚÄÄ64 Anatosuchus

³ ÃÄÄ63 A._tsangatsangana

³ ÚÄÄ133ÅÄÄ62 A._wegeneri

³ ³ ³ ÚÄÄ61 A._buitreraensis

³ ³ ÀÄÄ132´ ÚÄÄ59 A._gomesii

³ ³ ÀÄÄ131´ ÚÄÄ60 A._patagonicus

³ ÚÄÄ134´ ÀÄÄ130ÁÄÄ18 Uruguaysuchus

³ ³ ³ ÚÄÄ72 Stolokrosuchus

³ ³ ³ ³ ÚÄÄ71 Kaprosuchus

³ ³ ÀÄÄ162´ ÚÄÄ163ÁÄÄ70 Mahajangasuchus

³ ³ ³ ³ ÚÄÄ69 Hamadasuchus

³ ³ ÀÄÄ161´ ³ ÚÄÄ68 Gasparinisuchus

ÀÄÄ135´ ÀÄÄ160´ ÃÄÄ67 Lomasuchus

³ ÀÄÄ159ÅÄÄ66 Uberabasuchus

³ ÀÄÄ65 Montealtosuchus

³ ÚÄÄ19 Candidodon

³ ³ ÚÄÄ20 Libycosuchus

ÀÄÄ137´ ³ ÚÄÄ21 Simosuchus

³ ³ ³ ÚÄÄ111 Neuquensuchus

ÀÄÄ138´ ³ ÃÄÄ23 Pakasuchus

³ ³ ÃÄÄ22 Malawisuchus

ÀÄÄ139´ ³ ÚÄÄ39 Morrinhosuchus

³ ³ ³ ÚÄÄ112 Llanosuchus

³ ³ ³ ÃÄÄ38 Coringasuchus

³ ³ ³ ÃÄÄ37 Adamantinasuchus

ÀÄÄ140´ ÚÄÄ144´ ÃÄÄ36 Yacarerani

³ ³ ³ ÃÄÄ31 Armadillosuchus

³ ³ ³ ÃÄÄ30 Sphagesaurus

³ ³ ÀÄÄ143ÅÄÄ29 Caryonosuchus

³ ³ ÃÄÄ28 Labidiosuchus

³ ³ ÃÄÄ27 Mariliasuchus

³ ³ ÃÄÄ25 Notosuchus

ÀÄÄ142´ ³ ÚÄÄ35 Caipirasuchus_mineirus

³ ³ ÃÄÄ32 Caipirasuchus_stenognathus

³ ÀÄÄ146´ ÚÄÄ34 Caipirasuchus_paulistanus

³ ÀÄÄ147ÁÄÄ33 Caipirasuchus_montealtensis

³ ÚÄÄ24 Chimaerasuchus

³ ³ ÚÄÄ26 Comahuesuchus

³ ³ ³ ÚÄÄ58 Pabhwehshi

ÀÄÄ141´ ³ ÃÄÄ56 Lorosuchus

³ ³ ÃÄÄ47 Cynodontosuchus

ÀÄÄ145´ ÃÄÄ46 Pehuenchesuchus

³ ³ ÚÄÄ49 Iberosuchus

³ ÃÄÄ154ÁÄÄ48 Bergisuchus

³ ³ ÚÄÄ41 Pissarrachampsa

ÀÄÄ151ÅÄÄ150´ ÚÄÄ45 Stratiotosuchus

³ ÀÄÄ149´ ÚÄÄ40 Campinasuchus

³ ÀÄÄ148´ ÚÄÄ42 B._albertoi

³ ÀÄÄ152´ ÚÄÄ44 B._salgadoensis

³ ÀÄÄ153ÁÄÄ43 B._pachecoi

³ ÚÄÄ51 Barinasuchus

³ ³ ÚÄÄ55 Ayllusuchus

ÀÄÄ157´ ÚÄÄ155ÁÄÄ50 Bretesuchus

³ ³ ÚÄÄ57 Lumbrera_form

ÀÄÄ156´ ÃÄÄ54 S._querejazus

ÀÄÄ158ÅÄÄ53 S._icaeorhinus

ÀÄÄ52 S._huilensis

Synapomorphies (Node numbers refer to nodes in consensus)

Gracilisuchus :

All trees:

No autapomorphies:

Terrestrisuchus :

All trees:

Char. 432: 0 --> 1

Dibothrosuchus :

All trees:

Char. 14: 0 --> 1

Char. 78: 0 --> 1

Char. 89: 0 --> 2

Protosuchus_richardsoni :

All trees:

Char. 164: 1 --> 0

Protosuchus_haughtoni :

All trees:

Char. 205: 1 --> 0

Char. 279: 0 --> 1

Hemiprotosuchus :

All trees:

Char. 32: 0 --> 1

Orthosuchus :

All trees:

Char. 1: 0 --> 1

Char. 10: 0 --> 1

Char. 104: 1 --> 2

Char. 107: 0 --> 4

Char. 130: 0 --> 1

Char. 141: 1 --> 0

Char. 152: 0 --> 1

Char. 162: 0 --> 1

Char. 211: 0 --> 1

Char. 284: 1 --> 0

Char. 436: 0 --> 1

Edentosuchus :

All trees:

Char. 66: 1 --> 2

Char. 120: 0 --> 1

Char. 124: 0 --> 1

Char. 129: 0 --> 1

Char. 138: 0 --> 1

Kayenta_Form :

All trees:

Char. 118: 0 --> 1

Zaraasuchus :

All trees:

Char. 198: 0 --> 1

Gobiosuchus :

All trees:

No autapomorphies:

Sichuanosuchus_shuhanensis :

All trees:

Char. 21: 0 --> 1

Char. 144: 1 --> 0

Sichuanosuchus_huidongensis :

All trees:

Char. 262: 0 --> 1

Shantungosuchus :

All trees:

No autapomorphies:

Zosuchus :

All trees:

Char. 31: 0 --> 1

Char. 43: 01 --> 2

Char. 80: 0 --> 1

Char. 105: 1 --> 2

Char. 107: 0 --> 3

Char. 139: 1 --> 0

Char. 159: 0 --> 1

Char. 164: 1 --> 0

Char. 172: 0 --> 1

Char. 192: 1 --> 0

Char. 261: 0 --> 1

Hsisosuchus_chowi :

All trees:

Char. 183: 0 --> 1

Hsisosuchus_chungkingensis :

All trees:

Char. 206: 0 --> 1

Fruitachampsa :

All trees:

Char. 11: 0 --> 1

Char. 18: 1 --> 0

Char. 39: 0 --> 1

Char. 66: 1 --> 2

Char. 91: 0 --> 1

Char. 92: 0 --> 1

Char. 126: 1 --> 0

Char. 134: 0 --> 1

Char. 141: 1 --> 0

Char. 265: 0 --> 1

Char. 365: 1 --> 0

Uruguaysuchus :

All trees:

Char. 166: 1 --> 0

Char. 175: 1 --> 0

Char. 272: 1 --> 0

Char. 277: 0 --> 1

Char. 278: 0 --> 1

Candidodon :

All trees:

Char. 107: 0 --> 1

Char. 119: 1 --> 0

Char. 127: 1 --> 0

Char. 139: 1 --> 0

Char. 148: 0 --> 1

Char. 399: 0 --> 1

Libycosuchus :

All trees:

Char. 75: 0 --> 1

Char. 191: 1 --> 0

Char. 211: 0 --> 1

Simosuchus :

All trees:

Char. 2: 1 --> 3

Char. 9: 2 --> 0

Char. 15: 1 --> 0

Char. 16: 1 --> 0

Char. 18: 0 --> 1

Char. 36: 2 --> 1

Char. 77: 1 --> 0

Char. 96: 0 --> 1

Char. 101: 1 --> 0

Char. 102: 1 --> 2

Char. 125: 0 --> 2

Char. 127: 1 --> 2

Char. 132: 0 --> 1

Char. 142: 1 --> 2

Char. 148: 0 --> 2

Char. 149: 1 --> 0

Char. 153: 0 --> 2

Char. 175: 1 --> 0

Char. 184: 0 --> 1

Char. 190: 0 --> 1

Char. 201: 0 --> 1

Char. 209: 0 --> 1

Char. 218: 0 --> 1

Char. 239: 0 --> 1

Char. 257: 0 --> 1

Char. 352: 0 --> 1

Char. 353: 0 --> 1

Char. 364: 0 --> 1

Char. 382: 0 --> 1

Char. 412: 0 --> 1

Char. 428: 0 --> 1

Char. 429: 0 --> 1

Malawisuchus :

All trees:

Char. 8: 0 --> 1

Char. 107: 03 --> 1

Char. 139: 1 --> 0

Char. 144: 1 --> 0

Char. 277: 0 --> 1

Pakasuchus :

All trees:

Char. 10: 0 --> 1

Char. 66: 1 --> 2

Char. 79: 0 --> 1

Char. 89: 2 --> 1

Char. 104: 2 --> 1

Char. 166: 0 --> 1

Chimaerasuchus :

All trees:

Char. 8: 0 --> 1

Char. 10: 0 --> 1

Char. 69: 1 --> 0

Char. 100: 0 --> 1

Char. 105: 1 --> 3

Char. 107: 03 --> 4

Char. 120: 0 --> 1

Char. 123: 0 --> 1

Char. 139: 1 --> 0

Char. 151: 1 --> 0

Char. 218: 0 --> 12

Char. 304: 1 --> 0

Char. 387: 0 --> 2

Char. 390: 0 --> 1

Notosuchus :

All trees:

Char. 11: 0 --> 1

Char. 30: 1 --> 0

Char. 54: 1 --> 0

Char. 94: 0 --> 1

Char. 105: 1 --> 0

Char. 129: 1 --> 0

Char. 131: 1 --> 0

Char. 135: 0 --> 1

Char. 141: 0 --> 1

Char. 147: 1 --> 0

Char. 161: 1 --> 0

Char. 164: 0 --> 1

Char. 179: 0 --> 1

Char. 194: 1 --> 0

Char. 208: 0 --> 1

Char. 236: 0 --> 1

Char. 298: 0 --> 1

Char. 307: 0 --> 1

Char. 349: 1 --> 0

Char. 352: 1 --> 0

Char. 353: 1 --> 0

Char. 365: 0 --> 1

Char. 366: 1 --> 0

Char. 377: 1 --> 0

Char. 383: 1 --> 0

Char. 384: 1 --> 0

Char. 391: 1 --> 0

Char. 439: 0 --> 1

Comahuesuchus :

All trees:

Char. 2: 1 --> 3

Char. 80: 0 --> 1

Char. 118: 1 --> 0

Char. 125: 0 --> 2

Char. 126: 1 --> 0

Char. 166: 0 --> 2

Char. 182: 0 --> 1

Char. 229: 0 --> 1

Char. 230: 0 --> 1

Char. 261: 0 --> 1

Char. 439: 0 --> 1

Mariliasuchus :

All trees:

Char. 28: 1 --> 0

Char. 107: 2 --> 3

Char. 144: 1 --> 0

Char. 147: 1 --> 0

Char. 166: 0 --> 12

Char. 228: 0 --> 1

Char. 230: 0 --> 1

Char. 236: 0 --> 1

Char. 261: 0 --> 1

Char. 292: 0 --> 1

Char. 347: 0 --> 1

Char. 359: 0 --> 1

Char. 381: 0 --> 1

Char. 383: 1 --> 0

Char. 392: 1 --> 0

Labidiosuchus :

All trees:

Char. 161: 1 --> 0

Char. 261: 0 --> 1

Char. 392: 1 --> 0

Caryonosuchus :

All trees:

Char. 105: 1 --> 3

Char. 134: 1 --> 0

Char. 363: 0 --> 1

Char. 380: 1 --> 0

Sphagesaurus :

All trees:

Char. 101: 1 --> 0

Char. 105: 1 --> 3

Char. 111: 1 --> 0

Char. 120: 0 --> 1

Char. 123: 0 --> 1

Char. 126: 0 --> 1

Char. 128: 0 --> 1

Char. 132: 0 --> 1

Char. 133: 0 --> 1

Char. 142: 1 --> 0

Char. 163: 1 --> 0

Char. 292: 0 --> 1

Char. 356: 1 --> 2

Char. 363: 0 --> 1

Char. 380: 1 --> 0

Char. 398: 0 --> 1

Armadillosuchus :

All trees:

Char. 21: 1 --> 0

Char. 30: 1 --> 0

Char. 94: 0 --> 1

Char. 96: 0 --> 1

Char. 105: 1 --> 3

Char. 111: 1 --> 0

Char. 123: 0 --> 1

Char. 126: 0 --> 1

Char. 134: 1 --> 0

Char. 142: 1 --> 0

Char. 163: 1 --> 0

Char. 167: 0 --> 1

Char. 180: 0 --> 1

Char. 210: 0 --> 1

Char. 266: 1 --> 0

Char. 323: 1 --> 0

Char. 354: 1 --> 0

Char. 356: 1 --> 2

Char. 363: 0 --> 1

Caipirasuchus_stenognathus :

All trees:

Char. 399: 0 --> 1

Caipirasuchus_montealtensis :

All trees:

Char. 292: 0 --> 1

Caipirasuchus_paulistanus :

All trees:

Char. 277: 0 --> 1

Char. 406: 1 --> 0

Caipirasuchus_mineirus :

All trees:

Char. 131: 1 --> 0

Char. 169: 1 --> 0

Char. 406: 1 --> 0

Yacarerani :

All trees:

Char. 21: 1 --> 0

Char. 28: 1 --> 0

Char. 73: 1 --> 0

Char. 90: 1 --> 5

Char. 120: 0 --> 1

Char. 225: 1 --> 0

Char. 230: 0 --> 1

Char. 261: 0 --> 1

Char. 270: 1 --> 0

Char. 277: 0 --> 1

Char. 296: 0 --> 1

Char. 301: 1 --> 0

Char. 332: 0 --> 1

Char. 347: 0 --> 1

Char. 356: 1 --> 0

Char. 359: 0 --> 1

Char. 364: 0 --> 1

Char. 382: 0 --> 1

Char. 384: 1 --> 0

Char. 385: 1 --> 0

Char. 390: 0 --> 1

Char. 404: 0 --> 1

Char. 412: 0 --> 1

Char. 428: 0 --> 1

Char. 429: 0 --> 1

Adamantinasuchus :

All trees:

Char. 121: 1 --> 0

Char. 129: 1 --> 0

Char. 215: 0 --> 1

Char. 225: 1 --> 0

Char. 228: 0 --> 1

Char. 230: 0 --> 1

Char. 261: 0 --> 1

Char. 364: 0 --> 1

Char. 365: 0 --> 1

Char. 384: 1 --> 0

Char. 385: 1 --> 0

Char. 390: 0 --> 1

Coringasuchus :

All trees:

Char. 235: 0 --> 2

Morrinhosuchus :

All trees:

No autapomorphies:

Campinasuchus :

All trees:

Char. 65: 1 --> 0

Char. 154: 0 --> 1

Char. 184: 0 --> 1

Char. 192: 0 --> 1

Char. 256: 1 --> 0

Char. 283: 1 --> 0

Pissarrachampsa :

All trees:

Char. 164: 1 --> 0

Char. 192: 0 --> 1

Char. 227: 2 --> 1

Char. 236: 0 --> 1

B._albertoi :

All trees:

No autapomorphies:

B._pachecoi :

All trees:

Char. 73: 0 --> 1

B._salgadoensis :

All trees:

No autapomorphies:

Stratiotosuchus :

All trees:

Char. 213: 0 --> 1

Char. 356: 1 --> 0

Char. 419: 1 --> 0

Pehuenchesuchus :

All trees:

Char. 79: 1 --> 0

Char. 119: 0 --> 1

Cynodontosuchus :

All trees:

Char. 128: 0 --> 1

Char. 360: 0 --> 1

Char. 405: 0 --> 1

Char. 409: 0 --> 1

Bergisuchus :

All trees:

No autapomorphies:

Iberosuchus :

All trees:

No autapomorphies:

Bretesuchus :

All trees:

Char. 226: 0 --> 1

Barinasuchus :

All trees:

No autapomorphies:

S._huilensis :

All trees:

No autapomorphies:

S._icaeorhinus :

All trees:

Char. 395: 0 --> 1

S._querejazus :

All trees:

Char. 41: 1 --> 0

Char. 68: 0 --> 1

Char. 178: 1 --> 0

Ayllusuchus :

All trees:

No autapomorphies:

Lorosuchus :

All trees:

Char. 2: 0 --> 3

Char. 5: 0 --> 2

Char. 8: 2 --> 1

Char. 24: 1 --> 0

Char. 41: 1 --> 0

Char. 65: 1 --> 0

Char. 76: 1 --> 2

Char. 102: 2 --> 3

Char. 119: 0 --> 1

Char. 134: 1 --> 0

Char. 141: 0 --> 1

Char. 155: 0 --> 1

Char. 159: 0 --> 1

Char. 161: 0 --> 1

Char. 225: 2 --> 0

Char. 265: 0 --> 1

Char. 285: 0 --> 1

Char. 367: 1 --> 2

Char. 398: 1 --> 0

Char. 406: 1 --> 0

Lumbrera_form :

All trees:

Char. 120: 0 --> 1

Pabhwehshi :

All trees:

Char. 77: 0 --> 1

Char. 105: 1 --> 2

Char. 236: 0 --> 1

Char. 408: 0 --> 1

A._gomesii :

All trees:

Char. 166: 1 --> 2

Char. 185: 1 --> 0

A._patagonicus :

All trees:

No autapomorphies:

A._buitreraensis :

All trees:

Char. 22: 1 --> 0

Char. 164: 0 --> 1

Char. 197: 0 --> 1

Char. 292: 0 --> 1

A._wegeneri :

All trees:

Char. 21: 0 --> 1

Char. 119: 1 --> 0

Char. 211: 0 --> 1

Char. 225: 0 --> 1

Char. 264: 1 --> 0

Char. 318: 0 --> 1

A._tsangatsangana :

All trees:

Char. 64: 3 --> 2

Char. 140: 0 --> 1

Char. 166: 1 --> 2

Char. 173: 0 --> 1

Char. 197: 0 --> 1

Char. 367: 1 --> 2

Anatosuchus :

All trees:

Char. 2: 1 --> 3

Char. 21: 0 --> 1

Char. 127: 1 --> 0

Char. 139: 1 --> 0

Char. 166: 1 --> 2

Char. 170: 1 --> 0

Char. 184: 0 --> 1

Char. 185: 1 --> 0

Char. 234: 0 --> 1

Char. 239: 0 --> 1

Char. 406: 0 --> 1

Montealtosuchus :

All trees:

Char. 22: 1 --> 0

Char. 30: 0 --> 1

Char. 134: 0 --> 1

Char. 154: 0 --> 1

Uberabasuchus :

All trees:

Char. 73: 0 --> 1

Char. 101: 1 --> 0

Char. 104: 1 --> 2

Lomasuchus :

All trees:

No autapomorphies:

Gasparinisuchus :

All trees:

Char. 72: 0 --> 1

Hamadasuchus :

All trees:

Char. 77: 0 --> 1

Char. 78: 1 --> 2

Char. 105: 0 --> 1

Char. 129: 0 --> 1

Char. 347: 0 --> 1

Char. 399: 0 --> 1

Char. 408: 0 --> 1

Mahajangasuchus :

All trees:

Char. 17: 0 --> 1

Char. 73: 0 --> 1

Char. 140: 0 --> 1

Char. 166: 1 --> 2

Char. 180: 0 --> 1

Char. 265: 0 --> 1

Char. 285: 1 --> 0

Char. 376: 1 --> 0

Char. 400: 0 --> 1

Kaprosuchus :

All trees:

Char. 21: 0 --> 1

Char. 69: 1 --> 0

Char. 77: 0 --> 1

Char. 78: 1 --> 2

Char. 199: 0 --> 1

Char. 208: 0 --> 1

Char. 270: 0 --> 1

Stolokrosuchus :

All trees:

Char. 6: 1 --> 0

Char. 28: 1 --> 0

Char. 76: 2 --> 3

Char. 104: 1 --> 0

Char. 123: 0 --> 1

Char. 129: 0 --> 1

Char. 146: 0 --> 1

Char. 147: 0 --> 1

Char. 159: 0 --> 1

Char. 160: 0 --> 1

Char. 166: 1 --> 2

Char. 169: 0 --> 1

Char. 183: 0 --> 1

Char. 185: 1 --> 0

Char. 245: 0 --> 1

Char. 282: 0 --> 1

Char. 284: 1 --> 0

Char. 294: 1 --> 2

Char. 368: 1 --> 0

Char. 385: 0 --> 1

Char. 408: 0 --> 1

Theriosuchus :

All trees:

Char. 11: 0 --> 1

Char. 21: 0 --> 1

Alligatorium :

All trees:

No autapomorphies:

Goniopholis_simus :

All trees:

Char. 384: 0 --> 1

Char. 385: 0 --> 1

Goniopholis_stovalli :

All trees:

Char. 105: 0 --> 1

Eutretauranosuchus :

All trees:

Char. 270: 0 --> 2

Sunosuchus :

All trees:

Char. 10: 0 --> 1

Char. 267: 0 --> 1

Char. 275: 0 --> 3

Shamosuchus :

All trees:

Char. 10: 0 --> 1

Char. 78: 2 --> 1

Char. 121: 1 --> 0

Char. 183: 1 --> 0

Char. 219: 0 --> 1

Char. 276: 0 --> 1

Bernissartia :

All trees:

Char. 263: 0 --> 1

Char. 279: 0 --> 1

Hylaeochampsa :

All trees:

Char. 0: 2 --> 0

Char. 10: 0 --> 1

Char. 183: 1 --> 2

Glen_Rose_Form :

All trees:

Char. 5: 2 --> 0

Char. 30: 0 --> 1

Char. 65: 1 --> 0

Char. 79: 0 --> 1

Char. 183: 1 --> 0

Char. 218: 0 --> 2

Char. 261: 0 --> 2

Char. 275: 0 --> 1

Char. 395: 0 --> 1

Calsoyasuchus :

All trees:

Char. 22: 0 --> 2

Char. 66: 2 --> 1

Borealosuchus :

All trees:

Char. 12: 0 --> 1

Char. 49: 0 --> 1

Char. 88: 0 --> 1

Pristichampsus_vorax :

All trees:

Char. 2: 3 --> 0

Char. 63: 0 --> 1

Char. 119: 1 --> 0

Char. 139: 0 --> 1

Char. 146: 1 --> 0

Char. 159: 1 --> 0

Char. 180: 0 --> 1

Eothoracosaurus_mississippiensi :

All trees:

Char. 22: 1 --> 0

Char. 100: 0 --> 1

Gavialis :

All trees:

Char. 1: 0 --> 1

Char. 8: 1 --> 0

Char. 13: 0 --> 1

Char. 255: 0 --> 1

Char. 265: 0 --> 1

Char. 279: 0 --> 1

Leidyosuchus_canadensis :

All trees:

Char. 22: 1 --> 0

Char. 49: 0 --> 1

Asiatosuchus_germanicus :

All trees:

Char. 116: 0 --> 1

Crocodylus :

All trees:

Char. 22: 1 --> 2

Char. 76: 2 --> 0

Char. 378: 0 --> 1

Diplocynodon_hantoniensis :

All trees:

Char. 12: 0 --> 1

Alligator :

All trees:

Char. 8: 1 --> 0

Char. 22: 1 --> 2

Char. 65: 1 --> 0

Char. 68: 0 --> 2

Char. 88: 0 --> 1

Char. 99: 1 --> 0

Char. 143: 0 --> 1

Char. 265: 0 --> 1

Pelagosaurus :

All trees:

Char. 19: 0 --> 1

Char. 24: 1 --> 0

Char. 58: 1 --> 0

Steneosaurus_bollensis :

All trees:

Char. 208: 0 --> 1

M._superciliousus :

All trees:

Char. 243: 1 --> 0

Char. 361: 0 --> 1

M._casamequelai :

All trees:

Char. 43: 0 --> 1

Char. 241: 0 --> 1

C._araucaniensis :

All trees:

No autapomorphies:

C._suevicus :

All trees:

No autapomorphies:

D._maximus :

All trees:

No autapomorphies:

D._andiniensis :

All trees:

No autapomorphies:

Rhabdognathus :

All trees:

Char. 263: 1 --> 0

Sokotosuchus :

All trees:

Char. 8: 0 --> 1

Char. 78: 0 --> 1

Char. 177: 0 --> 1

Dyrosaurus :

All trees:

Char. 166: 2 --> 0

Hyposaurus :

All trees:

No autapomorphies:

Pholidosaurus :

All trees:

Char. 1: 0 --> 1

Sarcosuchus :

All trees:

Char. 2: 2 --> 3

Char. 79: 0 --> 1

Char. 99: 1 --> 0

Char. 277: 0 --> 1

Char. 385: 1 --> 0

Terminonaris :

All trees:

No autapomorphies:

Rugosuchus :

All trees:

Char. 94: 1 --> 2

Char. 127: 1 --> 0

Char. 256: 0 --> 1

Char. 279: 0 --> 1

Char. 385: 0 --> 1

Argochampsa :

All trees:

Char. 24: 1 --> 0

Char. 35: 0 --> 1

Char. 67: 1 --> 0

Char. 182: 1 --> 0

Char. 233: 1 --> 0

Char. 256: 0 --> 1

Microsuchus :

All trees:

Char. 115: 1 --> 2

Char. 339: 0 --> 1

Char. 438: 0 --> 1

Neuquensuchus :

All trees:

Char. 316: 0 --> 1

Char. 438: 0 --> 1

Llanosuchus :

All trees:

Char. 161: 1 --> 0

Char. 179: 0 --> 1

Char. 235: 0 --> 1

Char. 383: 1 --> 0

Char. 391: 1 --> 0

Node 114 :

All trees:

Char. 32: 0 --> 1

Char. 127: 1 --> 0

Node 115 :

All trees:

No synapomorphies

Node 116 :

All trees:

Char. 16: 0 --> 1

Char. 134: 0 --> 1

Char. 211: 0 --> 1

Node 117 :

All trees:

Char. 1: 0 --> 1

Char. 57: 0 --> 1

Char. 99: 0 --> 1

Char. 215: 0 --> 1

Node 118 :

All trees:

Char. 49: 0 --> 1

Char. 72: 1 --> 2

Char. 73: 0 --> 1

Char. 90: 0 --> 12

Char. 102: 3 --> 0

Node 119 :

All trees:

Char. 0: 0 --> 2

Char. 15: 0 --> 1

Char. 18: 0 --> 1

Char. 23: 0 --> 1

Char. 29: 0 --> 1

Char. 44: 0 --> 2

Char. 46: 0 --> 1

Char. 50: 0 --> 1

Char. 54: 0 --> 1

Char. 66: 0 --> 1

Char. 67: 0 --> 1

Char. 77: 0 --> 1

Char. 81: 0 --> 1

Char. 94: 0 --> 1

Char. 98: 0 --> 1

Char. 121: 1 --> 0

Char. 195: 0 --> 1

Char. 196: 0 --> 2

Char. 203: 0 --> 1

Char. 204: 0 --> 1

Char. 251: 0 --> 1

Char. 328: 0 --> 1

Node 120 :

All trees:

Char. 85: 0 --> 1

Char. 116: 0 --> 1

Char. 133: 1 --> 0

Char. 138: 0 --> 1

Char. 323: 0 --> 1

Node 121 :

All trees:

Char. 8: 3 --> 2

Char. 38: 0 --> 1

Char. 107: 0 --> 2

Char. 159: 0 --> 1

Char. 161: 0 --> 1

Char. 187: 0 --> 1

Char. 194: 0 --> 1

Char. 209: 0 --> 1

Char. 389: 0 --> 1

Node 122 :

All trees:

Char. 0: 2 --> 1

Char. 31: 0 --> 1

Char. 74: 0 --> 1

Char. 96: 0 --> 1

Char. 205: 1 --> 0

Char. 213: 0 --> 1

Char. 214: 0 --> 1

Char. 215: 0 --> 1

Char. 216: 0 --> 1

Char. 218: 0 --> 1

Char. 219: 0 --> 1

Char. 220: 0 --> 1

Char. 221: 0 --> 1

Char. 222: 0 --> 1

Char. 223: 0 --> 1

Node 123 :

All trees:

Char. 8: 3 --> 1

Char. 35: 0 --> 2

Char. 48: 0 --> 2

Char. 84: 0 --> 1

Char. 99: 0 --> 1

Char. 106: 1 --> 0

Char. 163: 0 --> 1

Char. 268: 0 --> 1

Node 124 :

All trees:

Char. 77: 1 --> 0

Char. 137: 0 --> 1

Char. 163: 1 --> 0

Char. 201: 0 --> 1

Char. 205: 1 --> 0

Char. 215: 0 --> 1

Char. 218: 0 --> 1

Char. 219: 0 --> 1

Node 125 :

All trees:

Char. 142: 1 --> 2

Char. 200: 0 --> 1

Char. 210: 0 --> 1

Node 126 :

All trees:

Char. 8: 1 --> 2

Char. 30: 0 --> 1

Node 127 :

All trees:

Char. 36: 0 --> 12

Char. 38: 0 --> 1

Char. 40: 0 --> 1

Char. 78: 0 --> 1

Char. 102: 3 --> 0

Char. 124: 0 --> 1

Char. 149: 0 --> 1

Char. 191: 0 --> 1

Char. 196: 2 --> 1

Node 128 :

All trees:

Char. 15: 1 --> 0

Char. 101: 1 --> 0

Char. 106: 0 --> 1

Char. 127: 1 --> 0

Node 129 :

All trees:

Char. 16: 0 --> 1

Char. 45: 0 --> 1

Char. 72: 1 --> 0

Char. 76: 0 --> 2

Char. 77: 1 --> 0

Char. 118: 0 --> 1

Char. 121: 0 --> 1

Char. 147: 1 --> 0

Char. 167: 0 --> 1

Char. 196: 1 --> 0

Char. 202: 1 --> 0

Char. 233: 0 --> 1

Char. 374: 0 --> 1

Node 130 :

All trees:

Char. 182: 1 --> 0

Char. 192: 0 --> 1

Node 131 :

All trees:

Char. 30: 0 --> 1

Char. 224: 2 --> 1

Node 132 :

All trees:

Char. 190: 0 --> 1

Char. 270: 0 --> 1

Node 133 :

All trees:

Char. 26: 0 --> 1

Char. 75: 0 --> 1

Char. 142: 1 --> 2

Char. 144: 1 --> 0

Char. 148: 0 --> 1

Char. 158: 0 --> 1

Char. 186: 0 --> 1

Char. 196: 0 --> 1

Char. 208: 0 --> 1

Char. 399: 0 --> 1

Node 134 :

All trees:

Char. 25: 0 --> 1

Char. 272: 0 --> 1

Char. 283: 0 --> 1

Char. 294: 0 --> 1

Char. 306: 0 --> 1

Char. 335: 0 --> 1

Char. 337: 0 --> 1

Char. 339: 0 --> 1

Char. 436: 0 --> 1

Node 135 :

All trees:

Char. 22: 0 --> 1

Char. 69: 0 --> 1

Char. 89: 0 --> 1

Char. 151: 0 --> 1

Char. 164: 1 --> 0

Char. 170: 0 --> 1

Char. 185: 0 --> 1

Char. 295: 0 --> 1

Char. 301: 0 --> 1

Char. 302: 0 --> 1

Char. 304: 0 --> 1

Char. 313: 0 --> 1

Char. 325: 0 --> 1

Char. 326: 0 --> 1

Char. 327: 0 --> 1

Char. 342: 1 --> 2

Char. 345: 1 --> 0

Char. 355: 1 --> 2

Char. 367: 0 --> 1

Char. 368: 0 --> 1

Char. 378: 0 --> 1

Char. 413: 0 --> 1

Char. 414: 0 --> 1

Char. 425: 0 --> 1

Char. 427: 0 --> 1

Node 136 :

All trees:

Char. 9: 0 --> 2

Char. 14: 0 --> 1

Char. 18: 1 --> 0

Char. 44: 2 --> 1

Char. 70: 0 --> 1

Char. 82: 0 --> 1

Char. 198: 0 --> 1

Node 137 :

All trees:

Char. 77: 0 --> 1

Char. 102: 0 --> 1

Char. 169: 0 --> 1

Char. 225: 0 --> 1

Node 138 :

All trees:

Char. 104: 1 --> 2

Char. 106: 0 --> 1

Node 139 :

All trees:

Char. 0: 2 --> 1

Char. 73: 0 --> 1

Char. 186: 0 --> 1

Char. 359: 1 --> 0

Char. 375: 0 --> 1

Node 140 :

All trees:

Char. 105: 0 --> 1

Char. 140: 0 --> 1

Char. 167: 1 --> 0

Char. 192: 1 --> 2

Char. 194: 0 --> 1

Char. 305: 0 --> 1

Char. 365: 1 --> 0

Node 141 :

All trees:

Char. 103: 1 --> 0

Char. 153: 0 --> 1

Char. 163: 1 --> 0

Char. 192: 2 --> 1

Char. 225: 1 --> 2

Node 142 :

All trees:

Char. 41: 0 --> 1

Char. 76: 2 --> 1

Char. 114: 0 --> 1

Char. 119: 1 --> 2

Char. 231: 0 --> 1

Char. 278: 0 --> 1

Char. 310: 0 --> 1

Node 143 :

All trees:

Char. 136: 0 --> 1

Char. 383: 0 --> 1

Node 144 :

All trees:

Char. 107: 03 --> 2

Char. 126: 1 --> 0

Char. 362: 0 --> 1

Char. 380: 0 --> 1

Node 145 :

All trees:

Char. 66: 1 --> 2

Char. 119: 2 --> 0

Char. 130: 0 --> 1

Char. 158: 0 --> 2

Char. 283: 0 --> 1

Node 146 :

All trees:

Char. 126: 0 --> 1

Char. 144: 1 --> 0

Char. 208: 0 --> 1

Char. 225: 1 --> 0

Char. 350: 0 --> 1

Char. 363: 0 --> 2

Char. 364: 0 --> 1

Char. 382: 0 --> 1

Char. 398: 0 --> 1

Char. 411: 0 --> 1

Node 147 :

All trees:

Char. 101: 1 --> 0

Char. 224: 2 --> 1

Char. 352: 1 --> 0

Node 148 :

All trees:

Char. 8: 2 --> 3

Char. 137: 0 --> 1

Char. 226: 0 --> 1

Node 149 :

All trees:

Char. 353: 0 --> 1

Char. 400: 1 --> 2

Node 150 :

All trees:

Char. 18: 0 --> 1

Char. 32: 0 --> 1

Char. 35: 2 --> 3

Char. 43: 0 --> 1

Char. 44: 1 --> 2

Char. 120: 0 --> 1

Char. 128: 0 --> 1

Char. 133: 0 --> 1

Char. 135: 0 --> 1

Char. 140: 0 --> 1

Char. 142: 1 --> 2

Char. 148: 0 --> 1

Char. 178: 1 --> 0

Char. 179: 0 --> 1

Char. 191: 0 --> 1

Char. 211: 1 --> 0

Char. 232: 0 --> 1

Char. 256: 0 --> 1

Char. 289: 0 --> 1

Char. 290: 0 --> 1

Char. 349: 0 --> 1

Char. 360: 0 --> 1

Char. 361: 0 --> 1

Char. 397: 0 --> 1

Char. 399: 0 --> 1

Char. 400: 0 --> 1

Char. 401: 0 --> 1

Char. 402: 0 --> 1

Char. 404: 0 --> 1

Char. 405: 0 --> 1

Node 151 :

All trees:

Char. 2: 1 --> 0

Char. 8: 0 --> 2

Char. 79: 0 --> 1

Char. 106: 1 --> 0

Char. 117: 0 --> 1

Char. 129: 0 --> 1

Char. 137: 1 --> 0

Char. 154: 1 --> 0

Char. 167: 0 --> 1

Char. 180: 0 --> 1

Char. 191: 1 --> 0

Char. 192: 1 --> 0

Char. 211: 0 --> 1

Char. 227: 1 --> 2

Char. 267: 0 --> 1

Char. 398: 0 --> 1

Node 152 :

All trees:

Char. 398: 1 --> 0

Node 153 :

All trees:

Char. 66: 2 --> 1

Char. 138: 1 --> 0

Char. 160: 0 --> 1

Node 154 :

All trees:

Char. 284: 1 --> 2

Char. 408: 0 --> 1

Node 155 :

All trees:

Char. 287: 0 --> 1

Node 156 :

All trees:

Char. 68: 1 --> 0

Char. 138: 1 --> 0

Node 157 :

All trees:

Char. 8: 2 --> 1

Char. 139: 1 --> 2

Node 158 :

All trees:

Char. 212: 0 --> 1

Char. 213: 0 --> 1

Char. 282: 0 --> 1

Node 159 :

All trees:

Char. 119: 1 --> 0

Char. 127: 1 --> 0

Char. 170: 1 --> 0

Char. 236: 0 --> 1

Char. 242: 01 --> 2

Char. 395: 0 --> 1

Node 160 :

All trees:

Char. 76: 2 --> 1

Char. 158: 0 --> 3

Char. 358: 0 --> 1

Node 161 :

All trees:

Char. 163: 1 --> 0

Char. 191: 1 --> 0

Char. 211: 0 --> 1

Char. 226: 0 --> 1

Char. 285: 0 --> 1

Node 162 :

All trees:

Char. 0: 2 --> 1

Char. 2: 1 --> 3

Char. 5: 0 --> 12

Char. 35: 12 --> 4

Char. 42: 0 --> 1

Char. 75: 0 --> 1

Char. 102: 0 --> 2

Char. 104: 1 --> 2

Char. 125: 01 --> 2

Char. 142: 1 --> 2

Char. 153: 0 --> 2

Char. 190: 0 --> 1

Char. 256: 0 --> 1

Char. 293: 0 --> 1

Char. 356: 0 --> 1

Char. 365: 1 --> 2

Char. 397: 0 --> 1

Char. 398: 0 --> 1

Node 163 :

All trees:

Char. 16: 1 --> 0

Char. 30: 0 --> 1

Char. 34: 0 --> 1

Char. 82: 1 --> 0

Char. 99: 1 --> 0

Node 164 :

All trees:

Char. 2: 2 --> 3

Char. 25: 0 --> 1

Char. 80: 0 --> 1

Char. 143: 1 --> 0

Char. 177: 0 --> 1

Char. 182: 0 --> 1

Char. 263: 1 --> 0

Node 165 :

All trees:

Char. 2: 1 --> 2

Char. 5: 0 --> 1

Char. 35: 2 --> 0

Char. 64: 3 --> 2

Char. 111: 1 --> 0

Char. 115: 1 --> 0

Char. 146: 0 --> 1

Char. 149: 1 --> 0

Char. 152: 0 --> 1

Char. 159: 0 --> 1

Char. 163: 1 --> 0

Char. 191: 1 --> 0

Char. 211: 0 --> 1

Char. 316: 0 --> 1

Char. 355: 1 --> 0

Char. 357: 0 --> 1

Node 166 :

All trees:

Char. 12: 0 --> 1

Char. 68: 1 --> 2

Char. 100: 0 --> 1

Char. 160: 0 --> 1

Char. 206: 0 --> 1

Char. 399: 0 --> 1

Node 167 :

All trees:

Char. 5: 1 --> 2

Char. 55: 0 --> 1

Char. 66: 1 --> 2

Char. 78: 1 --> 2

Char. 104: 1 --> 0

Char. 168: 0 --> 1

Char. 172: 0 --> 1

Char. 277: 0 --> 1

Node 168 :

All trees:

Char. 275: 0 --> 2

Node 169 :

All trees:

Char. 9: 2 --> 1

Node 170 :

All trees:

Char. 6: 1 --> 0

Char. 78: 2 --> 1

Node 171 :

All trees:

Char. 21: 0 --> 1

Char. 58: 1 --> 0

Char. 218: 0 --> 2

Char. 278: 0 --> 1

Node 172 :

All trees:

Char. 89: 0 --> 1

Char. 90: 2 --> 3

Char. 91: 0 --> 1

Char. 164: 1 --> 0

Node 173 :

All trees:

Char. 42: 0 --> 1

Char. 68: 1 --> 0

Char. 93: 0 --> 1

Char. 95: 2 --> 1

Char. 96: 0 --> 1

Node 174 :

All trees:

Char. 268: 1 --> 0

Node 175 :

All trees:

Char. 42: 1 --> 2

Char. 43: 01 --> 2

Char. 264: 0 --> 1

Char. 277: 1 --> 0

Node 176 :

All trees:

Char. 267: 0 --> 1

Node 177 :

All trees:

Char. 22: 0 --> 1

Char. 259: 0 --> 1

Node 178 :

All trees:

Char. 237: 0 --> 1

Node 179 :

All trees:

Char. 2: 3 --> 2

Char. 12: 0 --> 1

Char. 19: 0 --> 1

Char. 56: 0 --> 1

Char. 78: 2 --> 0

Char. 161: 1 --> 0

Char. 177: 1 --> 0

Char. 242: 0 --> 1

Char. 266: 1 --> 0

Char. 269: 1 --> 0

Char. 359: 1 --> 0

Node 180 :

All trees:

Char. 70: 1 --> 2

Char. 90: 3 --> 4

Char. 99: 1 --> 0

Char. 257: 0 --> 1

Node 181 :

All trees:

Char. 94: 1 --> 2

Char. 164: 0 --> 1

Node 182 :

All trees:

Char. 3: 0 --> 1

Char. 263: 0 --> 1

Char. 277: 0 --> 1

Char. 294: 0 --> 1

Node 183 :

All trees:

Char. 76: 2 --> 0

Char. 168: 1 --> 0

Char. 278: 0 --> 1

Node 184 :

All trees:

Char. 117: 0 --> 1

Char. 247: 0 --> 1

Node 185 :

All trees:

Char. 13: 0 --> 1

Char. 15: 1 --> 2

Char. 16: 1 --> 0

Char. 23: 1 --> 0

Char. 29: 1 --> 0

Char. 32: 0 --> 1

Char. 45: 1 --> 0

Char. 46: 1 --> 0

Char. 48: 2 --> 1

Char. 59: 0 --> 1

Char. 64: 1 --> 0

Char. 67: 1 --> 0

Char. 83: 1 --> 0

Char. 85: 2 --> 1

Char. 165: 0 --> 1

Char. 174: 0 --> 1

Char. 178: 0 --> 2

Char. 196: 0 --> 1

Char. 205: 1 --> 0

Char. 227: 0 --> 2

Char. 243: 0 --> 1

Char. 248: 0 --> 1

Char. 266: 1 --> 0

Char. 324: 0 --> 1

Node 186 :

All trees:

Char. 12: 0 --> 1

Char. 44: 1 --> 0

Char. 62: 1 --> 0

Char. 64: 2 --> 1

Char. 76: 2 --> 3

Char. 78: 1 --> 0

Char. 116: 1 --> 0

Char. 294: 0 --> 1

Char. 376: 1 --> 0

Char. 385: 0 --> 1

Node 187 :

All trees:

Char. 0: 2 --> 0

Char. 74: 0 --> 1

Char. 101: 1 --> 0

Char. 105: 1 --> 2

Char. 110: 0 --> 1

Char. 171: 1 --> 0

Char. 245: 0 --> 1

Char. 246: 0 --> 1

Char. 249: 0 --> 1

Char. 250: 0 --> 1

Node 188 :

All trees:

Char. 65: 1 --> 0

Node 189 :

All trees:

Char. 68: 1 --> 0

Char. 130: 0 --> 1

Char. 244: 0 --> 1

Char. 253: 0 --> 1

Char. 385: 1 --> 0

Node 190 :

All trees:

Char. 2: 2 --> 1

Char. 119: 1 --> 0

Char. 139: 0 --> 1

Node 191 :

All trees:

Char. 251: 1 --> 0

Node 192 :

All trees:

Char. 32: 0 --> 1

Char. 63: 0 --> 1

Char. 67: 1 --> 0

Char. 127: 1 --> 0

Char. 256: 0 --> 1

Node 193 :

All trees:

Char. 27: 0 --> 1

Char. 255: 0 --> 1

Node 194 :

All trees:

Char. 5: 1 --> 2

Char. 35: 0 --> 1

Char. 42: 0 --> 1

Char. 55: 0 --> 1

Char. 66: 1 --> 2

Char. 237: 0 --> 1

Node 195 :

All trees:

Char. 77: 0 --> 1

Char. 162: 0 --> 1

Char. 238: 0 --> 1

Char. 239: 0 --> 1

Char. 240: 0 --> 1

Char. 264: 0 --> 1
